# Design, Synthesis and Biological Evaluation of Neogliptin, a Novel 2-Azabicyclo[2.2.1]heptane-Based Inhibitor of Dipeptidyl Peptidase-4 (DPP-4)

**DOI:** 10.3390/ph15030273

**Published:** 2022-02-22

**Authors:** Ivan O. Maslov, Tatiana V. Zinevich, Olga G. Kirichenko, Mikhail V. Trukhan, Sergey V. Shorshnev, Natalya O. Tuaeva, Maxim A. Gureev, Amelia D. Dahlén, Yuri B. Porozov, Helgi B. Schiöth, Vladimir M. Trukhan

**Affiliations:** 1 Department of Bioorganic chemistry, Faculty of Biology, Lomonosov Moscow State University, 119991 Moscow, Russia; ivan.maslov@monash.edu (I.O.M.); zinevich.tatiana@gmail.com (T.V.Z.); 2 LLC Institute of Mitoengineering MSU, 119899 Moscow, Russia; kirolga2001@mail.ru; 3 Institute for Translational Medicine and Biotechnology, I. M. Sechenov First Moscow State Medical University, 119991 Moscow, Russia; trukhan.misha@gmail.com (M.V.T.); natalya.tuaeva@gmail.com (N.O.T.); 4 Chembridge Corp., 119435 Moscow, Russia; gray20002006@yandex.ru; 5 Serbsky Institute for General and Forensic Psychiatry, 119839 Moscow, Russia; 6 World-Class Research Centre “Digital Biodesign and personalised healthcare”, I. M. Sechenov First Moscow State Medical University, 119991 Moscow, Russia; max_technik@mail.ru (M.A.G.); yuri.porozov@gmail.com (Y.B.P.); 7 Department of Computational Biology, Sirius University of Science and Technology, Olympic Ave 1, 354340 Sochi, Russia; 8 Functional Pharmacology and Neuroscience, Department of Surgical Sciences, Uppsala University, 75124 Uppsala, Sweden; dahlenamelia@gmail.com

**Keywords:** type 2 diabetes mellitus, DPP-4 inhibitors, molecular docking, structure-activity relationship, stereoisomerism, rotameric forms

## Abstract

Compounds that contain (R)-3-amino-4-(2,4,5-trifluorophenyl)butanoic acid substituted with bicyclic amino moiety (2-aza-bicyclo[2.2.1]heptane) were designed using molecular modelling methods, synthesised, and found to be potent DPP-4 (dipeptidyl peptidase-4) inhibitors. Compound **12a** (IC50 = 16.8 ± 2.2 nM), named neogliptin, is a more potent DPP-4 inhibitor than vildagliptin and sitagliptin. Neogliptin interacts with key DPP-4 residues in the active site and has pharmacophore parameters similar to vildagliptin and sitagliptin. It was found to have a low cardiotoxic effect compared to sitagliptin, and it is superior to vildagliptin in terms of ADME properties. Moreover, compound **12a** is stable in aqueous solutions due to its low intramolecular cyclisation potential. These findings suggest that compound **12a** has unique properties and can act as a template for further type 2 diabetes mellitus drug development.

## 1. Introduction

Pharmacological therapy for type 2 diabetes mellitus (T2DM) patients has developed considerably over the past decades involving several new strategies [1,2,3]. Many of these strategies involve more patient-friendly ways of drug administration. Several decades ago, insulin injections were the only way to overcome hyperglycaemia, but now many oral antihyperglycaemic agents are available that are administrated either in monotherapy or as combinational drug therapy. Currently, one of the most recent and promising methods to treat T2DM is to use dipeptidyl peptidase 4 (DPP-4) inhibitors, which are also called gliptins [4,5,6,7,8,9]. They prevent the degradation of glucagon-like peptide-1 (GLP-1) and glucose-dependent insulinotropic peptide (GIP), stimulate insulin synthesis, suppress glucagon secretion, inhibit appetite, reduce body weight, slow gastric emptying, and can restore functions of pancreatic beta cells providing a new approach that is effective for T2DM patients.

DPP-4 is a glycoprotein expressed in several tissues that has a catalytic activity selective for protein molecules that contain Pro or Ala in the penultimate position at the N-terminus; it plays a vital role in several functions, including immune regulation, signal transduction, and apoptosis as well as being used by MERS-CoV as a receptor [10]. DPP-4 consists of 766 amino acids (aa) and comprises several parts: intracellular (6 aa), transmembrane (22 aa), and large extracellular (738 aa) with a catalytic site. Two identical DPP-4 glycoproteins are usually located near each other and form a dimer. Each subunit is composed of *α/β*-hydrolase and *β*-propeller domains, and they form a cavity that is crucial for inhibitor binding [6,11].

Currently, several structures oriented to target-specific interaction with DPP-4 are already known and officially approved by the U.S. Food & Drug Administration (FDA), including sitagliptin [12], saxagliptin [13], alogliptin [14], and linagliptin [15] (Table 1). All these DPP-4 inhibitors are used both in monotherapy, such as Januvia (sitagliptin), Onglyza (saxagliptin), Nesina/Vipidia (alogliptin), Trajenta (linagliptin), and in combinations with other antihyperglycaemic drugs. The FDA-approved combinations with DPP-4 inhibitors include Janumet (sitagliptin and metformin), Jentadueto (linagliptin and metformin), Kazano (alogliptin and metformin), Steglujan (sitagliptin and ertugliflozin), Qtern (saxagliptin and dapagliflozin), Glyxambi (linagliptin and empagliflozin), Oseni (alogliptin and pioglitazone) and Qternmet XR (saxagliptin, metformin, and dapagliflozin).

Some other gliptins (e.g., vildagliptin, teneligliptin, trelagliptin, evogliptin, omarigliptin) have not been yet approved by the FDA (Table 1), but they are nevertheless marketed in many countries and used as T2DM treatment. Moreover, several DPP-4 inhibitors are in the final phases of clinical trials. In recent years, studies of drugs’ physiological profiles and clinical properties based on DPP-4 inhibitors have been intensively and widely conducted and the side effects that such drugs may cause. Although the four approved DPP-4 inhibitors share good properties, such as antihyperglycaemic activity, the lack of bodyweight influence, and low risk of hypoglycaemia, some specific side effects such as hepatotoxicity or cardiotoxicity [16], and adverse reactions should be considered. Moreover, the importance of providing individualised patient care for safe and effective antihyperglycaemic therapies has also been highlighted. That is why there is still strong enthusiasm in developing novel inhibitors of DPP-4 with more favourable side effects profiles, different pharmacokinetic properties, or cheaper to manufacture.

Over the last years, several new DPP-4 inhibitors have been published, many of which are very potent in the low nanomolar range of IC50 values. Different scaffolds have been closely studied to search for new active DPP-4 inhibitors. In silico methods are widely used at the early stages of drug design as they provide an opportunity to reduce the number of molecules needed to be screened [8,17]. Some strategies of DPP-4 inhibitor design, like ligand-based and structure-based virtual screening, utilise computer-aided drug discovery methods, allowing to prioritise the space of small molecules, using a series of patterns, calculated in accordance to protein active site structure data and its ligands [11,18,19,20,21,22,23,24,25,26]. Many experiments were carried out on the structural characterisation of synthesised or natural compounds using high-throughput methods.

Here we performed virtual screening and created structure-activity relationship (SAR) models to reveal new potent DPP-4 inhibitors. In this article, we present a novel class of small-molecule derivatives based on 3-azabicyclo[2.2.1]heptene-2-carbonitrile that seems to be promising agents to treat hyperglycaemia.

## 2. Results and Discussion

### 2.1. Molecular Modelling

**Figure 1:**
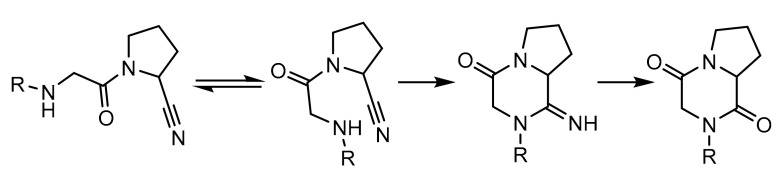
Spontaneous intramolecular cyclisation of the cyanopyrrolidine derivatives acylated with alpha-amino acids.

We initially analysed analogous compounds that contain the same beta-amino acid ((R)-3-amino 4-(2,4,5-trifluorophenyl)butanoic acid) and bicyclic amino moiety (2-aza-bicyclo[2.2.1]heptane) with a nitrile substituent. We performed molecular modelling predictions and compared the predicted DPP-4 inhibition activity of these compounds. We looked particularly at the presence of spontaneous intramolecular cyclisation previously described for the acylated cyanopyrrolidine derivatives [27]. This intramolecular cyclisation leads to the formation of substituted diketopiperazines with no affinity to DPP-4 (Figure 1).

**Figure 2:**
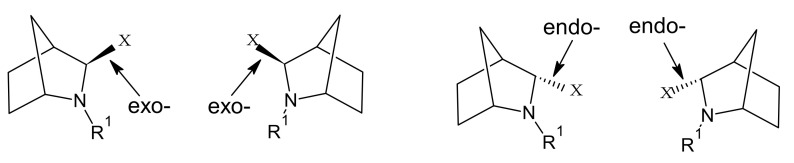
Possible stereoisomers of 3-substituted 2-aza-bicyclo[2.2.1]heptanes.

To avoid these negative consequences, we had an idea to modify the cyanopyrrolidine moiety by adding the second aliphatic cycle to make this part less flexible. The same unsymmetric ring system described in [28] for ledipasvir demonstrated beneficial SAR properties compared to pyrrolidine-based analogues. We also decided to prefer beta-amino acid (the same as the one in sita- and evogliptin) instead of alpha derivatives because of the enlargement of the aliphatic chain that connects cyanopyrrolidine moiety with the amino group that is crucial in protein-ligand interaction leads to energetically unfavourable cyclisation reaction profiles. The presence of chiral centre in 3-position of 2-aza-bicyclo[2.2.1]heptane results in two isomeric structures of *exo*- and *endo*-configuration (Figure 2).

**Figure 3:**
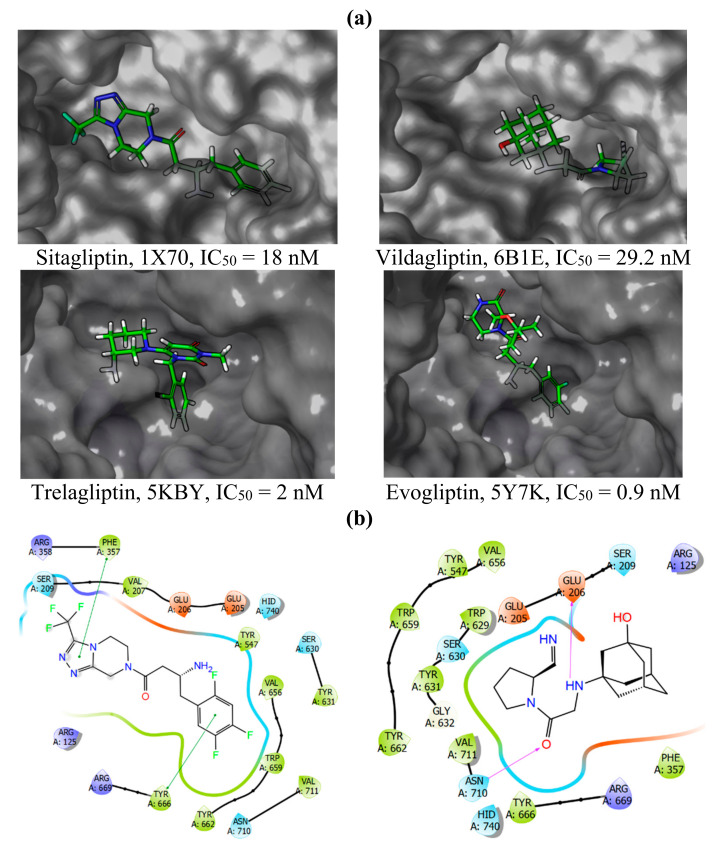

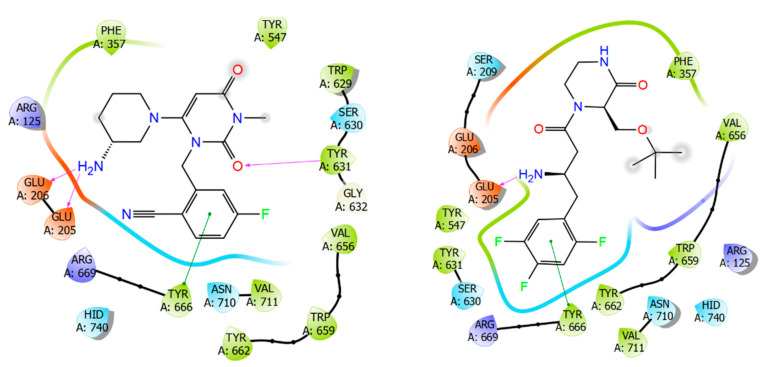
Binding parameters of most potent reference compounds. (**a**)–inhibitors in the DPP-4 binding cavity, (**b**)–ligand interactions diagram, showing the essential interacting residues. [Colour Figure 4. demonstrate that compound **12a** has interaction patterns similar to sitagliptin and vildagliptin. This fact is also complemented by scoring function values (Table 2). Although **12a** has GlideScore and Emodel values within the same range as for reference structures, its ∆Gbind value is preferred, indicating that the protein-ligand complex of DPP-4 with **12a** is energetically more favourable. Moreover, the predicted parameter of potential cardiotoxicity (QPlogHERG, blocking of HERG K+ ion channels) for **12a** is the safest. Sitagliptin has the highest risk of potential cardiotoxicity (the reference value less than -5 may result in high cardiotoxicity). Some reviews and clinical trials showed conflicting findings of this effect [30,31,32]. Predicted cardiotoxicity of **12a** is comparable to vildagliptin, which does not cause side effects associated with heart failure [33]; **12a** is also predicted to have a 1.5-fold reduced cardiotoxicity than sitagliptin. Thus, the new compound **12a** unifies the binding parameters of two known active DPP-4 inhibitors, and at the same time, is predicted to be less toxic. As can be seen in Figure 4, non-active compound **12b** binds in reversed, incorrect mode. This fact is linked with steric incompatibility with the active cleft of DPP-4. The same applies to compounds **12c,d**.

Next, we analysed protein-ligand binding modes and spatial orientation of reference compounds in terms of functional groups needed for correct ligand-protein interaction (Figure 3a,b). Amino acids in the active site of DPP-4 form different subsites (S2, S1, S’1, and S’2) that are usually occupied by different residues of the substrate molecule. The S1 pocket is formed predominantly by S630, D708, H740 (a catalytic triad), Y631, V656, W659, Y662, Y666, V711. Meanwhile, the S’2 pocket consists of R125, E205, E206, S209, F357. Critical residues of S’1 and S’2 binding pockets are Y547 and W629, respectively. Sitagliptin (S) and vildagliptin (V), the two earliest DPP-4 inhibitors approved, occupy the active centre differently (Figure 3). Due to the structural dissimilarity, they have differences in DPP-4 binding site filling efficiency (S > V), ability to form pi-stacking interaction with aromatic groups (S > V), hydrophobic contacts intensity (S > V), and hydrogen bonding intensity (S < V).

Our new compound **12a** has shown the following protein-ligand interaction profile: Residues in S’1 and S’2 binding pockets are involved in hydrophobic interactions. Trifluorophenyl aromatic ring forms significant hydrophobic site-specific interaction in S1 and S2 pockets using crucial π-π stacking interactions with tyrosine sidechains and hydrogen bonds with E205/E206. Trifluorophenyl ligand moiety partially occupies the S1 pocket, thus forming π-π stacking with Y666/Y662 and H740. Additional aliphatic cycle allows **12a** to form hydrophobic interactions with residues in the S2 extensive region.

Also, **12a** contains the cyanopyrrolidine moiety and, thus, mimics the penultimate proline of DPP-4 substrates; its nitrile moiety is targeted to occupy the S’1 binding pocket. This interaction mediates the formation of a hydrogen bond with Arg669 in the S’1 pocket. Here, the nitrile group is a non-covalent interactant [29] and an important part of the site-specific interaction machinery (as in vildagliptin, Figure 3). 

**Figure 4:**
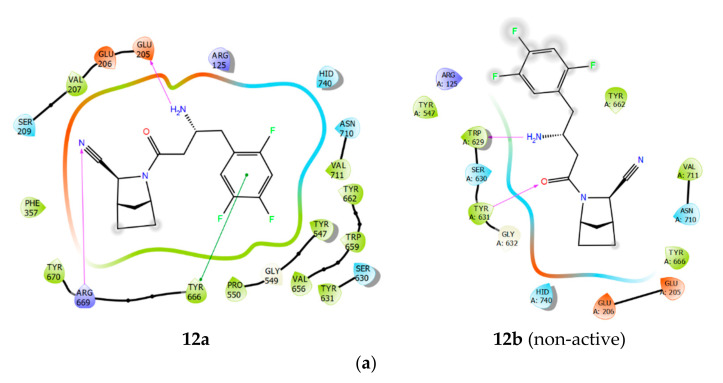

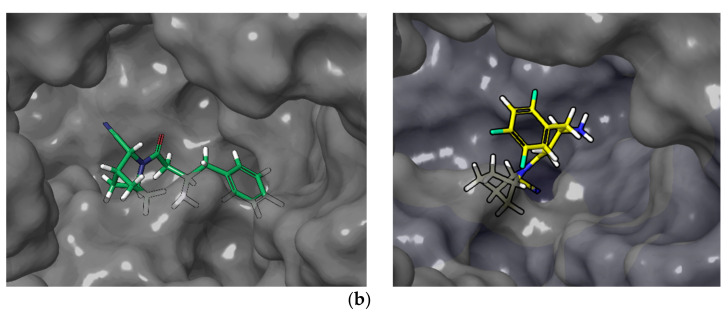
Ligand interaction diagram of **12a,b** in the active site of DPP-4 (**a**) and ligand positioning in DPP-4 binding cavity (**b**).

Additionally, this fact is confirmed with MM-GBSA free energy calculations (Table 2), compounds **12b–d** showed a significant increase in free Gibbs (ΔG) energy, which would result in the unfavourability of ligand-protein complex with DPP-4 (also confirmed by docking results). We observed a loss in site-specificity that resulted in the absence of some protein-ligand interactions that are typical for reference compounds (see Figure 3).

### 2.2. Chemical Synthesis

**Figure 5:**
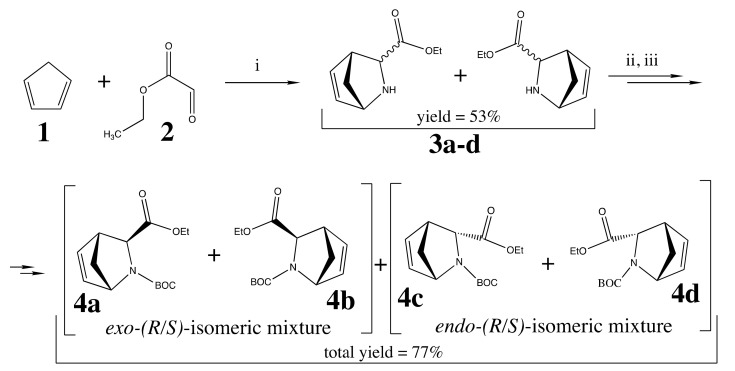
Synthesis of intermediates **4a,b** and **4c,d**. Reagents and conditions: (i) sat. NH_4_Cl for 12h at rt; (ii) Boc_2_O, THF overnight at rt (iii) separation by column chromatography, gradient: petroleum ether (PE) → 30% EtOAc in PE.

**Figure 6:**
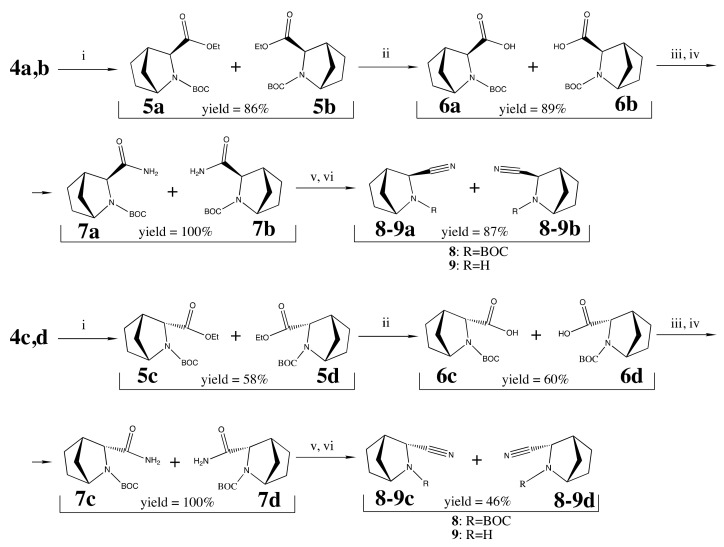
Synthesis of *exo*- and *endo*-intermediates **9a,b** and **9c,d**. Reagents and conditions: (i) H_2_, 10% Pd/C, EtOAc for 1.5h at t = 55–60 °C and *p* = 45 PSI; (ii) LiOH, MeOH, H_2_O for 3h at t = 40–50 °C; (iii) EtOCOCl, THF, for 10 min at t = −20 °C; (iv) NH_3_⋅H_2_O, Et_3_N, THF, for 1h at t = −20°C; (v) TFAA, THF for 3 h at t = 0–4 °C; (vi) p-TSA, CH_3_CN overnight at rt; acid residue removal with NH_3_ in DCM.

The general synthetic route for preparing the key intermediates, *R/S-Exo*- and *R/S-endo*-3-azabicyclo[2.2.1]heptane-2-carbonitriles, is shown in Figure 5 and Figure 6. Initially, starting from cyclopenta-1,3-diene (**1**), ethyl oxoacetate (**2**), and ammonia chloride, a mixture of four different stereoisomeric compounds (**3a–d**) were obtained [34,35,36]. Subsequent treatment of the mixture with Boc2O followed by column chromatography procedure afforded the separation of *exo*- and *endo*-isomeric pairs **4a,b** and **4c,d** (Figure 5) [37].

Both mixtures **4a,b** and **4c,d** were subjected to multistep synthetic route including common procedures of hydrogenation, saponification, amide to nitrile conversion receiving the intermediates **5–8a,b** and **5–8c,d** [37,38,39] followed by final Boc-deprotection with p-toluenesulfonic acid to afford carbonitriles **9a,b** (*exo*-) and **9c,d** (*endo*-) (Figure 6), previously mentioned in [40].

**Figure 7:**
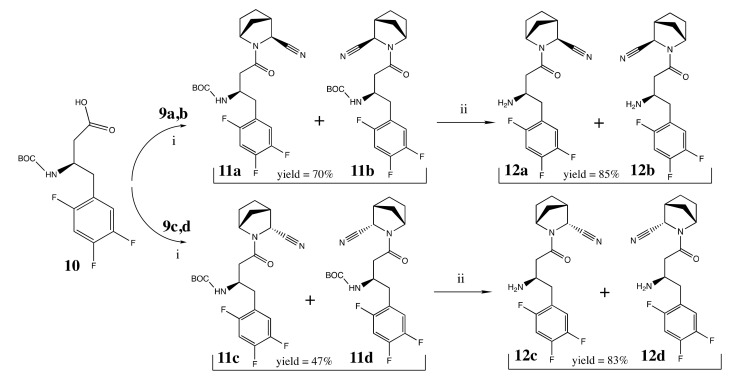
Synthesis of inhibitors **12a,b** and **12c,d**. Reagents and conditions: (i) BOP, DIPEA, DCM overnight at rt; (ii) p-TSA, CH_3_CN overnight at rt, products were obtained as *p*-TSA salts.

Both mixtures, **9a,b** and **9c,d**, were coupled with Boc-protected (*R*)-3-amino-4-(2,4,5-trifluorophenyl)butanoic acid **10** under standard conditions to yield after deprotection the corresponding final compounds **12a,b** and **12c,d** (Figure 7).

Here we also provide a scheme for the stereoselective synthesis of *S*- and *R-exo*-2-azabicyclo[2.2.1]heptane-carbonitriles starting from *S* and *R*-1-phenylethylamine, respectively. This synthetic route appears to be more promising than the necessity of isolating each resulting diastereomer from the mixture on the final step.

**Figure 8:**
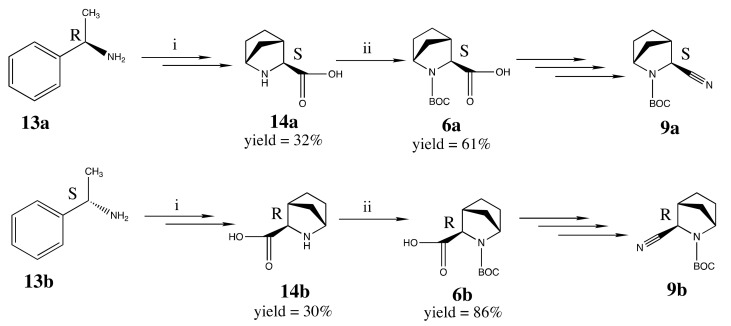
Synthesis of individual *S-exo*- and *R-exo*-stereoisomeric intermediates **6a** and **6b**. Reagents and conditions: (i) OHC-COOEt; cyclopentadiene, CF_3_COOH/ H_2_O, DMF at rt; (ii) Boc2O, EtOH for 24 h at rt.

For the synthesis of pure 3*S*- and 3*R*-*exo*-2-azabicyclo[2.2.1]heptane-carbonitriles **9a** and **9b**, we used the intermediates **6a** and **6b**, correspondingly. The 3*S*-acid **6a** was prepared from the precursor **14a** as described in the literature [38,41,42,43,44,45,46] and shown in Figure 8. The use of (*R*)-1-phenylethylamine **13a** followed by two-step hydrogenation and saponification afforded the only isomer **14a** with the desired *S*-configuration of carbon in 3-position. Subsequent Boc-protection step afforded to the pure isomeric intermediate **6a** [42]. The intermediate of 3*R*-configuration, **6b**, was prepared from **13b** by the analogy with **6a** [47]. The use of another enantiomeric starting reagent (S)-1-phenylethylamine **13b** afforded the pure compound **6b** of 3*R*-*Exo*-configuration [45]. The following steps to prepare **9a** and **9b** were carried out by the analogy with the scheme shown in Figure 7.

The obtained individual isomeric intermediates *S*- and *R*-*exo*-2-azabicyclo[2.2.1]heptane-carbonitriles **9a** and **9b** were used to synthesise the corresponding individual compounds **12a** and **12b**.

### 2.3. Structure and Purity Confirmation

The structures of key target final compounds **12** were unambiguously confirmed by 1H, 13C{1H}, 13C apt, COSY, HSQC, and NOESY NMR spectroscopy and liquid chromatography-mass spectrometry (LC/MS) methods. For structure confirmation of their precursors, **11**, 1H NMR spectroscopy was applied, and in some cases, if necessary, 13C NMR spectra were also registered. In each case from compounds **11** and **12**, chromatograms obtained by LC/MS analysis revealed the only molecular ion peak of 100% purity (see Supplementary file S1_NMR. Supplementary file S2_mass_spectra).

The exact assignment of individual proton signals in 1H NMR spectra of the final compounds **12** and their precursors **11** seemed not to be an easy task due to the overlapping and complex multiplicity of most signals. The doubling of some proton signals further complicated the task—however, total intensities of the same signals precisely correlated with each other.

**Figure 9:**
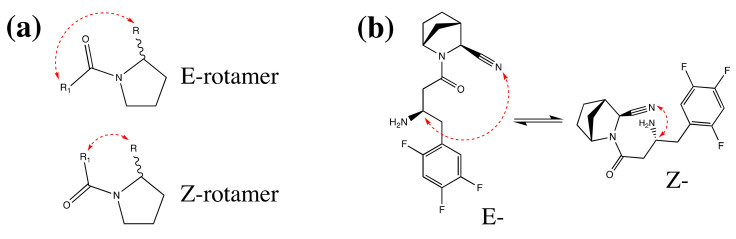
(**a**) *E/Z* rotamers in case of N-acylated 2-substituted pyrrolidines; (**b)** *E/Z*-rotameric forms of compound **12a**.

Signal doubling of the identical type protons is in good accordance with the existence of equilibrium *E/Z*-rotameric forms described in the literature. It is well known (see, for example, [48]) that the introduction of the N-acyl function in any 2-substituted pyrrolidines always leads to the formation of *E/Z*-rotameric forms (Figure 9a). The main reason for the stabilisation of rotamers is the hindered rotation around the N(1) -CO amide bond, and an additional reason is a steric factor [proximity of the substituent at C(2) in pyrrolidine]. In most cases, the existence of equilibrium forms can be seen in 1H NMR spectra by doubling the corresponding proton signals (see, for example, [49]).

All the synthesised inhibitors belonging to the class of N-acyl-2-substituted pyrrolidines should exist in solutions in *E/Z*-rotameric forms, as it has been shown for **12a**, as an example (Figure 9b).

**Figure 10:**
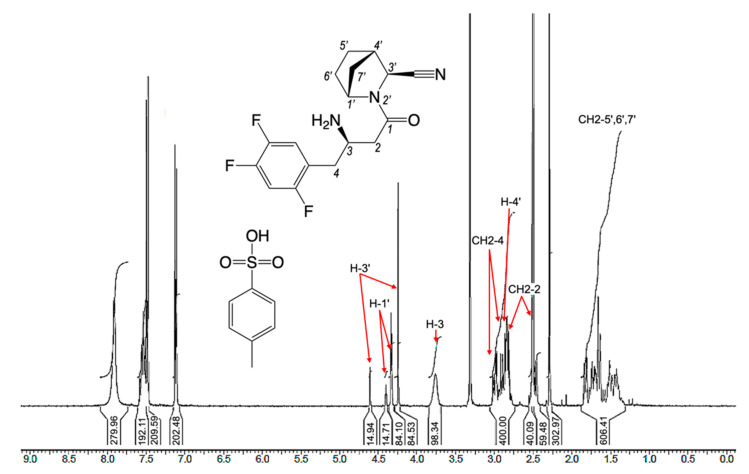
1H NMR spectrum of compound **12a** at room temperature.

Indeed, the presence of rotamers of all the synthesised compounds **11a–d** and **12a–d** was confirmed by registration of 1H NMR spectra with doubling proton signals at room temperature (see Section 3.5 and Supplementary Materials) as it is shown for **12a** as an example (Figure 10).

The presence of rotameric forms was also evident in the 13C{1H} NMR spectra (see Section 3.5 and Supplementary file S1_NMR). The exact proton and carbon nuclei signals assignment was performed using 13C atp, COSY, HSQC, HMBC NMR spectroscopy (see Supplementary file S1_NMR).

**Figure 11:**
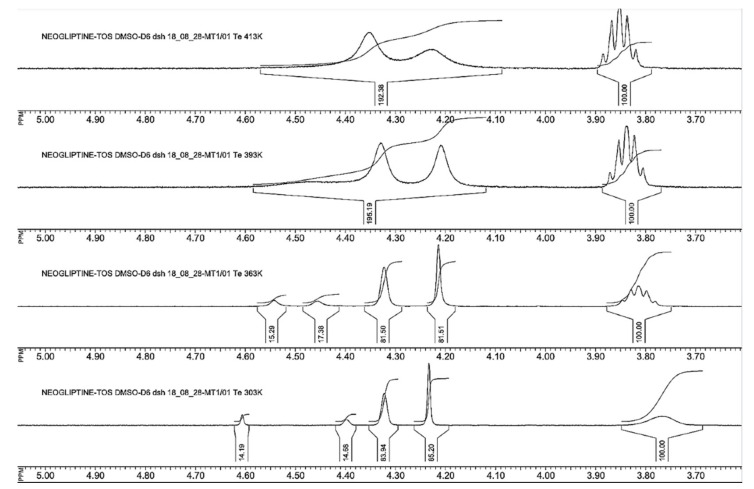
Fragments of 1H NMR spectra with H-1′, H-3′, and H-3 proton signals of compound **12a** in the temperature range 30–140 °C.

In order to prove the existence of *E/Z*-rotameric forms in all the synthesised compounds, three representatives, **12a**, **12b,** and their mixture **12a,b**, were chosen. Registration of 1H NMR spectra of these compounds in the temperature range 30–140 °C unambiguously confirmed the *E/Z*-rotamerism. For example, fragments of 1H NMR spectra of **12a** in a dimethyl sulfoxide (DMSO)-d6 solution at different temperatures are shown in Figure 11. Each methine proton signal, H-1′ and H-3′ (numeration in Figure 10), of the azanorbornane fragment was registered in the temperature range 30–90 °C as two broadened singlets in the ratio of ~85:15. The energy barrier of free rotation around the amide bond is large enough, and it is possible to overcome it only at a temperature of more than 140 °C. At a temperature of nearly 140 °C, a partial coalescence of the abovementioned signals was observed.

Coalescence of H-1′ and H-3′ proton signals in 1H NMR spectrum of **12b** was also found at a temperature above 140 °C (see Supplementary materials). The ratio of *E/Z*-rotameric forms of 85:15 in DMSO-d6 solution at room temperature calculated based on 1H NMR data was almost the same for all the synthesised compounds, **11** and **12**. Therefore, the data set of results obtained by NMR and LC/MS methods unambiguously confirmed that the final inhibitors **12a** and **12b** used for in vitro activity assay were chemically and stereochemically pure. The compounds **12a,b** and **12c,d** were diastereomeric mixtures and did not contain any side components.

### 2.4. Inhibitory Activity Evaluation

The DDP-4 enzyme inhibitory activities of the synthesised compounds were assessed using protocols similar to those described in the literature [50]. For IC50 values determination, inhibitory assays were carried out using recombinant DPP-4 enzyme D4943, chromogenic substrate Gly-Pro-pNA and buffer system (50 mM Tris-HCl, 50 mM NaCl, 0.01% Triton, pH = 7.6). After short incubation (37 °C for 30 min), the absorbance at 405 nm was measured. The procedure was initially optimised using reference compounds. Each inhibitor was analysed in the dilution range from 10^−4^ to 10^−11^ M. Certified samples of commercial drugs-inhibitors of DPP-4 (vilda-, sita-, alo- and linagliptin) with known IC50 were used as reference compounds. It was necessary to ensure that IC50 values estimated during the experiments fit into the ranges of values known for them from the literature and were reliably reproduced. Only after that precaution, synthesised compounds were tested. Correspondingly, the inhibition activity analysis of related enzymes (DPP-8 and DPP-9) was performed by fluorescent method with DPP-8 assay Kit and DPP-9 assay Kit. We analysed every compound in the dilution range 10^−2^ to 10^−8^ M (Table 3).

## 3. Materials and Methods

### 3.1. Molecular Modelling

The docking procedure was performed using the Schrödinger Glide module in standard precision mode. The docking grid was calculated according to native ligands dimensions using available PDB models (1X70 and 6B1E). In order to perform ligand docking, protein structures were superimposed, and coordinates of the docking grid were tied to the ligand centroid. The docking area was limited per reference ligand size, with 7 Å as a buffer zone. Grid spacing was set 0.375 Å, VdW radii cut-off 0.8 Å. Several optional constraints were added: nitrile group orientation (reference—vildagliptin), hydrophobic attraction—halogen-substituted moiety (sitagliptin). Docking solutions generation was performed using the Glide module of Schrödinger Suite (version 2020-3) in standard precision mode with 0.8 Å VdW radius and with previously mentioned optional constraints. Docking protocol was validated by redocking of reference compounds. The geometry of the best-fitting ligand was shown on ligand interaction diagrams. For each inhibitor, 45 docking solutions were generated, the best 15 were used for binding mode analysis. GlideScore and EModel values-controlled target affinity. Optimal binding poses were selected per cluster RMSD less than 1.5 Å. Binding pose and calculated parameters of reference ligand were taken as a control. Free Gibbs energy (∆G) was calculated using the MM-GBSA method, implemented in Schrodinger Suite v.2020-3, module Prime. All results were processed using Maestro molecular modelling interface (Schrodinger Suite v.2020-3). All protein-ligand complexes were prepared and refined using Schrodinger Protein Prepwizard. This procedure was essential to fix missing amino acid sidechains, incorrect bond orders, and correct protonation states. Optimal binding poses were selected by cluster RMSD less than 1.5 Å. Binding parameters of the reference ligand were selected as a control. To prove the structural novelty of identified hits, we used the Tanimoto coefficient that shows similarity scores between library compounds and already known drugs. A score below 0.5 is a good sign of low similarity. We used nine marketed gliptins (sita-, vilda-, alo-, lina-, saxa-, teneli-, trela-, evo- and omarigliptin) as reference compounds. The search for hits (top-scoring compounds with residues involved in the binding site of the enzyme with an increase in field regions) [51] was carried out in a library of chemical structures with a high level of similarity (Tanimoto coefficient more than 0.8), built on 3- azabicyclo[2.2.1]heptene-2-carbonitrile scaffolds. Potential toxicity evaluation of ligands was carried out using the QikProp module, using 2D/3D-QSAR descriptor combinations analysis.

#### 3.1.1. Molecular Modelling Hardware

- All calculations were processed at Sechenov First Moscow State Medical University, using the following hardware:
- Fujitsu RX4770M3 CPU-server. Intel(R) Xeon(R) CPU E7-8867 v4 @ 2.40GHz 18C/36T x4, 1024Gb RAM.
- Fujitsu RX2540M4 GPU-server. Intel(R) Xeon(R) Gold 6138 CPU @ 2.00GHz 20C/80T x2, 1024Gb RAM, 2xTesla P100 16Gb.
- Fujitsu Celsius R940 GPU-server. Intel(R) Xeon(R) CPU E5-2690 v4 @ 2.60GHz 14C/28T x2, 256Gb RAM, 2xGTX 1080Ti 11Gb.

#### 3.1.2. Used Software

- Maestro. Schrödinger Release 2020-3: Maestro, Schrödinger, LLC, New York, NY, USA, 2020.
- LigPrep. Schrödinger Release 2020-3: LigPrep, Schrödinger, LLC, New York, NY, USA, 2020.
- Prime. Schrödinger Release 2020-3: Prime, Schrödinger, LLC, New York, NY, USA, 2020.
- Glide module. Schrödinger Release 2020-3: Glide, Schrödinger, LLC, New York, NY, USA, 2020.

### 3.2. Chemical Synthesis

All starting reagents were bought from reliable commercial vendors, mostly Sigma-Aldrich, Merck, and Acros, and used without further purification. Intermediates and final compounds were isolated using column chromatography on silica gel. Compounds were only used for biological evaluation if the purity was ≥95%.

### 3.3. LC/MS, NMR, and Elemental Analysis

Liquid chromatography-mass spectrometry (LC/MS), NMR spectroscopy, and elemental analysis methods were applied to confirm the structure and purity of all synthesised compounds.

LC/MS analysis was performed on an 1100 LC (Agilent Technologies) with ELSD, UV (DAD 200–400 nm), and mass detection (1100 LCMSD, Agilent Technologies, APCI, and ES positive ionization). The most used column was the Onix C18 50 × 4.6 mm; eluent 1–0.1% TFA in water; eluent 2–0.1% TFA in acetonitrile, gradient—eluent 1–2.9 min, eluent 2–0.2 min, eluent 1—rinsing, flow rate—3.75 mL/min.

The structures of key target final compounds were unambiguously confirmed by 1H, 13C{1H},13C apt, COSY, HSQC, and NOESY NMR spectroscopy. For structure confirmation of intermediates, 1H NMR spectroscopy was applied, and in some cases, if it was necessary, 13C{1H} NMR spectra were also registered. NMR spectra were registered on spectrometers Bruker DRX 400 (400.13 MHz for protons, 100.61 MHz for carbons, 376.50 MHz for fluorine), Bruker Avance II+ 600 (600.11 MHz for protons, 150.93 MHz for carbons), Avance IIIHD 500 (500.13 MHz for protons, 125.78Mhz for carbons), and spectrometer Bruker Avance III 400 UltraShield Plus (400 MHz). CDCl3 and DMSO-d6 were used as solvents.

Elemental analysis was performed on Vario MICRO cube CHNS analyser (Elementar Analysensysteme GmbH, Hanau, Germany).

### 3.4. Inhibitory Activity Evaluation

For the inhibitory assays, we used the substrate Gly-Pro-p-nitroanilide (H-Gly-Pro-pNA HCl, G0513) and recombinant dipeptidyl peptidase-4 enzyme (D4943) that were purchased from Sigma-Aldrich (St. Louis, MO, USA). Samples containing DPP-4 (0.0015 U/well) and varying concentrations of test compounds were incubated with the chromogenic peptide substrate, G0513 (90 μg/well) in the total volume of 100 µL buffer system (50 mM Tris-HCl, 50 mM NaCl, 0.01% Triton, pH = 7.6). The mixtures were incubated at 37 °C for 30 min, and the absorbance at 405 nm was measured using the microplate reader ChemWell (Awareness Technology Inc., Palm City, FL, USA). DPP-4 inhibitory activities for each test compound were calculated. IC50 values were obtained using GraphPad Prism 8 software. Inhibition activity analysis of related enzymes (DPP-8 and DPP-9) was performed using “Fluorogenic DPP-8 Assay Kit” (BPS Bioscience) and “Fluorogenic DPP-9 Assay Kit” (BPS Bioscience). The dilution range for every compound was in the 10^−2^ to 10^−8^ M.

### 3.5. Experimental Section

**2-*tert*-Butyl 3-ethyl-2-azabicyclo[2.2.1]hept-5-ene-2,3-dicarboxylate (3a–d).**

The synthesis was made according to the procedure described in [34,35,36]. To a mixture of a saturated solution of ammonium chloride (39.3 g) and a toluene solution of ethyl glyoxylate (50%, 150 g) freshly prepared cyclopentadiene (64.7 g). The reaction mixture was stirred for 12 h at room temperature, then extracted with a mixture of methyl tert-butyl ether (MTBE) and petroleum ether (PE) in a 1:3 ratio. The aqueous layer was alkalised with NaOH aq. solution (50%) up to pH 8.0-9.0, extracted with MTBE and dried over anhydrous sodium sulphate. After solvent evaporation, the mixture of **3a–d** was obtained as a yellow oil (53%, 67 g) and used without any purification.

**2-*tert*-Butyl 3-ethyl (3*S,R*)-*exo*-2-azabicyclo[2.2.1]hept-5-ene-2,3-dicarboxylate (4a,b) and 2-*tert*-butyl 3-ethyl (3*R,S*)-*endo*-2-azabicyclo[2.2.1]hept-5-ene-2,3-dicarboxylate (4c,d).**

The synthesis was made according to the procedure described in [37]. To a solution of **3a–d** (38 g) in THF (200 mL), a solution of Boc-anhydride (55 g) was added dropwise in THF (200 mL) while cooling the reaction mixture with ice. The mixture was kept overnight under stirring at room temperature, then the solvent was evaporated, and the residue was dissolved in PE: EtOAc (in 1: 1 ratio) and washed with brine. The organic layer was dried over anhydrous sodium sulphate. The obtained mixture of *exo*- and *endo*-isomers was separated using column chromatography. Eluent: PE -> PE: EtOAc 30%. The separation afforded to pure *exo*-isomers **4a,b** (17 g) and *endo*-isomers **4c,d** (20 g). The total yield was 77%. 1H NMR (400 MHz, CDCl3), 𝛿 (ppm) for **4a,b**: 6.49 (m, 1H), 6.37 (1 m, 1H), 4.79, 4.66 (2 m, 1H, rotameric forms), 4.23–4.18 (m, 2H), 3.48, 3.39 (2 m, 1H, rotameric forms), 3.27 (m, 1H), 1.98–1.96 (m, 1H), 1.54–1.37 (m, 9H+1H, overlapped signals of rotameric forms), 1.32–1.25 (m, 3H). 1H NMR (400 MHz, CDCl3), 𝛿 (ppm) for **4c,d**: 6.54, 6.51 (2 m, 1H, rotameric forms), 6.09 (m, 1H), 4.86, 4.75 (2 m, 1H, rotameric forms), 4.32, 4.26 (2 m, 1H, rotameric forms), 4.15–4.09 (m, 2H), 3.47 (m, 1H), 1.67–1.65 (2 m, 2H), 1.46–1.39 (m, 9H), 1.26–1.22 (m, 3H).

**2-*tert*-Butyl 3-ethyl (3*S,R*)-*exo*-2-azabicyclo[2.2.1]heptane-2,3-dicarboxylate(5a,b)**

The synthesis was made according to the procedure described in [37]. A mixture of **4a,b** (17 g), 10% Pd/C (0.8 g), and EtOH was hydrogenated for 1.5 h at 55–60 °C and under a pressure of 45 PSI. The mixture was filtered through celite and evaporated to give the product **5a,b** (14.8 g, 86%) as a yellow oil. 1H NMR (400 MHz, CDCl3), 𝛿 (ppm): 4.31–4.11 (m overlapped, 3H), 3.78–3.64 (m, 1H), 2.63 (m, 1H), 1.87–1.45 (m overlapped, 6H), 1.47, 1.42 (2 s, 9H, rotameric forms), 1.24 (m overlapped, 3H, rotameric forms). LC-MS APCI: *m/z* 214 [M − t-Bu + H]+, 170 [M − Boc + H]+.

**2-*tert*-Butyl 3-ethyl (3*S,R*)-*endo*-2-azabicyclo[2.2.1]heptane-2,3-dicarboxylate(5c,d)**

The synthesis was made according to the procedure described in [37,38]. A mixture **5c,d** (10 g, 58%) was prepared from **4c,d** (17 g) by the analogy with **5a,b.** 1H NMR (400 MHz, CDCl3), 𝛿 (ppm): 4.35 (m, 1H), 4.24–4.10 (m, 3H), 2.74 (m, 1H), 2.02–1.60 (m overlapped, 6H), 1.45, 1.42 (2 s, 9H, rotameric forms), 1.24 (m overlapped, 3H, rotameric forms). LC-MS APCI: *m/z* 214 [M − t-Bu + H]+, 170 [M − Boc + H]+.

**(3*S,R*)-*exo*-2-(*tert*-Butoxycarbonyl)-2-azabicyclo[2.2.1]heptane-3-carboxylic acid (6a,b)**

The synthesis was made according to the procedure described in [37,39]. A mixture of ester **5a,b** (14.8 g), and LiOH (monohydrate, 6.58 g) in a water-methanol solution was stirred overnight at room temperature. TLC monitoring showed SM remained. Another 1.5 equivalents of LiOH were added, and the mixture was kept under stirring at 40–50 °C for 2 h. Then methanol was evaporated, the mixture was diluted with water, extracted with EtOAc, then the aqueous layer was acidified with citric acid to pH 4.0 and extracted with DCM. After drying with sodium sulphate and removing the solvent, the desired product **6a,b** (11.9 g, 89%) was obtained. 1H NMR (400 MHz, CDCl3), 𝛿 (ppm): 4.34, 4.13 (2 m, 1H, rotameric forms), 3.83, 3.73 (2 m, 1H, rotameric forms), 2.92, 2.73 (2 m, 1H, rotameric forms), 2.07–1.61 (m overlapped, 6H), 1.47, 1.42 (2 s, 9H, rotameric forms). LC-MS APCI: *m/z* 186 [M − t-Bu + H]+, 142 [M − Boc + H]+.

**(3*S*)-*exo*-2-(*tert*-Butoxycarbonyl)-2-azabicyclo[2.2.1]heptane-3-carboxylic acid (6a)**

The synthesis was made according to the procedure described in [42,46]. To EtOH (15 mL) solution of compound **14a** (0.9 g, 6.4 mmol) BOC2O (1.6 g, 7.4 mmol) was added. The mixture was stirred at RT for 24h and evaporated. Water (15 mL) was added to the residue. The product was extracted with EtOAc; then, the aqueous layer was acidified with citric acid to pH 4.0 and extracted with DCM. After drying with sodium sulphate and removing the solvent, the desired product **6a** (1 g, 61%) was obtained. 1H NMR and LC-MS are the same as described in **6a,b** synthesis.

**(3*R*)-*exo*-2-(*tert*-Butoxycarbonyl)-2-azabicyclo[2.2.1]heptane-3-carboxylic acid (6b)**

The synthesis was made according to the procedure described in [46,47]. Compound **6b** (0.77 g, 86%) was prepared from **14b** (0.8 g) by the analogy with **6a**. 1H NMR spectra and LC-MS for **6b** are the same as described in **6a,b** synthesis.

**(3*S,R*)-*endo*-2-(*tert*-Butoxycarbonyl)-2-azabicyclo[2.2.1]heptane-3-carboxylic acid (6c,d)**

The synthesis was made according to the procedure described in [37,38,39]. A mixture **6c,d** (4.9 g, 60%) was prepared from **5c,d** (9 g) by the analogy with **6a,b.** 1H NMR (400 MHz, CDCl3), 𝛿 (ppm): 4.36 (m, 1H), 4.23 (m, 1H), 2.84 (1 m, 1H, rotameric forms), 1.82–1.41 (m overlapped, 15H). LC-MS APCI: *m/z* 141 [M − Boc + H]+.

***tert*** **-Butyl (3*S,R*)-*exo*-3-carbamoyl-2-azabicyclo[2.2.1]heptane-2-carboxylate (7a,b)**

The synthesis was made according to the procedure described in [39,45]. TEA (6.54 mL, 4.75 g) was added to a solution of acid **6a,b** (10.3 g) in anhydrous THF under argon atmosphere at −20 °C. Then ethyl chloroformate (5.10 g) was added dropwise over 10 min. The mixture was kept undercooling for 40 min, and aqueous ammonia (8.36 g) was added dropwise while cooling. After one hour, THF was evaporated, the residue was treated with a citric acid solution to pH 4.0 and extracted with ethyl acetate. The combined organic solution was washed with aq. NaHCO3, dried over sodium sulphate and evaporated to give the product **7a,b** (10 g, quantitative yield) as a colourless crystalline residue. 1H NMR (400 MHz, CDCl3), 𝛿 (ppm): 6.85, 6.22 (2 broad signals, 1H, rotameric forms), 5.90, 5.69 (2 broad signals, 1H, rotameric forms), 4.23, 4.09 (2 m, 1H, rotameric forms), 3.76, 3.65 (2 m, 1H rotameric forms), 2.90, 2.79 (2 m, 1H, rotameric forms), 1.74–1.33 (m overlapped, 6H), 1.46, 1.44 (2 s, 9H, rotameric forms). LC-MS APCI: *m/z* 141 [M − Boc + H]+.

***tert*** **-Butyl (3*S,R*)-*endo*-3-carbamoyl-2-azabicyclo[2.2.1]heptane-2-carboxylate (7c,d)**

The synthesis was made according to the procedure described in [38,39]. A mixture **7c,d** (4 g, quantitative yield) was prepared from **6c,d** (4 g) by the analogy with **7a,b.** 1H NMR (400 MHz, CDCl3), 𝛿 (ppm): 6.25 (broad signal, 1H), 5.73 (broad signal, 1H), 4.47 (m, 1H), 4.13 (m, 1H), 2.90 (m, 1H), 2.04–1.51 (m overlapped, 6H), 1.47, 1.44 (2 s, 9H, rotameric forms). LC-MS APCI: *m/z* 140 [M − Boc]+.

***tert*** **-butyl (3*S,R*)-*exo*-3-cyano-2-azabicyclo[2.2.1]heptane-2-carboxylate (8a,b)**

Trifluoroacetic anhydride (14.4 g) was added dropwise to a suspension of amide **7a,b** (10.3 g) in anhydrous THF, at a temperature not higher than 4 °C, within 10 min. After the TLC, it became clear that the starting amide was still present, and another portion of trifluoroacetic anhydride (9 g) was added. The mixture was kept undercooling for 3 h, then ammonium hydrogen carbonate (45 g) was added portion-wise. The mixture was subjected to column chromatography. Eluent: a mixture of PE: EtOAc 4: 1. Boc-protected nitrile **8a,b** (8.3 g, 87%) was obtained as a yellow oil. 1H NMR (400 MHz, CDCl3), 𝛿 (ppm): 4.34, 4.20 (2 m, 1H, rotameric forms), 4.02, 3.91 (2 m, 1H, rotameric forms), 2.85 (m, 1H), 2.11–2.01 (m overlapped, 1H), 1.81–1.48 (m overlapped, 5H,) 1.48, 1.44 (2 s, 9H, rotameric forms). Note: on ^1^H NMR spectrum peaks of residual EtOAc are present. 13C{1H} NMR (100 MHz, DMSO-*d6*), 𝛿 (ppm): 153.93, 152.64, 118.09, 81.38, 80.99, 57.65, 56.48, 52.79, 52.50, 43.23, 42.6, 37.19, 36.54, 30.35, 30.15, 28.46, 28.39, 26.61, 26.44. LC-MS APCI: *m/z* 123 [M − Boc + H]+. Anal. calcd for **8a,b** (1:1 mixture of isomers) C_12_H_18_N_2_O_2_ * 0.4 C_4_H_8_O_2_ (EtOAc): C, 63.43; H, 8.30; N, 10.88. Found: C, 63.62; H, 8.16; N, 11.07%. Anal. calcd for **8a** C_12_H_18_N_2_O_2_: C, 64.84; H, 8.16; N, 12.60. Found: C, 64.95; H, 8.39; N, 12.77%. Anal. calcd for **8b** C_12_H_18_N_2_O_2_: C, 64.84; H, 8.16; N, 12.60. Found: C, 65.12; H, 8.25; N, 12.37%.

***tert*** **-butyl (3*S,R*)-*endo*-3-cyano-2-azabicyclo[2.2.1]heptane-2-carboxylate (8c,d)**

A mixture **8c,d** (1.5 g, 46%) was prepared from **6c,d** (3.5 g) by the analogy with **8a,b.** 1H NMR (400 MHz, CDCl3), 𝛿 (ppm): 4.38 (m, 1H), 4.26 (m, 1H), 2.81 (m, 1H), 2.02−1.53 (m overlapped, 6H), 1.47 (s, 9H). Note: on ^1^H NMR spectrum peaks of residual EtOAc are present. 13C{1H} NMR (100 MHz, DMSO-*d6*), 𝛿 (ppm): 153.40, 152.53, 118.18, 117.85, 81.35, 80.92, 57.96, 56.78, 52.37, 51.70, 41.81, 41.23, 37.79, 37.50, 30.22, 28.35, 24.04. LC-MS APCI: *m/z* 123 [M − Boc + H]+. Anal. calcd for **8c,d** (1:1 mixture of isomers) C_12_H_18_N_2_O_2_ * 0.1 C_4_H_8_O_2_ (EtOAc): C, 64.45; H, 8.20; N, 12.12. Found: C, 64.69; H, 8.32; N, 12.17%.

**(3*S,R*)-*exo*-2-azabicyclo[2.2.1]heptane-3-carbonitrile (9a,b)**

To a solution of BOC-protected nitrile **8a,b** (7 g) in acetonitrile (30 mL), p-TSA (12 g, two-fold excess) was added. The mixture was stirred overnight. Acetonitrile was evaporated, the residue was triturated with diethyl ether (3–4 treatments with decantation). The residue was dissolved in DCM and saturated with ammonia from a balloon. The precipitated ammonium salt of *p*-TSA was filtered. The filtrate was evaporated, and the residue was purified by column chromatography. Eluent: DCM after extraction of aqueous ammonia in the ratio of 1:10. The desired product **9a,b** (3.2 g, 83%) was obtained as a yellow oil. 1H NMR (400 MHz, CDCl3), 𝛿 (ppm): 5.38 (broad signal, 1H), 3.58 (m, 1H), 3.56 (m, 1H), 2.72 (m, 1H), 1.68 (m, 2H), 1.46–1.31 (m overlapped, 4H). Note: on ^1^H NMR spectrum peaks of residual N-tert-butylacetamide are present. 13C{1H} NMR (100 MHz, DMSO-*d6*), 𝛿 (ppm): 121.03, 55.99, 51.09, 42.44, 36.39, 32.64, 27.35. LC-MS APCI: *m/z* 123 [M + H]+. Anal. calcd for **9a,b** (1:1 mixture of isomers) C_7_H_10_N_2_ * 0.6 C_6_H_13_NO (N-tert-butylacetamide): C, 66.56; H, 9.38; N, 19.04. Found: C, 66.69; H, 9.42; N, 18.95%.

**(3*S*)-*exo*-2-azabicyclo[2.2.1]heptane-3-carbonitrile (9a)**

Compound **9a** was prepared from **6a** by the multistep synthetic route described for **9a,b**. 1H NMR spectra and LC/MS for **9a** are the same as described in **9a,b** synthesis. Anal. calcd for C_7_H_10_N_2_ * 0.1 C_6_H_13_NO (N-tert-butylacetamide): C, 68.28; H, 8.52; N, 22.00. Found: C, 68.58; H, 8.67; N, 22.00%.

**(3R)-*exo*-2-azabicyclo[2.2.1]heptane-3-carbonitrile (9b)**

Compound **9b** was prepared from **6b** by the multistep synthetic route described for **9a,b**. 1H NMR spectra and LC/MS for **9b** are the same as described in **9a,b** synthesis. Anal. calcd for C_7_H_10_N_2_ * 0.2 C_6_H_13_NO (N-tert-butylacetamide): C, 67.83; H, 8.75; N, 21.22. Found: C, 68.08; H, 8.87; N, 21.01%.

**(3*S,R*)-*endo*-2-azabicyclo[2.2.1]heptane-3-carbonitrile (9c,d)**

A mixture **9c,d** (0.4 g, 52%) was prepared from **7c,d** (1.4 g) by the analogy with **9a,b.** 1H NMR (400 MHz, CDCl3), 𝛿 (ppm): 5.52 (broad signal, 1H), 3.87 (m, 1H), 3.45 (m, 1H), 2.60 (m, 1H), 1.87–1.83 (m, 2H), 1.63–1.50 (m, 4H). Note: on ^1^H NMR spectrum peaks of residual N-tert-butylacetamide are present. 13C{1H} NMR (100 MHz, DMSO-*d6*), 𝛿 (ppm): 120.94, 56.67, 50.53, 40.46, 38.83, 31.57, 24.29. LC-MS APCI: *m/z* 123 [M + H]+. Anal. calcd for **9c,d** (1:1 mixture of isomers) C_7_H_10_N_2_ * 0.3 C_6_H_13_NO (N-tert-butylacetamide): C, 67.44; H, 8.94; N, 20.56. Found: C, 67.68; H, 8.94; N, 20.64%.

***tert*** **-Butyl {(3*R*)-4-[(3*S,R*)-3-cyano-*exo*-2-azabicyclo[2.2.1]heptan-2-yl]-1-(2,4,5-trifluorophenyl)-4-oxobutan-2-yl)}carbamate (11a,b)**

To a solution of acid **10** (0.19 g, 1 mmol) in DCM (20 mL) were added DIPEA (0.13 g, 1 mmol), BOP (0.44 g, 1 mmol), and amine **9a,b** (0.12 g, 1 mmol). The mixture was stirred at room temperature overnight and washed with 5% aqueous citric acid solution (3 × 10 mL) and 10% NaHCO3 (3 × 10 mL). The organic layer was dried over anh. sodium sulphate. The solvent was evaporated. The residue was dissolved in chloroform and purified by silica gel column chromatography (eluent 1–5% MeOH/CHCl3) to yield **11a,b** (0.3 g, 70%). 1H NMR (400 MHz, DMSO-*d6*), 𝛿 (ppm): 7.46 (m, 1H, Ph), 7.29 (m, 1H, Ph), 6.86, 6.80, 6.73, 6.39 (4 m, 1H, NH-Boc, 2 diastereomers in 2 rotameric forms), 4.68, 4.57, 4.41, 4.39, 4.34, 4.26, 4.24 (7 m, 2H, 2 diastereomers in 2 rotameric forms), 4.05 (m, 1H), 2.94–2.78 (m, 2H), 2.70–2.56, 2.46–2.33 (m, 3H, 2 diastereomers in 2 rotameric forms), 1.85–1.42 (m overlapped, 6H), 1.27, 1.24, 1.19 (3 m, 9H, Boc, 2 diastereomers in 2 rotameric forms). 13C{1H} NMR (100 MHz, DMSO-*d6*), 𝛿 (ppm): 168.61, 168.32, 168.16, 154.75–154.83, 149.06, 146.77, 144.39, 122.69–122.89, 119.15–119.41, 118.52, 118.12, 105.24–105.52, 77.70, 77.63, 57.88. 57.86, 56.07, 56.03, 52.13, 51.11, 50.98, 48.17, 47.44, 47.12, 42.74, 41.61, 41.59, 37.23, 37.20, 35.34, 33.17, 33.13, 30.49, 29.28, 28.11, 28.05, 27.76, 25.53, 25.51, 25.19. LC-MS APCI: *m/z* 382 [M − t-Bu + H]+, 338 [M − Boc + H]+. Anal. calcd for **11a,b** (1:1 mixture of isomers) C_22_H_26_N_3_O_3_F_3_: C, 60.40; H, 5.99; N, 9.61. Found: C, 60.61; H, 6.04; N, 9.61%.

***tert*** **-Butyl {(3*R*)-4-[(3*S*)-3-cyano-*exo*-2-azabicyclo[2.2.1]heptan-2-yl]-1-(2,4,5-trifluorophenyl)-4-oxobutan-2-yl)}carbamate (11a)**

Compound **11a** (0.29 g, 68%) was prepared from acid **10** (0.19 g, 1 mmol) and amine **9a** (0.12 g, 1 mmol) by the analogy with **11a,b.** 1H NMR (600 MHz, DMSO-*d6*), 𝛿 (ppm): 7.51–7.45 (m, 1H, Ph), 7.31 (m, 1H, Ph), 6.84, 6.77, 6.39 (3 m, 1H, NH-Boc, 2 rotameric forms), 4.58, 4.26 (2 m, 1H, CHCN, 2 rotameric forms), 4.39, 4.34 (2 m, 1H, 2 rotameric forms), 4.05 (m, 1H), 2.85–2.78 (m, 2H), 2.64-2.55 (m, 2H), 2.50–2.46 (m, 1H), 1.85-1.42 (m overlapped, 6H), 1.27, 1.20 (2 m, 9H, Boc, 2 rotameric forms). LC-MS AP-ES: *m/z* 382 [M − t-Bu + H]+, 338 [M − Boc + H]+. Anal. calcd for C_22_H_26_N_3_O_3_F_3_: C, 60.40; H, 5.99; N, 9.61. Found: C, 60.55; H, 6.11; N, 9.36%.

***tert*** **-Butyl {(3*R*)-4-[(3R)-3-cyano-*exo*-2-azabicyclo[2.2.1]heptan-2-yl]-1-(2,4,5-trifluorophenyl)-4-oxobutan-2-yl)}carbamate (11b)**

Compound **11b** (0.28 g, 65%) was prepared from acid **10** (0.19 g, 1 mmol) and amine **9b** (0.12 g, 1 mmol) by the analogy with **11a,b.** 1H NMR (500 MHz, DMSO-*d6*), 𝛿 (ppm): 7.52–7.43 (m, 1H, Ph), 7.30 (m, 1H, Ph), 6.86, 6.80, 6.42 (3 m, 1H, NH-Boc, 2 rotameric forms), 4.68, 4.42 (2 m, 1H, CHCN, 2 rotameric forms), 4.39, 4.25 (2 m, 1H, 2 rotameric forms), 4.03 (m, 1H), 2.95–2.83 (m, 2H), 2.69–2.55 (m, 2H), 2.40–2.34 (m, 1H), 1.86–1.42 (m overlapped, 6H), 1.28, 1.25, 1.19 (3 m, 9H, Boc, rotameric forms). LC-MS APCI: *m/z* 382 [M − t-Bu + H]+, 338 [M − Boc + H]+. Anal. calcd for C_22_H_26_N_3_O_3_F_3_: C, 60.40; H, 5.99; N, 9.61. Found: C, 60.65; H, 6.06; N, 9.73%.

***tert*** **-Butyl {(3*R*)-4-[(3*S,R*)-3-cyano-*endo*-2-azabicyclo[2.2.1]heptan-2-yl]-1-(2,4,5-trifluorophenyl)-4-oxobutan-2-yl)}carbamate (11c,d)**

A mixture **11c,d** (0.2 g, 47%) was prepared from acid **10** (0.19 g, 1 mmol) and amine **9c,d** (0.12 g, 1 mmol) by the analogy with **11a,b.** 1H NMR (600 MHz, DMSO-*d6*), 𝛿 (ppm): 7.42 (m, 1H, Ph), 7.29 (m, 1H, Ph), 6.71, 6.33 (2 m, 1H, NH-Boc, 2 diastereomers), 4.85, 4.62, 4.59, 4.46, 4.44, 4.41, 4.34 (7 m, 2H, 2 diastereomers in 2 rotameric forms), 4.02 (m, 1H), 2.96–2.84 (m, 2H), 2.66–2.54 (m, 2H), 2.50–2.43 (m, 1H), 1.75-1.57 (m overlapped, 6H), 1.29, 1.28, 1.22 (3 m, 9H, H-Boc, 2 diastereomers in 2 rotameric forms). 13C{1H} NMR (100 MHz, DMSO-*d6*), 𝛿 (ppm): 167.61, 167.55, 157.23–157.38, 154.85, 154.83, 154.16, 148.84–149.28, 146.52–146.95, 144.39–144.60, 122.64–122.98, 119.20-119.47, 118.04, 117.96, 105.27–105.79, 103.13, 77.73, 77.71, 63.02, 58.27, 58.16, 50.67, 50.61, 47.59, 47.47, 39.98, 39.95, 37.49, 37.35, 33.19, 33.03, 30.10, 29.98, 28.15, 28.12, 27.79, 23.62, 18.47, 15.39. LC-MS APCI: *m/z* 382 [M − t-Bu + H]+, 338 [M − Boc + H]+. Anal. calcd for **11c,d** (1:1 mixture of isomers) C_22_H_26_N_3_O_3_F_3_: C, 60.40; H, 5.99; N, 9.61. Found: C, 60.24; H, 6.12; N, 9.73%.

***(*** **3*S,R)*-2-[(3*R*)-3-Amino-4-(2,4,5-trifluorophenyl)butanoyl]-*exo*-2-azabicyclo[2.2.1]hepnane-3-carbonitrile *p*-toluenesulpfonate (12a,b)**

Compound **11a,b** (0.30 g, 0.69 mmol) was dissolved in acetonitrile (20 mL) and p-TSA (2 eq.) was added. The mixture was stirred at room temperature overnight. The precipitate was filtered off, washed with diethyl ether and dried in vacuo to give **12a,b** as p-TSA-salt (0.3 g, 85%). 1H NMR (500 MHz, DMSO-*d6*): 𝛿 7.93 (br.s, 3H, NH3+), 7.56–7.48 (m + d overlapped, 4H, *J*d = 4 Hz), 7.12 (d, 2H, *J*d = 4 Hz), 4.61, 4.58, 4.40, 4.34, 4.30, 4.27, 4.25 (7 m, 2H, 2 diastereomers in 2 rotameric forms), 3.76 (m, 1H), 3.02–2.82 (m, 4H), 2.57–2.46 (m, 1H), 2.29 (c, 3H), 1.83–1.40 (m, 6H). 13C{1H} NMR (125 MHz, DMSO-*d6*), 𝛿 (ppm): 168.02, 167.79, 157.62–157.55, 155.73–155.61, 150.08–149.95, 148.04–147.94, 148.36–147.46, 140.05, 145.57–145.43, 138.18, 128.56, 125.96, 120.32–119.96, 118.26, 106.76–106.35, 58.26, 58.20, 56.81, 52.13, 51.59, 47.72, 47.49, 43.35, 41.93, 37.61, 37.19, 35.99, 35.67, 31.39, 30.54, 30.44, 29.42, 25.96, 25.83, 25.47, 21.25. 19F NMR (376 MHz, DMSO-*d6*), 𝛿 (ppm): −117.71, −118.01 (2 m, 2 diastereomers), −135.71, −136.02 (2 m, 2 diastereomers), −143.21, −143.35 (2 m, 2 diastereomers). LC-MS APCI: *m/z* 338 [M + H]+. Anal. calcd for **12a,b** (1:1 mixture of isomers) C_24_H_26_F_3_N_3_O_4_S: C, 56,57; H, 5.14; N, 8.25; S, 6.29. Found: C, 56.70; H, 5.28; N, 8.08; S, 6.48%.

***(*** **3*S)*-2-[(3*R*)-3-Amino-4-(2,4,5-trifluorophenyl)butanoyl]-*exo*-2-azabicyclo[2.2.1]hepnane-3-carbonitrile *p*-toluenesulpfonate (12a)**

Compound **12a** (0.15 g, 84%) was prepared from **11a** (0.15 g, 0.35 mmol) by the analogy with **12a,b.** 1H NMR (400 MHz, DMSO-*d6*): 𝛿 7.94 (br.s, 3H, NH3+), 7.59–7.48 (m + d overlapped, 4H), 7.13 (d, 2H, *J*d = 4 Hz), 4.61, 4.24 (2 m, 1H, 2 rotameric forms), 4.40, 4.33 (2 m, 1H, 2 rotameric forms), 3.76 (m, 1H), 3.02–2.81 (m, 4H), 2.55-2.46 (m, 1H), 2.29 (c, 3H), 1.83–1.37 (m, 6H). 13C{1H} NMR (100 MHz, DMSO-*d6*), 𝛿 (ppm): 167.33, 157.31-157.44, 154.93–155.03, 149.56–149.90, 147.19–147.35, 145.56, 144.74–144.90, 137.77, 128.11, 125.51, 119.48–119.92, 117.79, 105.83–106.33, 57.77, 56.36, 51.70, 51.16, 47.33, 47.13, 42.95, 41.52, 37.11, 35.51, 35.32, 35.25, 31.14, 30.94, 30.11, 28.98, 25.52, 25.04, 20.79 LC/MS APCI: *m/z* 338 [M + H]+. Anal. calcd for C_24_H_26_F_3_N_3_O_4_S: C, 56,57; H, 5.14; N, 8.25; S, 6.29. Found: C, 56.70; H, 5.17; N, 8.13; S, 6.21%. HPLC: Shimadzu LC-2010, Column: Discovery Cyano 150 × 4.6 mm, 5 μm (Supelco), flow rate—0.9 mL/min, column temperature—25 °C, Detection—UV, 205 nm, sample volume—5 μl, elution mode—isocratic, chromatography time—20 min. Purity of **12a**—99.891%.

***(*** **3*R)*-2-[(3*R*)-3-Amino-4-(2,4,5-trifluorophenyl)butanoyl]-*exo*-2-azabicyclo[2.2.1]hepnane-3-carbonitrile *p*-toluenesulpfonate (12b)**

Compound **12b** (0.14 g, 78%) was prepared from **11b** (0.15 g, 0.35 mmol) by the analogy with **12a,b.** 1H NMR (400 MHz, DMSO-*d6*): 𝛿 7.92 (br.s, 3H, NH3+), 7.59 (m, 2H), 7.48 (d, 2H, *J*d = 8 Hz), 7.11 (d, 2H, *J*d = 8 Hz), 4.57, 4.27 (2 m, 1H, 2 rotameric forms), 4.38, 4.30 (2 m, 1H, 2 rotameric forms), 3.73 (m, 1H), 2.99–2.67 (m overlapped, 4H), 2.55–2.52 (m, 1H), 2.29 (c, 3H), 1.86–1.38 (m, 6H). 13C{1H} NMR (100 MHz, DMSO-*d6*), 𝛿 (ppm): 167.59, 167.42, 157.40–157.52, 154.98–155.08, 149.73–149.94, 147.16–147.45, 147.57, 144.74–144.93, 137.82, 128.17, 125.54, 119.48–119.81, 117.96, 105.88–106.39, 57.85, 56.29, 51.87, 51.18, 47.51, 42.94, 41.55, 37.21, 35.22, 34.97, 30.85, 30.05, 29.13, 25.43, 25.13, 20.85. LC-MS AP-ES: *m/z* 338 [M + H]+. Anal. calcd for C_24_H_26_F_3_N_3_O_4_S: C, 56,57; H, 5.14; N, 8.25; S, 6.29. Found: C, 56.87; H, 5.16; N, 8.12; S, 6.50%.

***(*** **3*R,S)*-2-[(3*R*)-3-Amino-4-(2,4,5-trifluorophenyl)butanoyl]-*endo*-2-azabicyclo[2.2.1]hepnane-3-carbonitrile *p*-toluenesulpfonate (12c,d)**

Compound **12c,d** as p-TSA-salt (0.29 g, 83%) was prepared from **11c,d** (0.30 g, 0.69 mmol) by the analogy with **12a,b.** 1H NMR (400 MHz, DMSO-*d6*): 𝛿 7.95 (br.s, 3H, NH3+), 7.57–7.48 (m + d overlapped, 4H), 7.13 (d, 2H, *J*d = 8 Hz), 4.86, 4.54, 4.47, 4.28 (4 m, 2H, 2 diastereomers in 2 rotameric forms), 3.83–3.76 (m, 1H), 3.02–2.56 (m overlapped, 5H), 2.29 (c, 3H), 1.73–1.50 (m, 6H). 13C{1H} NMR (100 MHz, DMSO-*d6*), 𝛿 (ppm): 167.01, 166.55, 157.38–157.49, 154.96–155.08, 149.68–150.06, 147.16–147.50, 145.50, 144.74–144.91, 137.87, 128.17, 125.54, 119.50–119.88, 117.63, 105.85–106.35, 58.01, 55.93, 51.34, 50.70, 47.52, 47.43, 47.34, 41.50, 39.92, 37.28, 35.79, 35.07, 34.39, 31.03, 30.91, 29.78, 29.07, 23.82, 23.47, 20.84. LC/MS APCI: *m/z* 338 [M + H]+. Anal. calcd for **12c,d** (1:1 mixture of isomers) C_24_H_26_F_3_N_3_O_4_S: C, 56.57; H, 5.14; N, 8.25; S, 6.29. Found: C, 56.54; H, 5.32; N, 8.01; S, 6.35%.

## 4. Conclusions

Here we show that pseudo peptides containing trifluoro-substituted aromatic β-amino acid and the proline functional analogue (with a labile nitrile group) inhibit DPP-4. Interestingly, the *Exo*-isomeric derivatives within all these groups were more potent against DPP-4 than the corresponding *endo*-analogues. Moreover, we found that the S-configuration of the amine part was the most favourable in all the experiments. The maximum similarity score of 0.38 was obtained for compounds **12a** and evogliptin among all the pairs (a synthesised compound + already known drug). Therefore, all our synthesised compounds can be considered significantly different compared to marketed gliptins.

The IC50 of compound **12a** is in the same range as for reference compounds, and this is consistent with literature data [9,48,52,53,54,55]. As for DPP-8, **12a** showed feeble inhibitory activity in the concentration range 10^−3^–10^−6^ M, and for a homologous enzyme DPP-9 in concentration 10^−3^ M. This is significantly below the estimated therapeutic range and is consistent with literature data for already known DPP-4 inhibitor compounds [55]. This makes it possible to predict the absence of side effects associated with undesirable inhibition of homologous enzymes DPP-8 and DPP-9.

We revealed and proved the presence of *E/Z*-rotameric forms in a solution. The energy barrier of free rotation around the amide bond is large enough, and it is possible to overcome it only at a temperature of more than 140 °C. At a temperature close to 140 °C, a partial coalescence of the abovementioned signals was observed. The initial studies of **12a** suggest that this compound could be less toxic and more stable in aqueous solutions than marketed gliptins.

## 5. Patents

Patent (Russian Federation), Issue No. 2018134266, date of issue 28.09.2018, date of registration in the State Register of Inventions (RU) 24.01.2020, date of expiry 28.09.2038. Dipeptidyl peptidase-4 inhibitor for treating type 2 diabetes mellitus, compounds (versions)//Patentee: Neobiotek LLC (Moscow, Russia). Authors: Trukhan V.M., Zinevich T.V., Maslov I.O., Kirichenko O.G.

## Figures and Tables

**Table 1:** The U.S. Food and Drug Administration (FDA) structures and approval of the most common DPP-4 inhibitors.

Compound	FDA	Structure
Sitagliptin	+	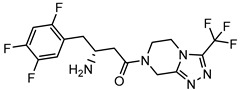
Saxagliptin	+	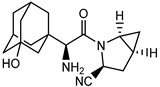
Alogliptin	+	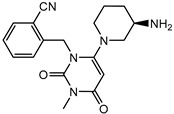
Linagliptin	+	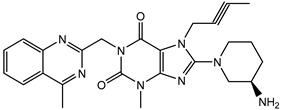
Vildagliptin	−	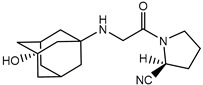
Teneligliptin	−	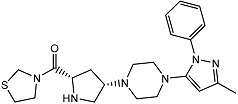
Trelagliptin	−	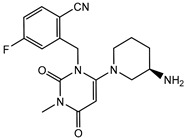
Evogliptin	−	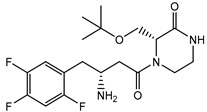
Omarigliptin	−	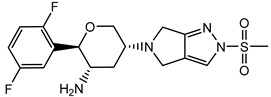

Approved (+); U.S. Food and Drug Administration (FDA); Not approved (−).

**Table 2:** Scoring results of re-docked reference structures, comparison with best-fitting compound **12a** and non-active **12b–d**. Higher risk of potential cardiotoxicity is highlighted in yellow and lower risk of potential cardiotoxicity is highlighted in green.

Structure	GlideScore	Emodel	∆G_bind_ (kcal/mol)	QPlogHERG
Sitagliptin	−5.80	−63.96	−17.05	−4.351
Trelagliptin	−5.68	−54.94	−16.35	−5.078
Evogliptin	−5.84	−53.11	−17.02	−3.032
Vildagliptin	−5.99	−56.58	−16.60	−2.987
12a	−5.74	−59,69	−17.39	−2.884
12b	−4.01	−40.56	−5.14	−3.191
12c	−3.69	−37.24	−9.09	−3.010
12d	−3.70	−45.46	−3.43	−3.393

Predicted IC50 value for blockage of HERG K+ channels (QPlogHERG).

**Table 3:** DPP-4-, DPP-8-, and DPP-9-inhibitory activities of target compounds **12a–d**.

Structure	DPP-4 IC_50_, nM	DPP-8 IC_50_, nM	DPP-9 IC_50_, nM
**12a** (*S-exo*)	16.8 ± 2.2	>1000	>1000
**12b** (*R-exo*)	>1000	-	-
**12c** (*S-endo*): **12d** (*R-endo*), 1:1 mixture	>1000	-	-

Dipeptidyl peptidase (DPP).

## Data Availability

The data presented in this study are available in the article and in the supplementary materials.

## Supplementary Materials

The following supporting information can be downloaded at: https://www.mdpi.com/article/10.3390/ph15030273/s1, Supplementary file S1_NMR. Supplementary file S2_mass_spectra.
