# Supplementary material for: Design, Synthesis and Biological Evaluation of Neogliptin, a Novel 2-Azabicyclo[2.2.1]heptane-Based Inhibitor of Dipeptidyl Peptidase-4 (DPP-4)
Source: Pharmaceuticals (Basel). 2022 Feb 22;15(3):273. doi: 10.3390/ph15030273 (PMC8949241; doi:10.3390/ph15030273)
Supplement: Supplementary file 1 [file pharmaceuticals-15-00273-s001.zip › LCMS.zip!/1H NMR for 4a,b.pdf]

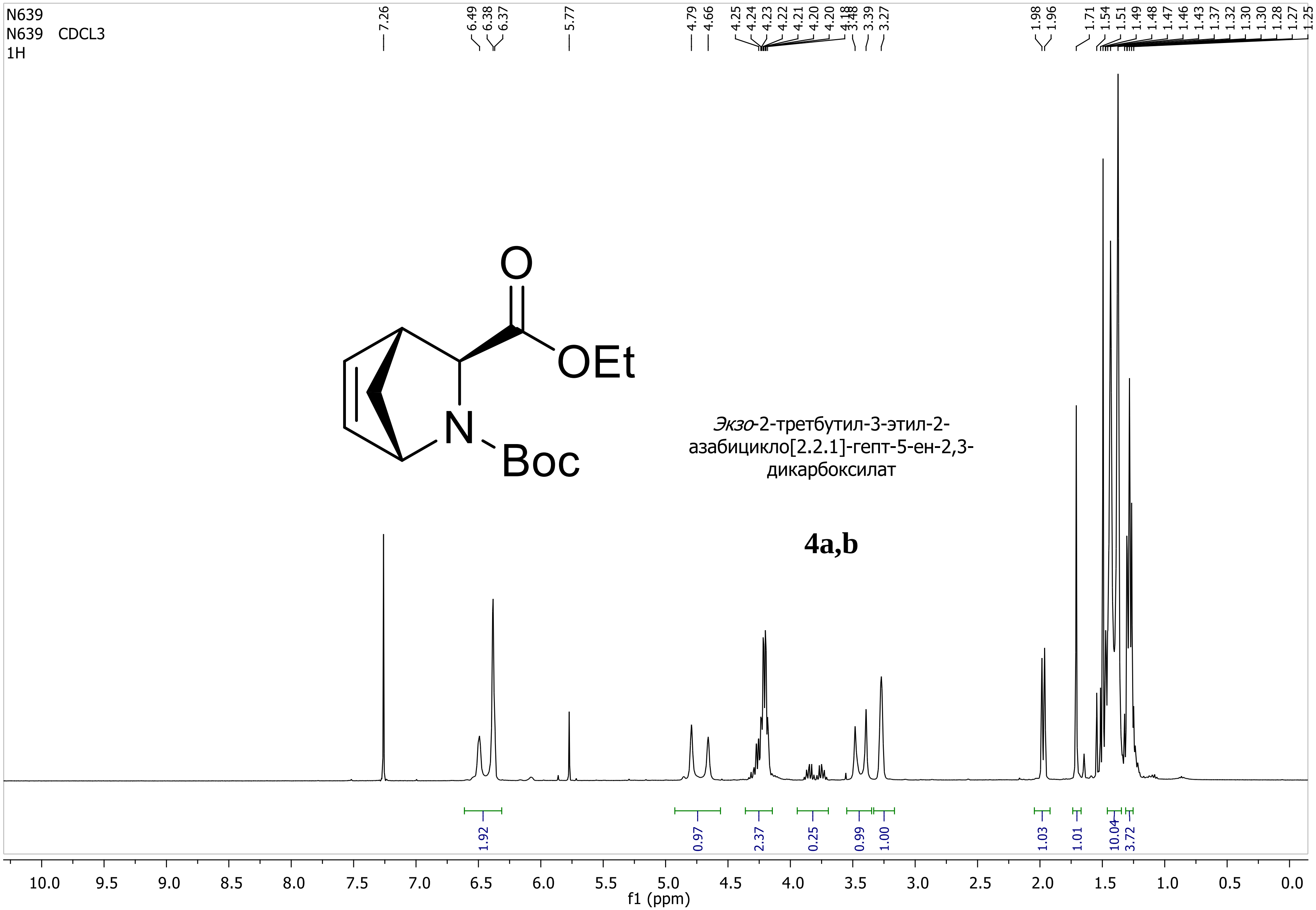
N639
N639 CDCL3
1H
7.26
6.49 6.38 6.37
5.77
4.79 4.66
4.25 4.24 4.23 4.22 4.21 4.20 4.20 4.18
3.48 3.39 3.27
1.98 1.96
1.71 1.54 1.51 1.49 1.48 1.47 1.46 1.43 1.37 1.32 1.30 1.30 1.28 1.27 1.25
O
OEt
N
Boc
Экзо-2-третбутил-3-этил-2-азабицикло[2.2.1]-гепт-5-ен-2,3-дикарбоксилат
4a,b
1.92 0.97 2.37 0.25 0.99 1.00 1.03 1.01 10.04 3.72
10.0 9.5 9.0 8.5 8.0 7.5 7.0 6.5 6.0 5.5 5.0 4.5 4.0 3.5 3.0 2.5 2.0 1.5 1.0 0.5 0.0
f1 (ppm)

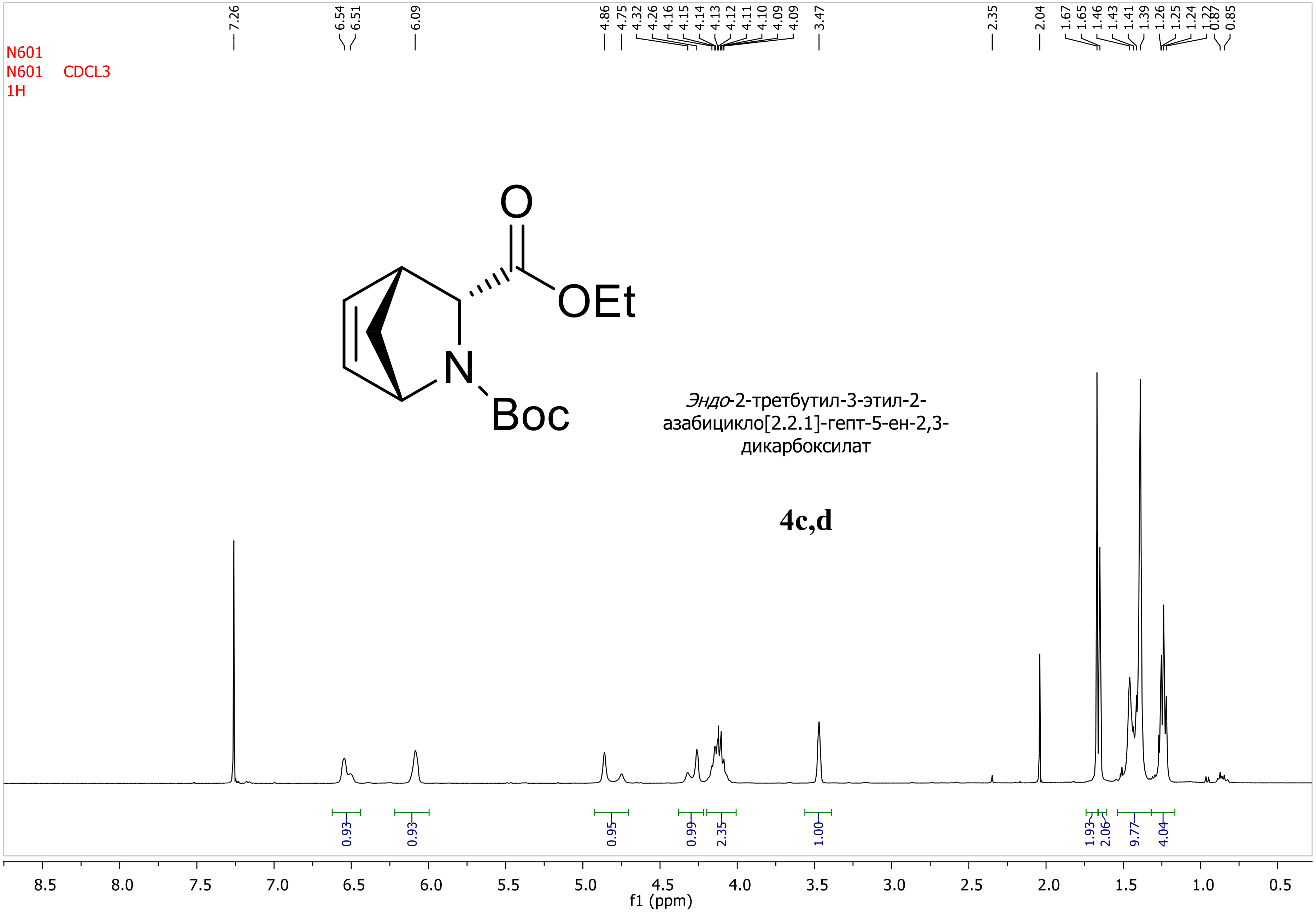
N601
N601 CDCL3
1H
7.26
6.54
6.51
6.09
4.86
4.75
4.32
4.26
4.16
4.15
4.14
4.13
4.12
4.11
4.10
4.09
4.09
3.47
2.35
2.04
1.67
1.65
1.46
1.43
1.41
1.39
1.26
1.25
1.24
1.22
0.87
0.85
O
OEt
N
Boc
Эндо-2-третбутил-3-этил-2-азабицикло[2.2.1]-гепт-5-ен-2,3-дикарбоксилат
4c,d
0.93
0.93
0.95
0.99
2.35
1.00
1.93
2.06
9.77
4.04
8.5
8.0
7.5
7.0
6.5
6.0
5.5
5.0
4.5
4.0
3.5
3.0
2.5
2.0
1.5
1.0
0.5
f1 (ppm)
