# Supplementary material for: Design, Synthesis and Biological Evaluation of Neogliptin, a Novel 2-Azabicyclo[2.2.1]heptane-Based Inhibitor of Dipeptidyl Peptidase-4 (DPP-4)
Source: Pharmaceuticals (Basel). 2022 Feb 22;15(3):273. doi: 10.3390/ph15030273 (PMC8949241; doi:10.3390/ph15030273)
Supplement: Supplementary file 1 [file pharmaceuticals-15-00273-s001.zip › LCMS.zip!/HPLC_12b.pdf]

HPLC: Shimadzu LC-2010, Column: Discovery Cyano 150 x 4,6 mm, 5 mkm (Supelco), flow rate - 0.9 ml / min, column temperature - 25 °C, Detection - UV, 205 nm, sample volume - 5 µl, elution mode - isocratic, chromatography time - 20 minutes. Purity of **12a** - 99.891%.

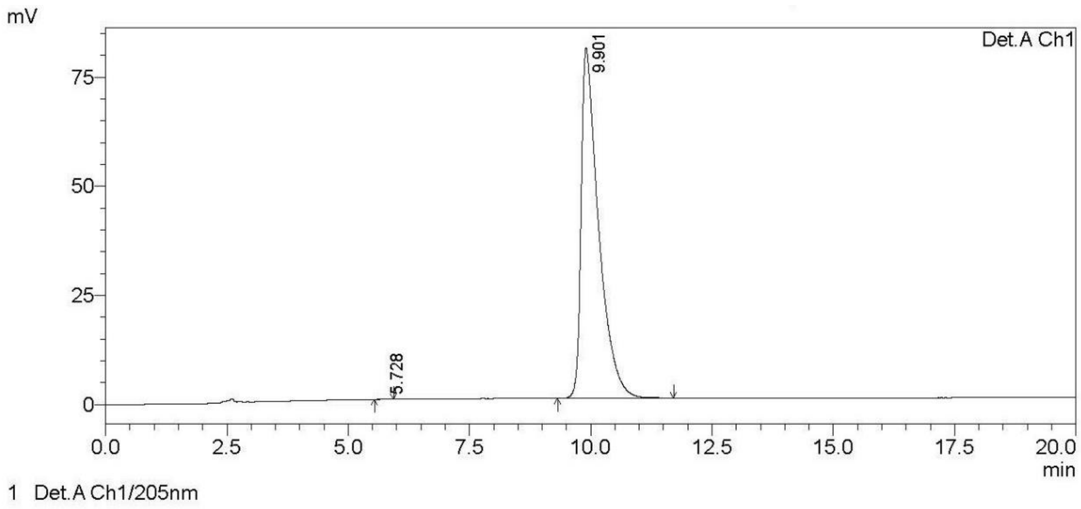

PeakTable

Detector A Ch1 205nm

Peak#	Ret. Time	Area	Area %
1	5.728	2186	0.109
2	9.901	2004128	99.891
Total		2006314	100.000
