# Supplementary material for: Design, Synthesis and Biological Evaluation of Neogliptin, a Novel 2-Azabicyclo[2.2.1]heptane-Based Inhibitor of Dipeptidyl Peptidase-4 (DPP-4)
Source: Pharmaceuticals (Basel). 2022 Feb 22;15(3):273. doi: 10.3390/ph15030273 (PMC8949241; doi:10.3390/ph15030273)

Design, synthesis and biological evaluation of Neogliptin, a novel 2-azabicyclo[2.2.1]hepnane-based inhibitor of dipeptidyl peptidase-4 (DPP-4)

Concentration-response curves of the synthesized compounds **12a** (neogliptin), **12b** and **12c**+**12d** (1:1 mixture of diastereomers).

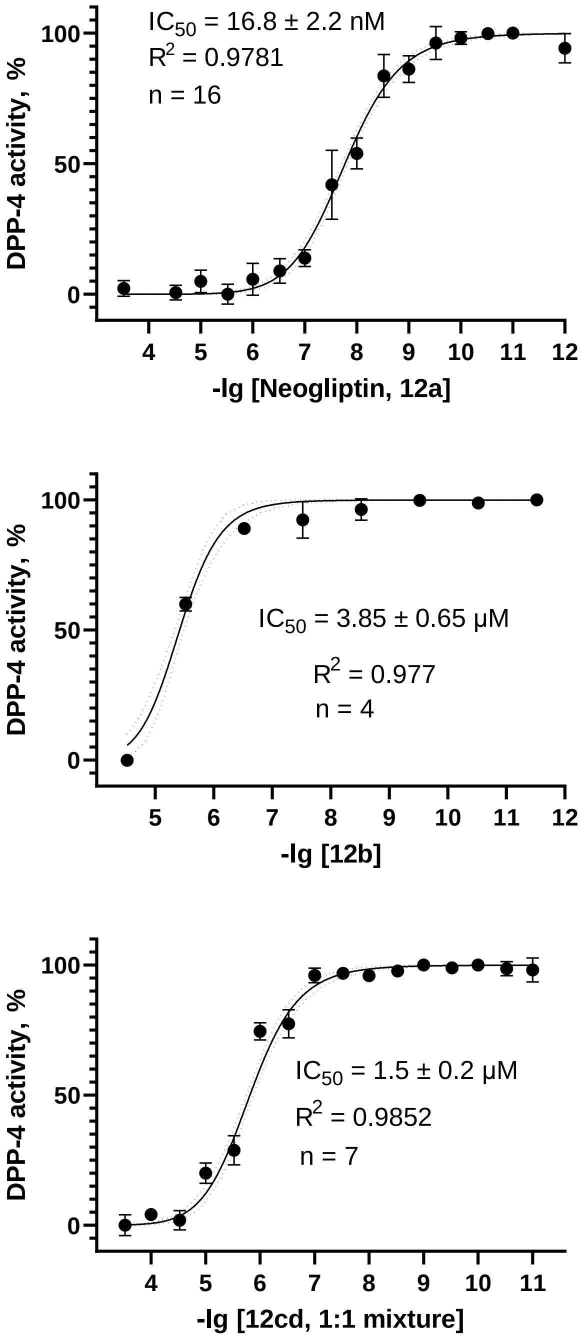

Concentration-response curves of the four marketed DPP4 inhibitors (data was obtained using the same assay conditions).

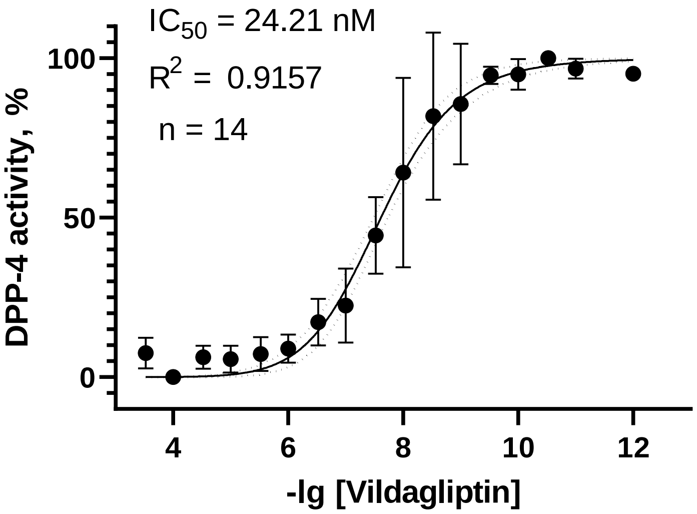

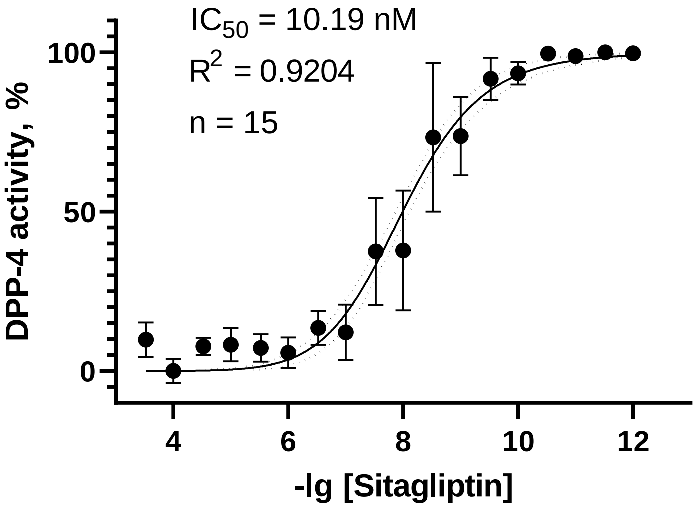

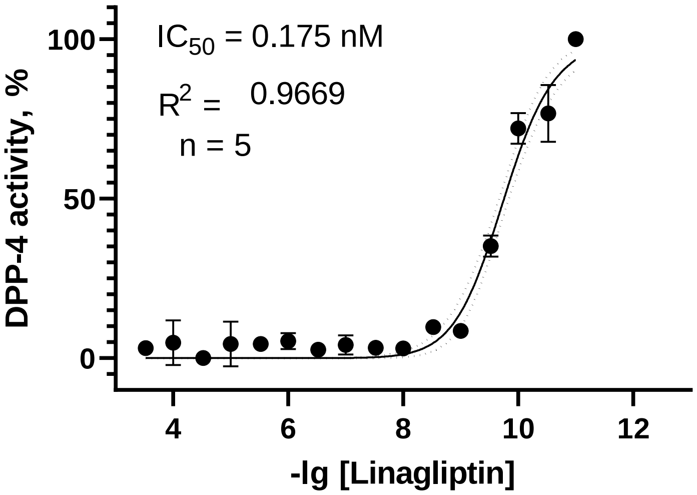

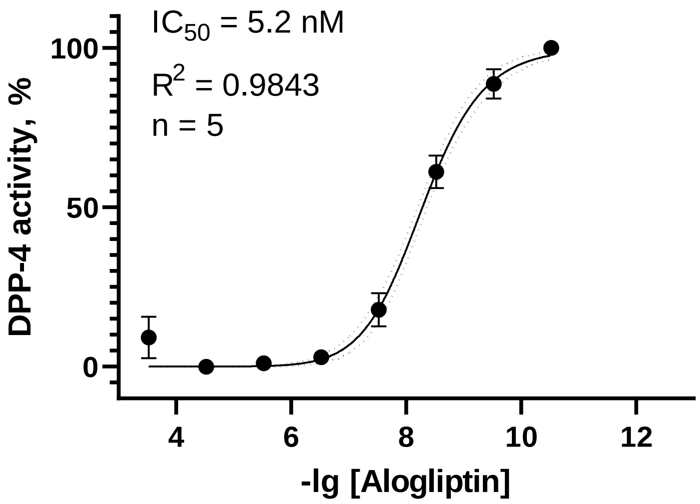

NMR-spectra of the key intermediates and target compounds.

5a,b

^{1}H NMR

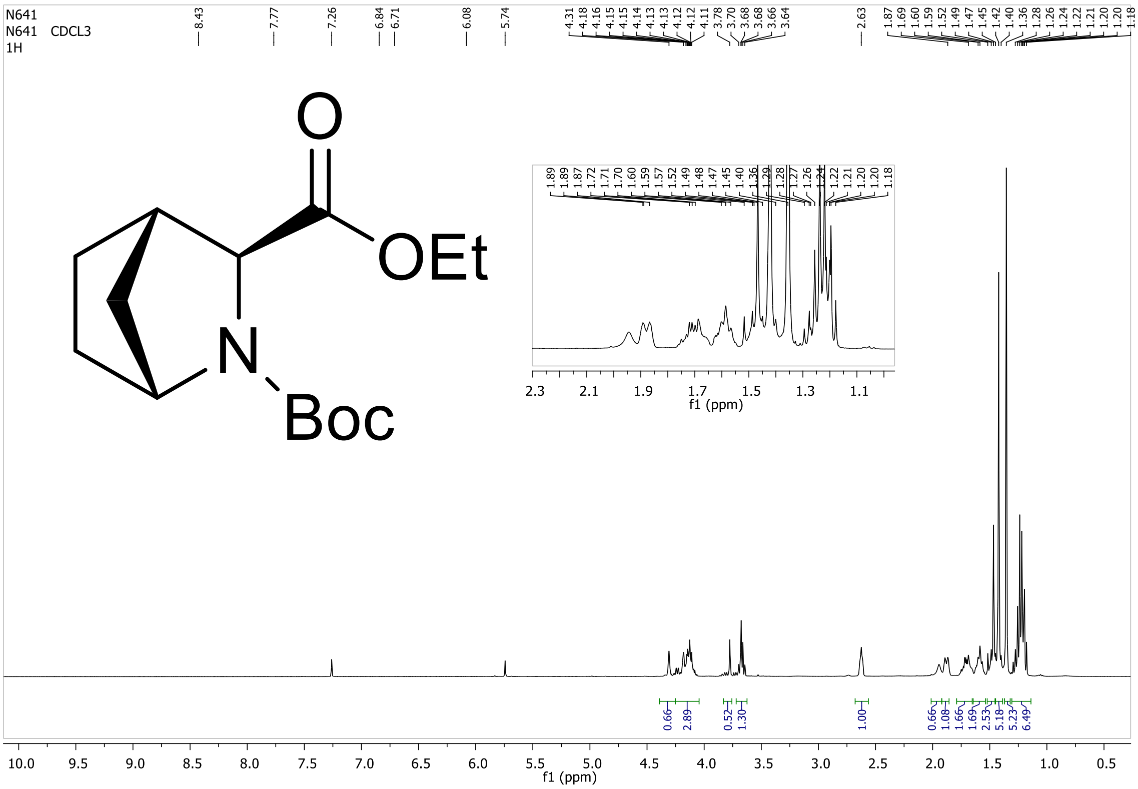

5c,d

^{1}H NMR

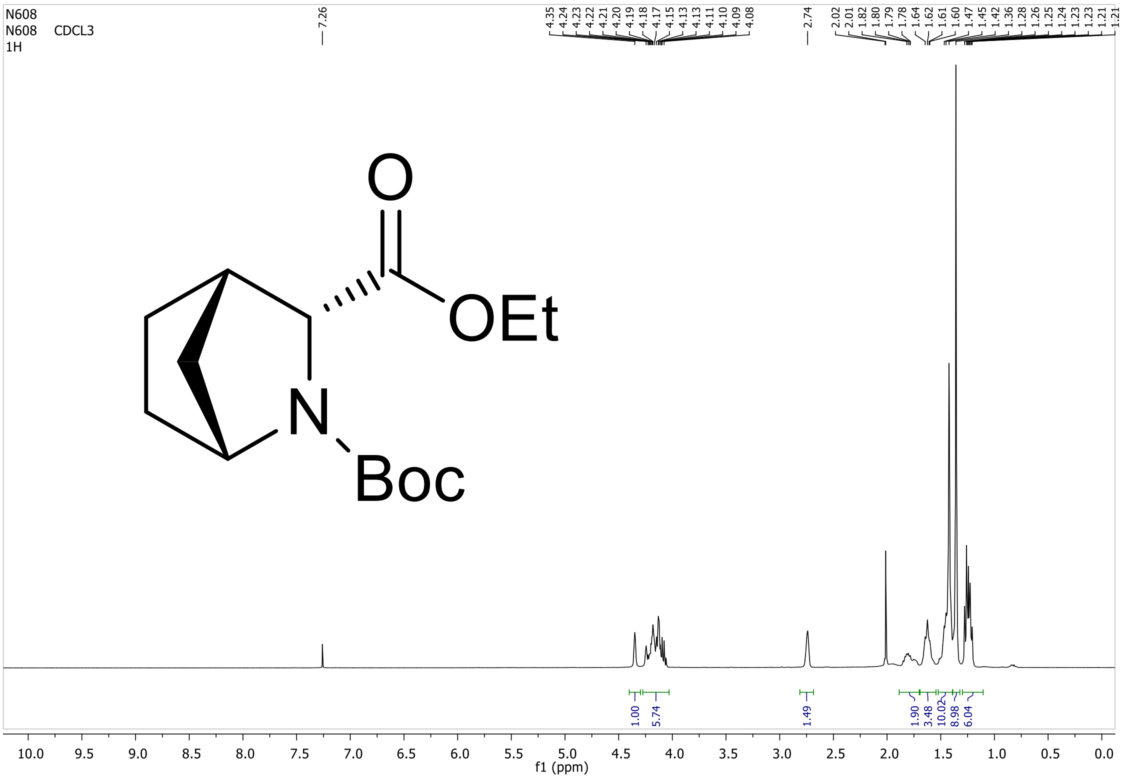

6a,b

^{1}H NMR

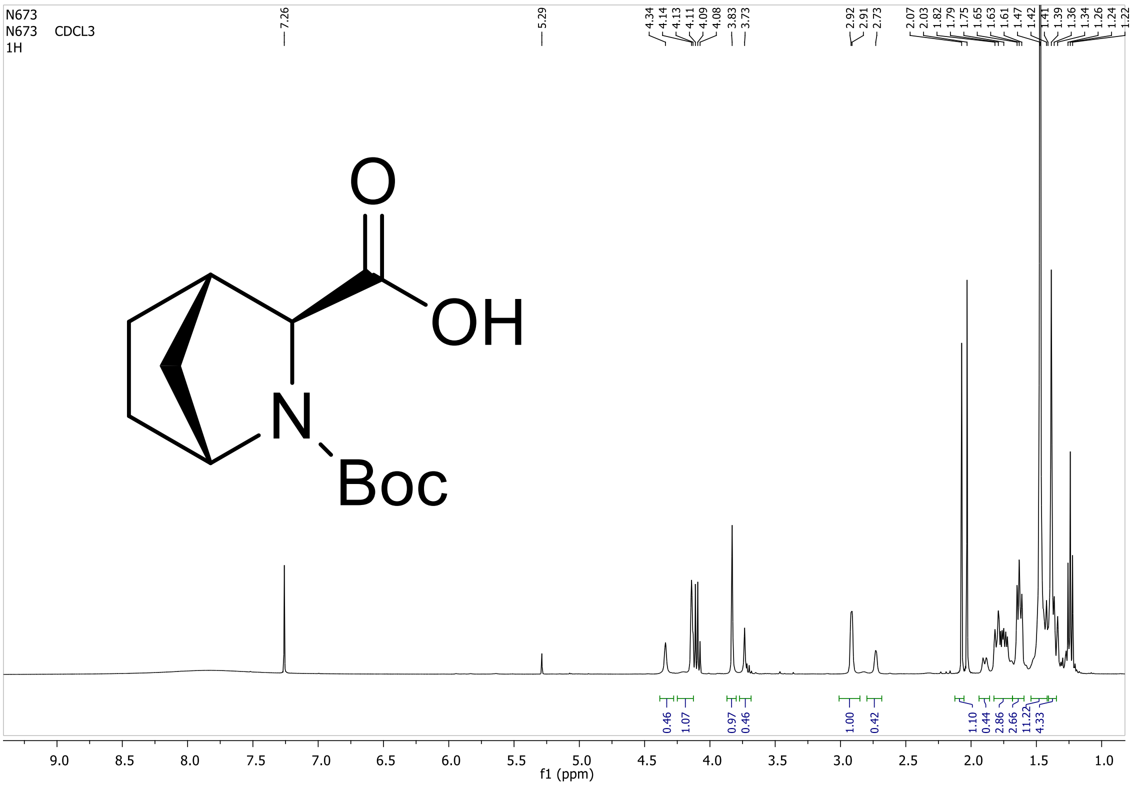

6c,d

^{1}H NMR

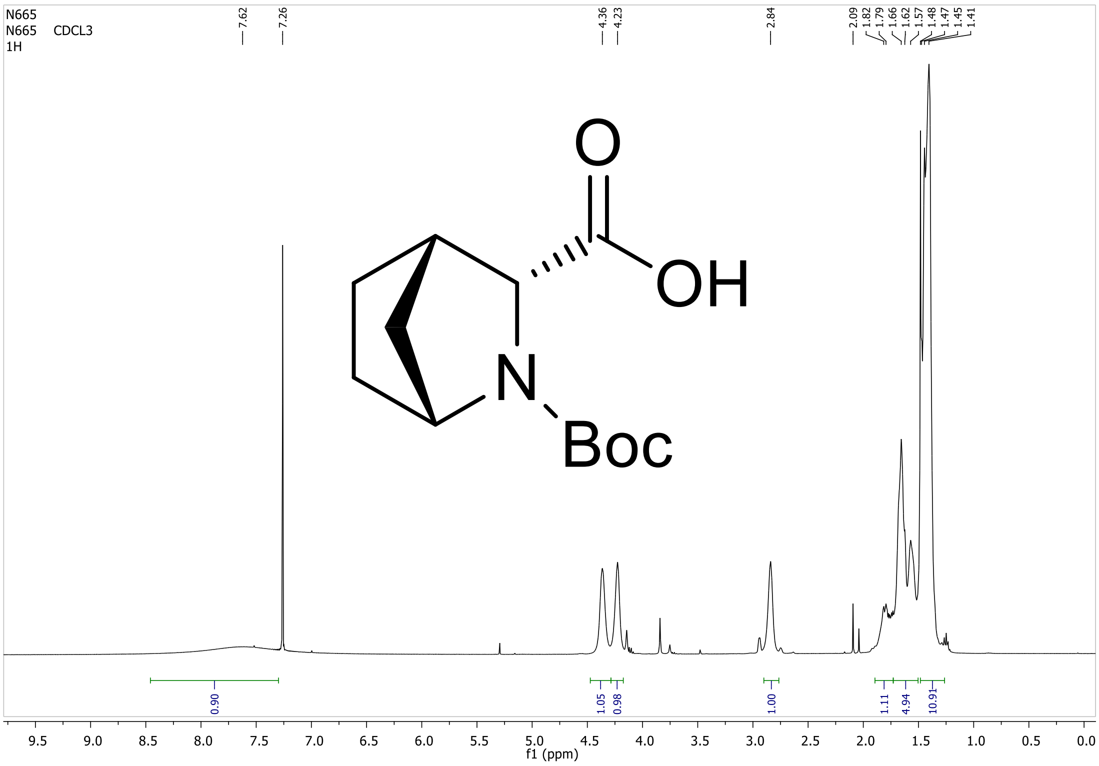

7a,b

^{1}H NMR

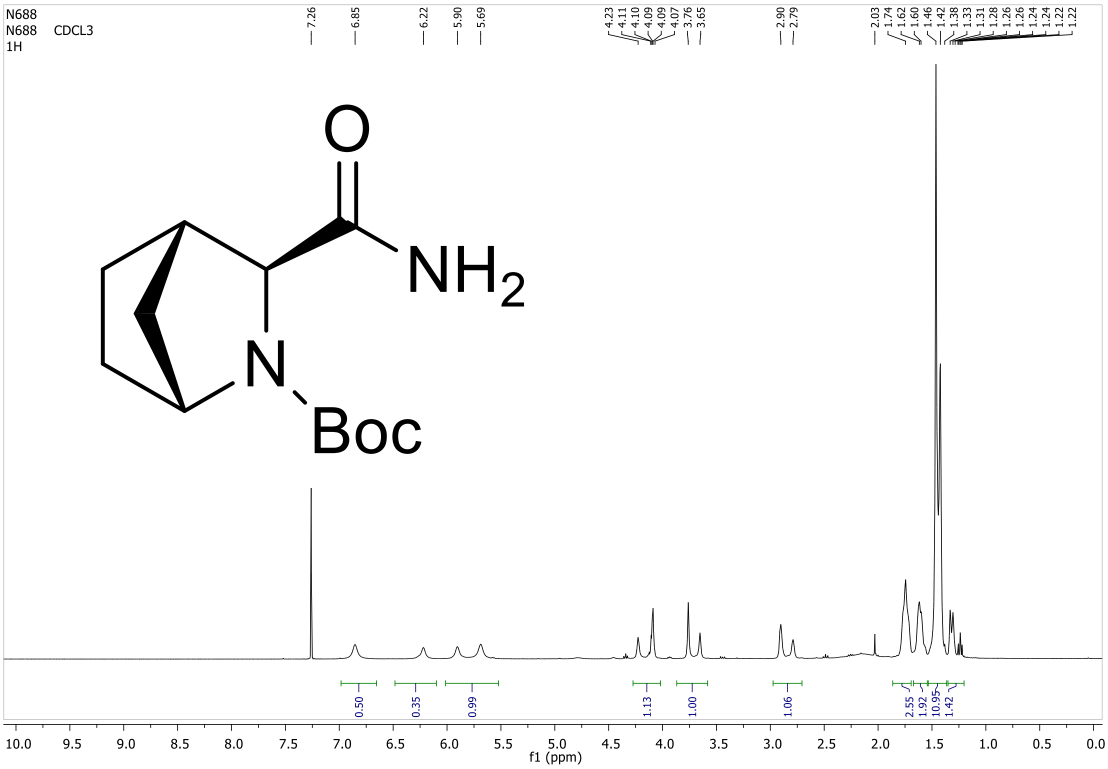

7c,d

^{1}H NMR

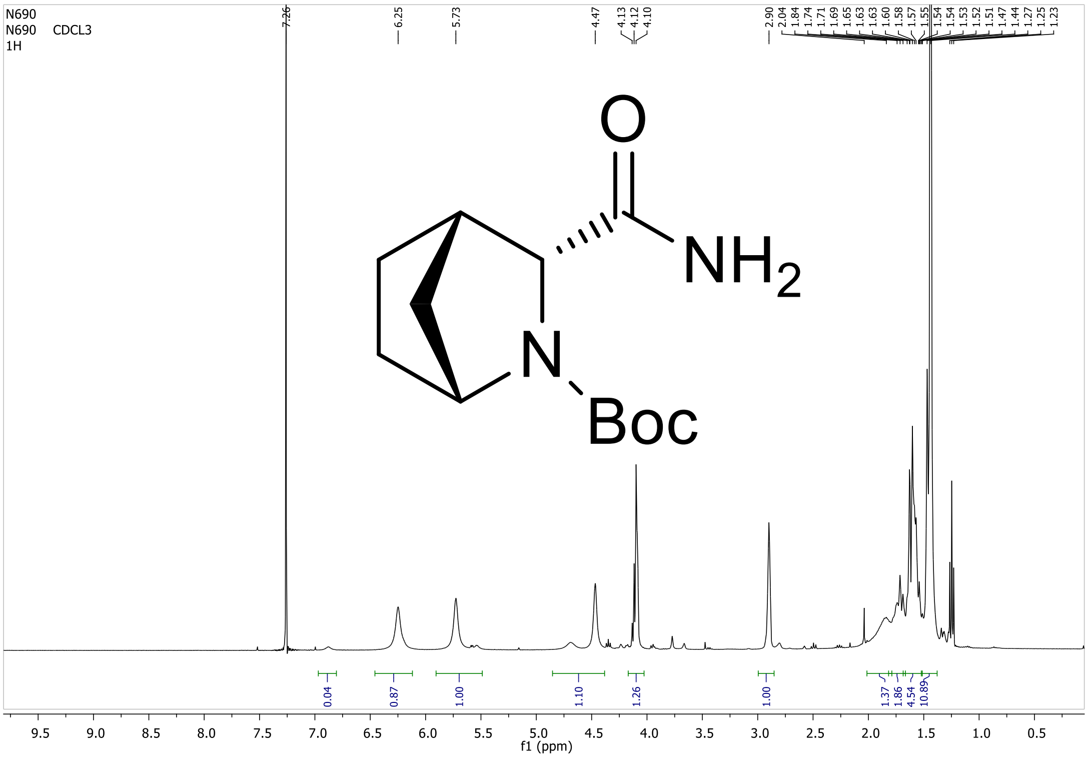

8a,b

^{1}H NMR

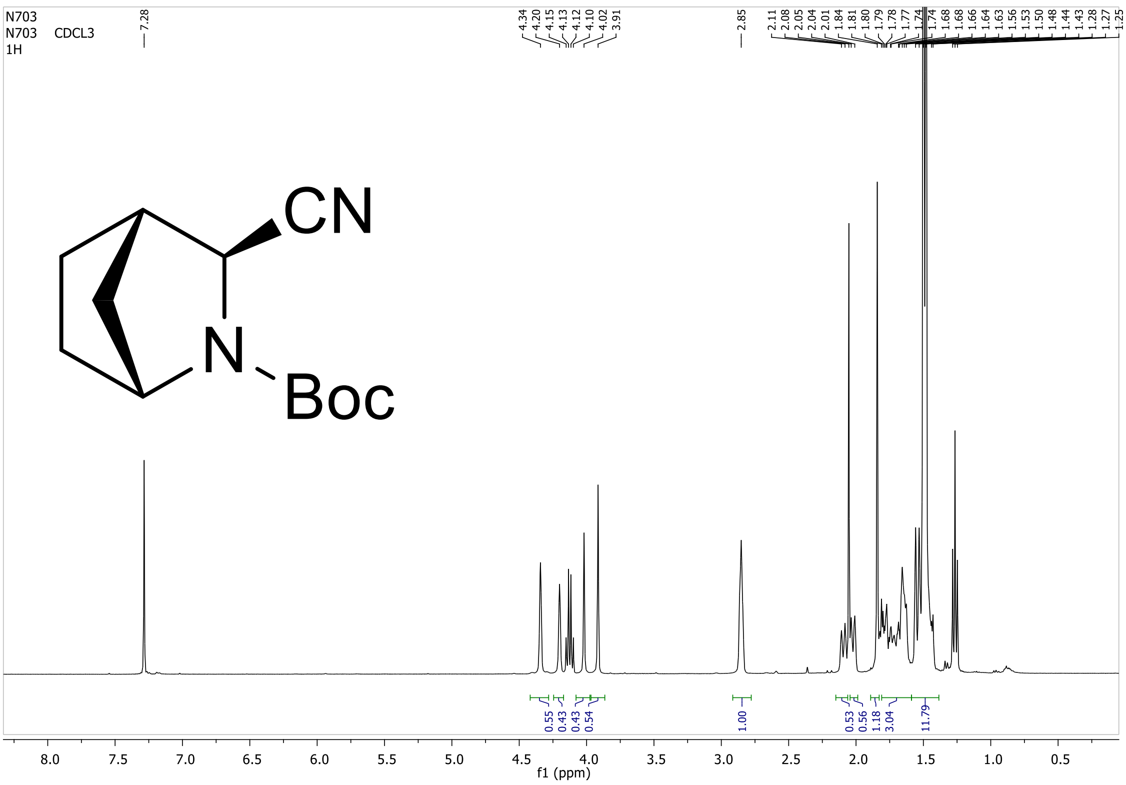

$^{13}C\{^1H\}$ NMR

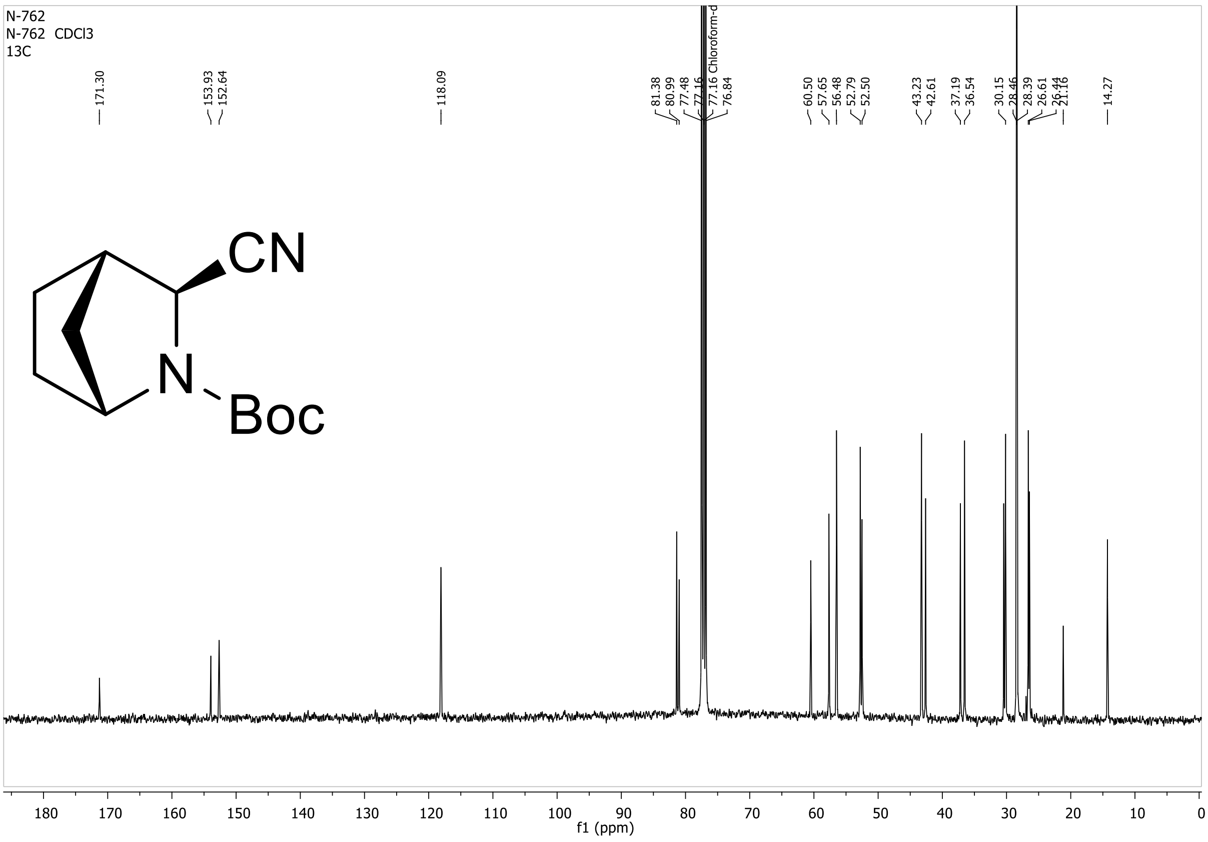

8c,d

^1H NMR

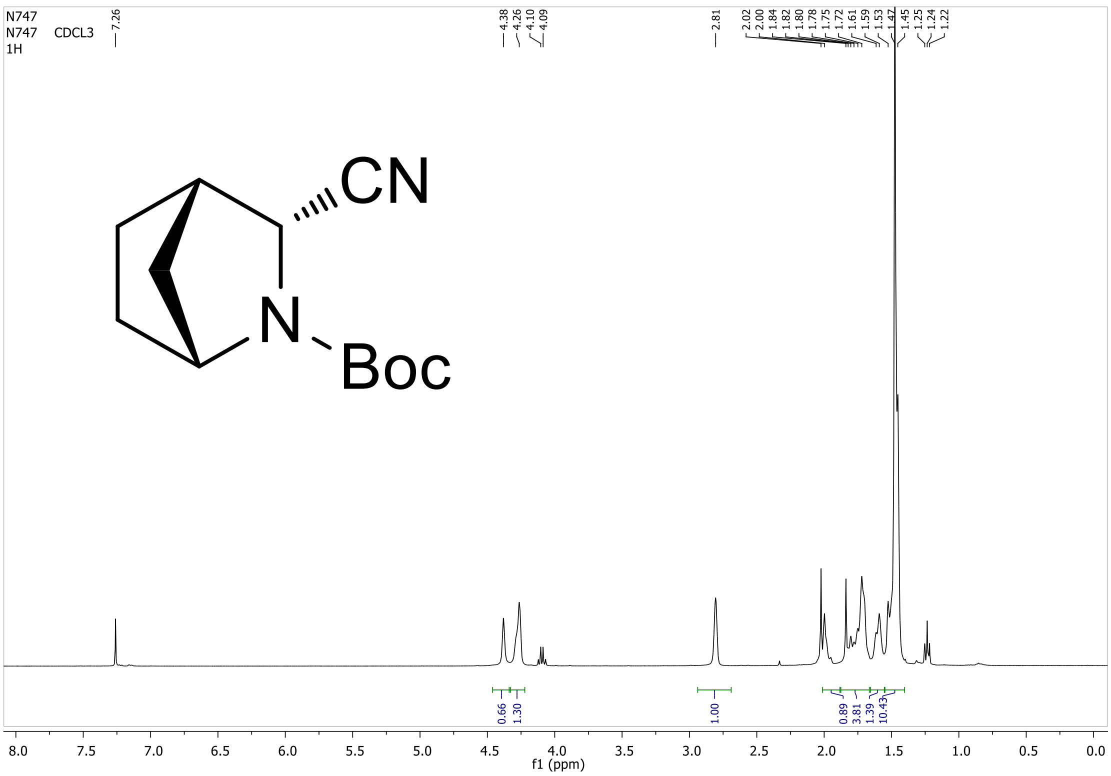

$^{13}C\{^{1}H\}$ NMR

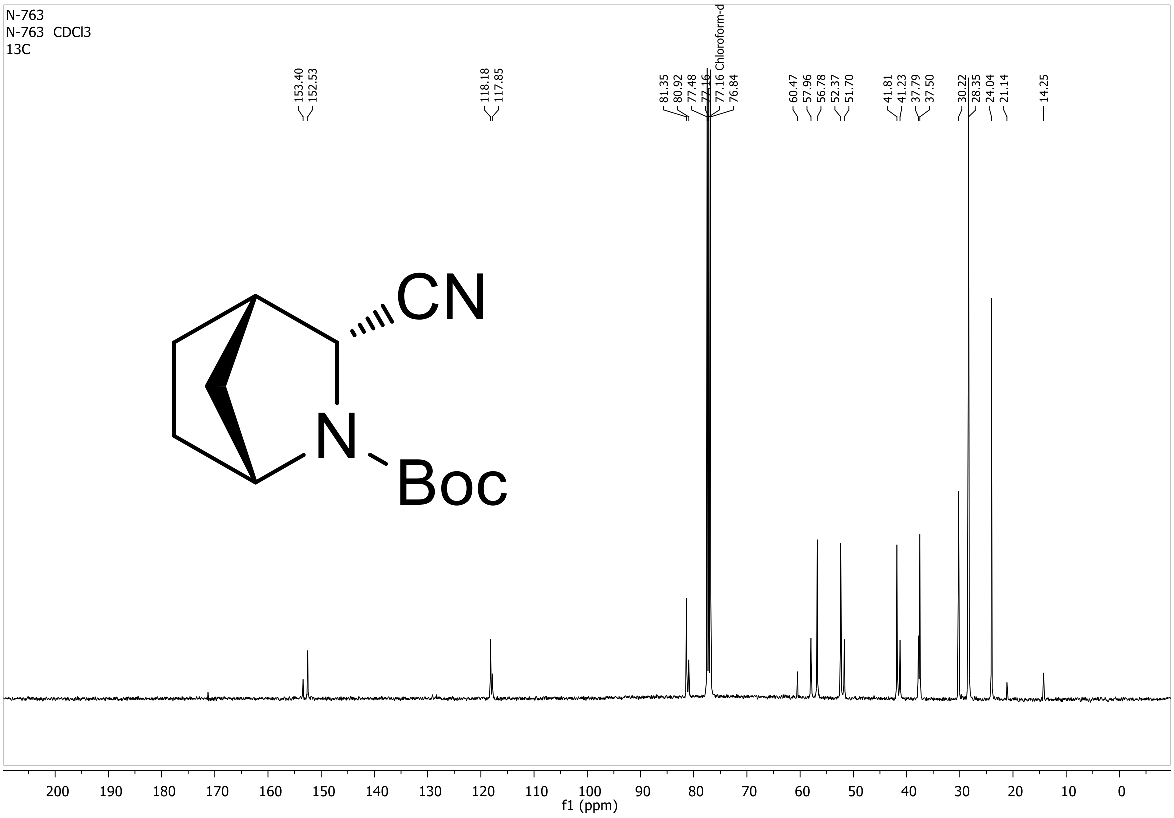

9a,b

^{1}H NMR

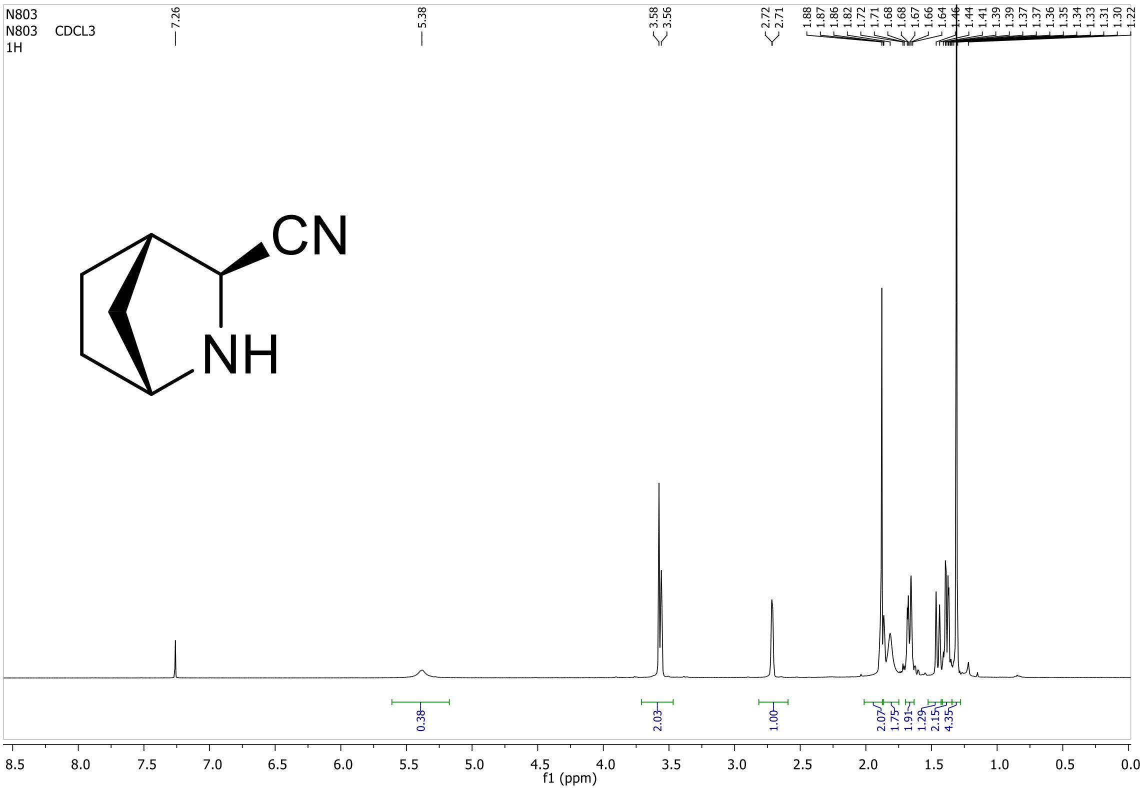

$^{13}C\{^{1}H\}$ NMR

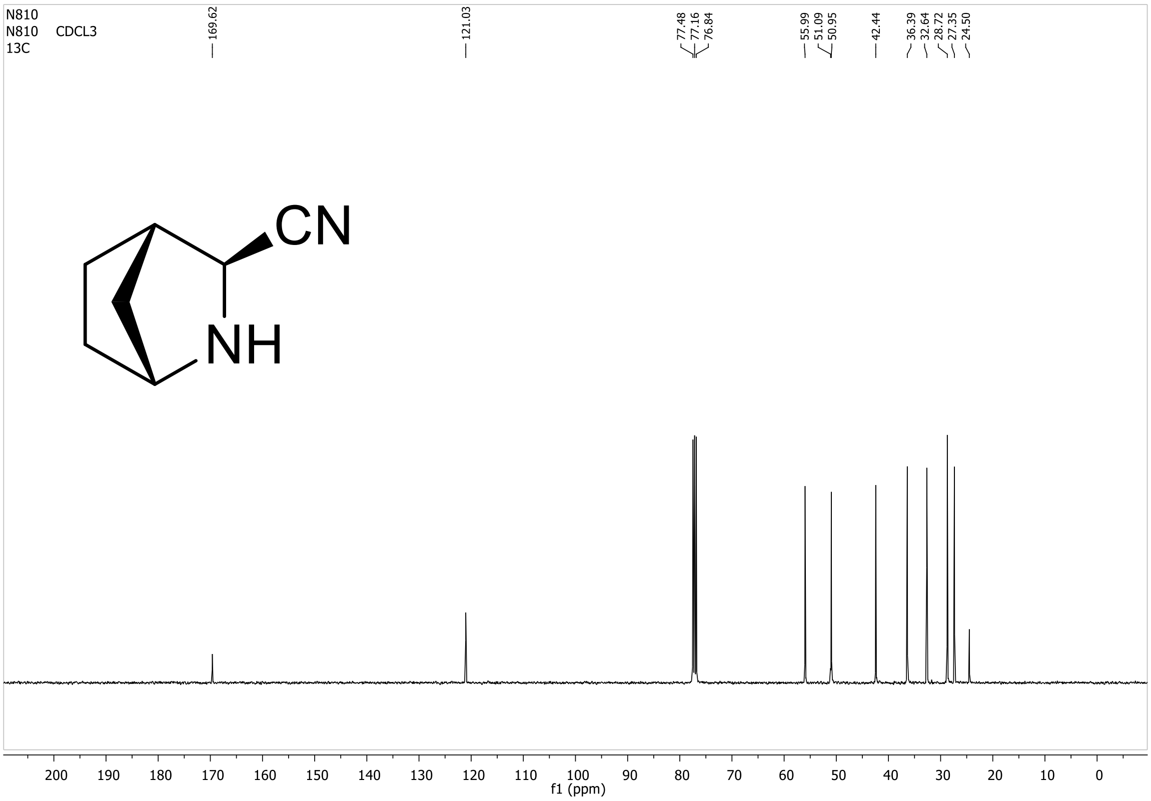

9c,d

^{1}H NMR

Supplementary material 1

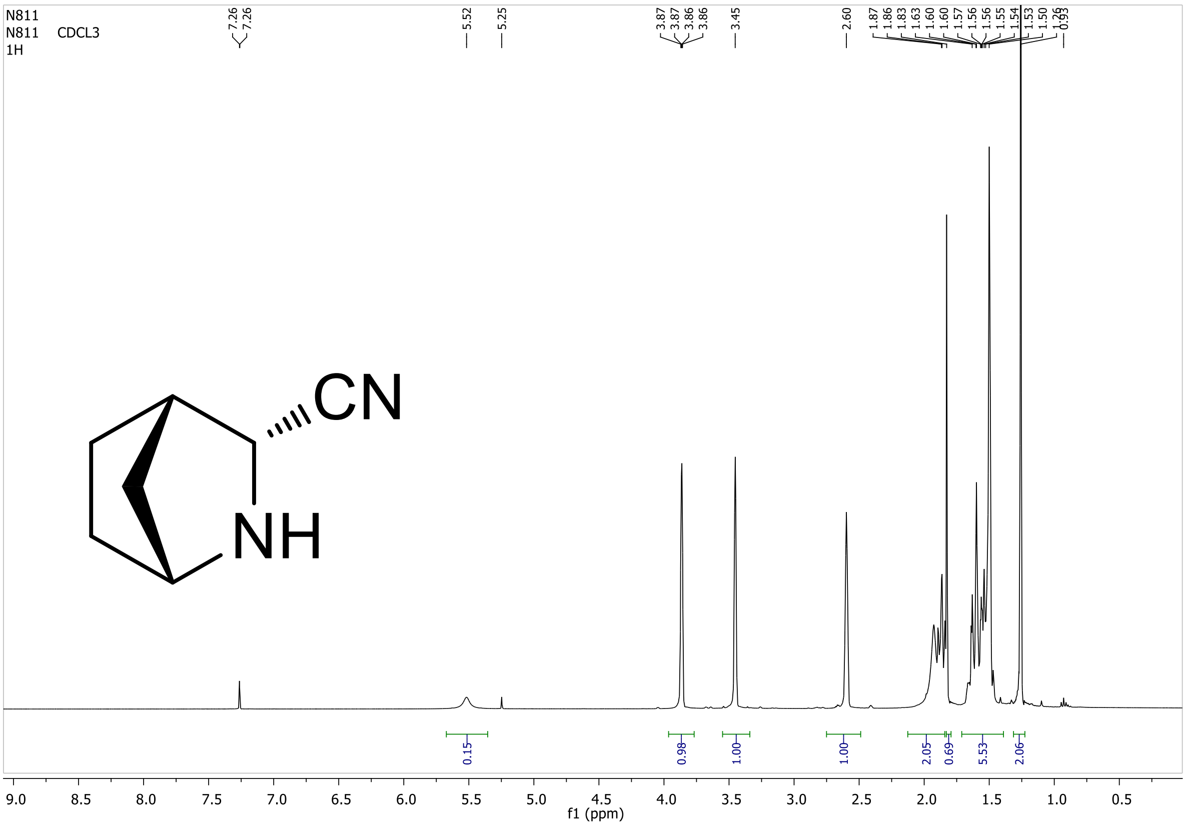

$^{13}C\{^{1}H\}$ NMR

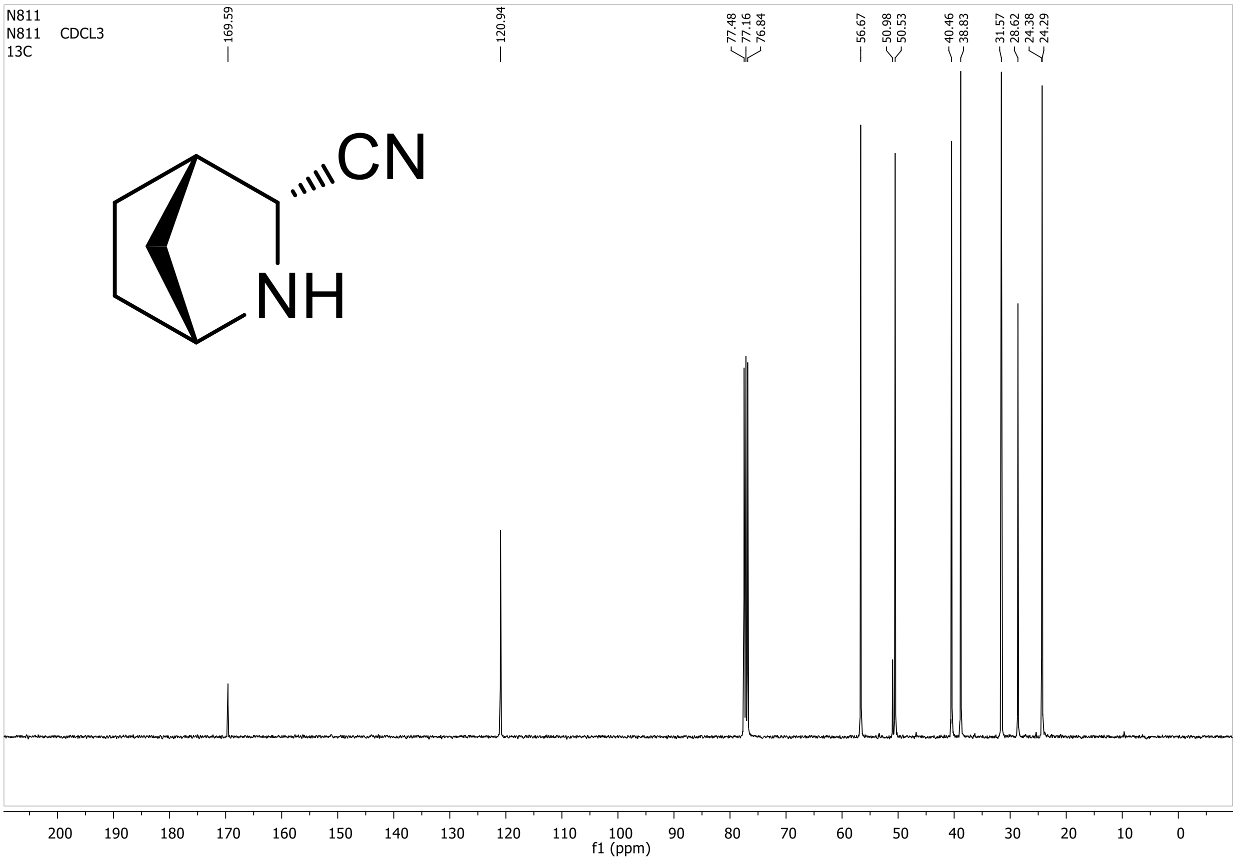

Supplementary material 1

11a,b

^{1}H NMR

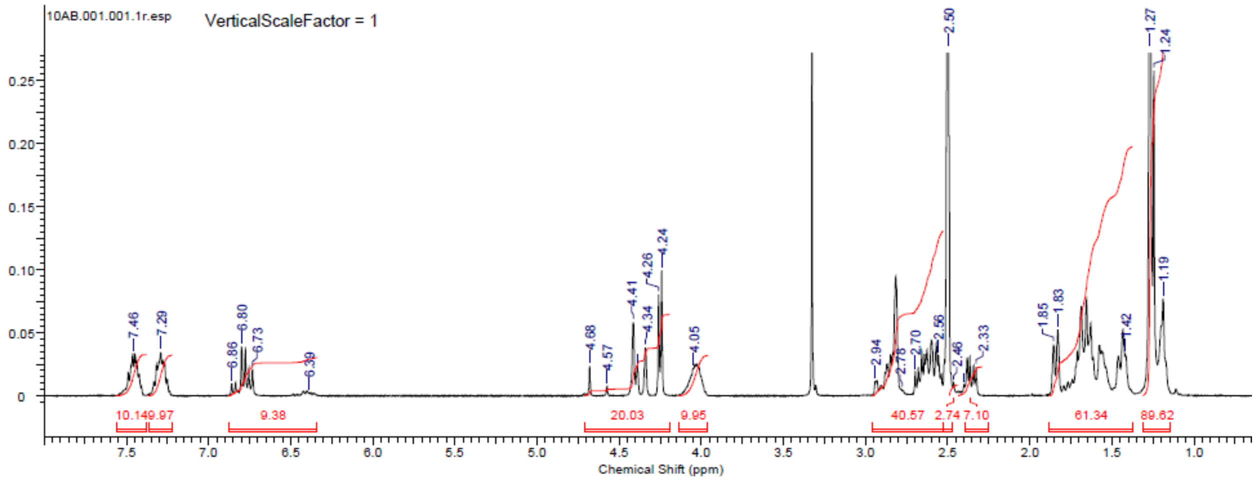

^{13}C{^{1}H} NMR

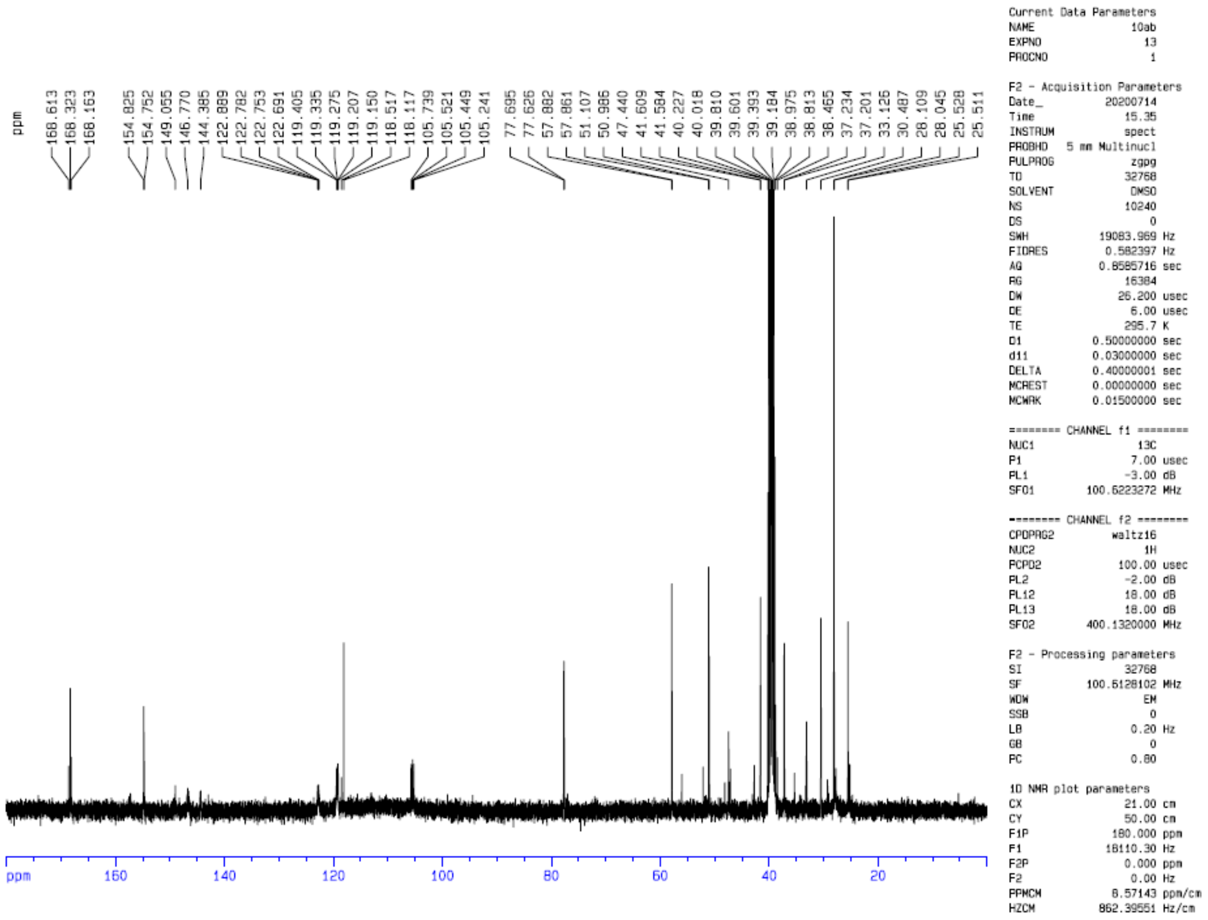

Supplementary material 1

11a

^{1}H NMR

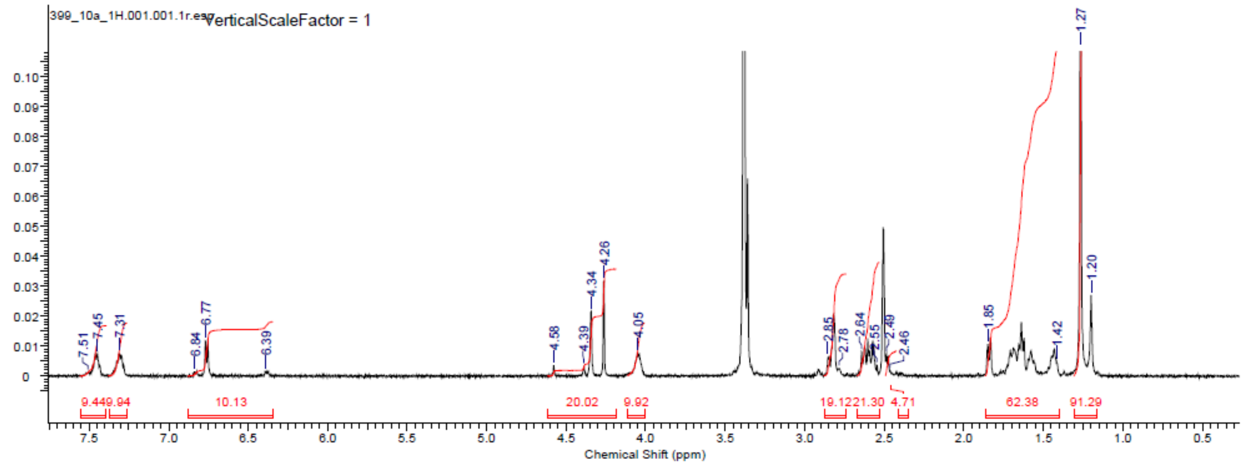

11b

^{1}H NMR

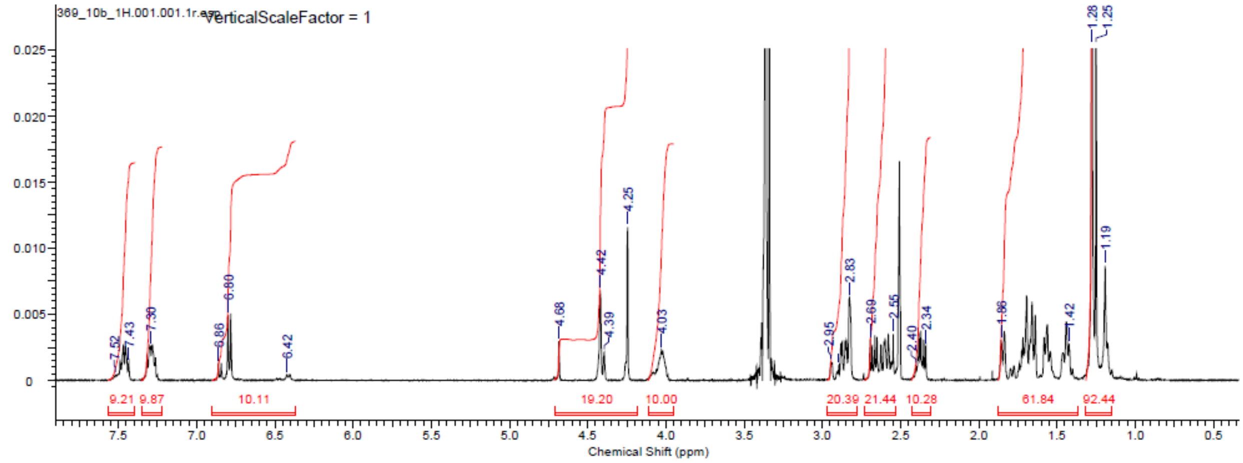

11c,d

^{1}H NMR

Supplementary material 1

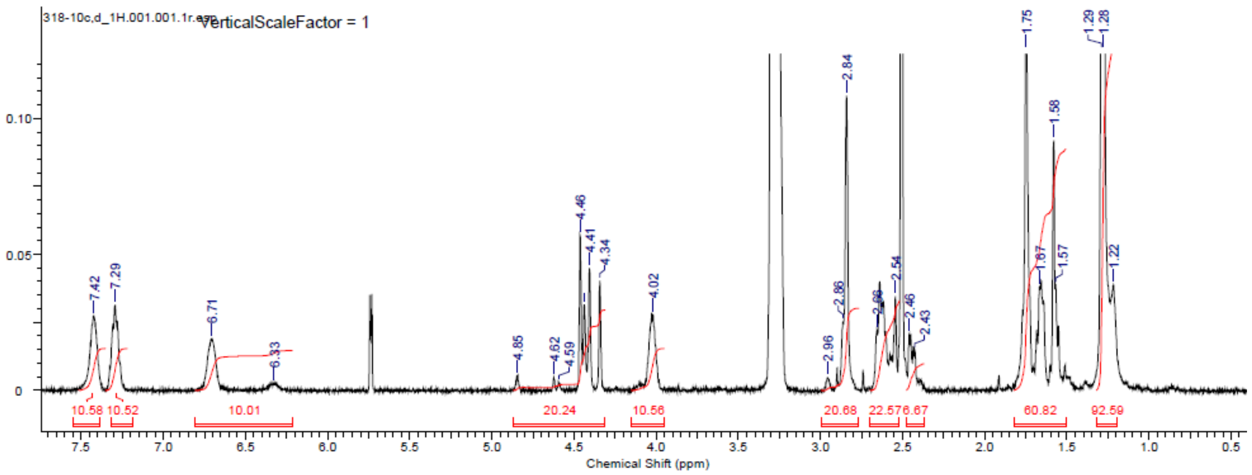

$^{13}C\{^1H\}$ NMR

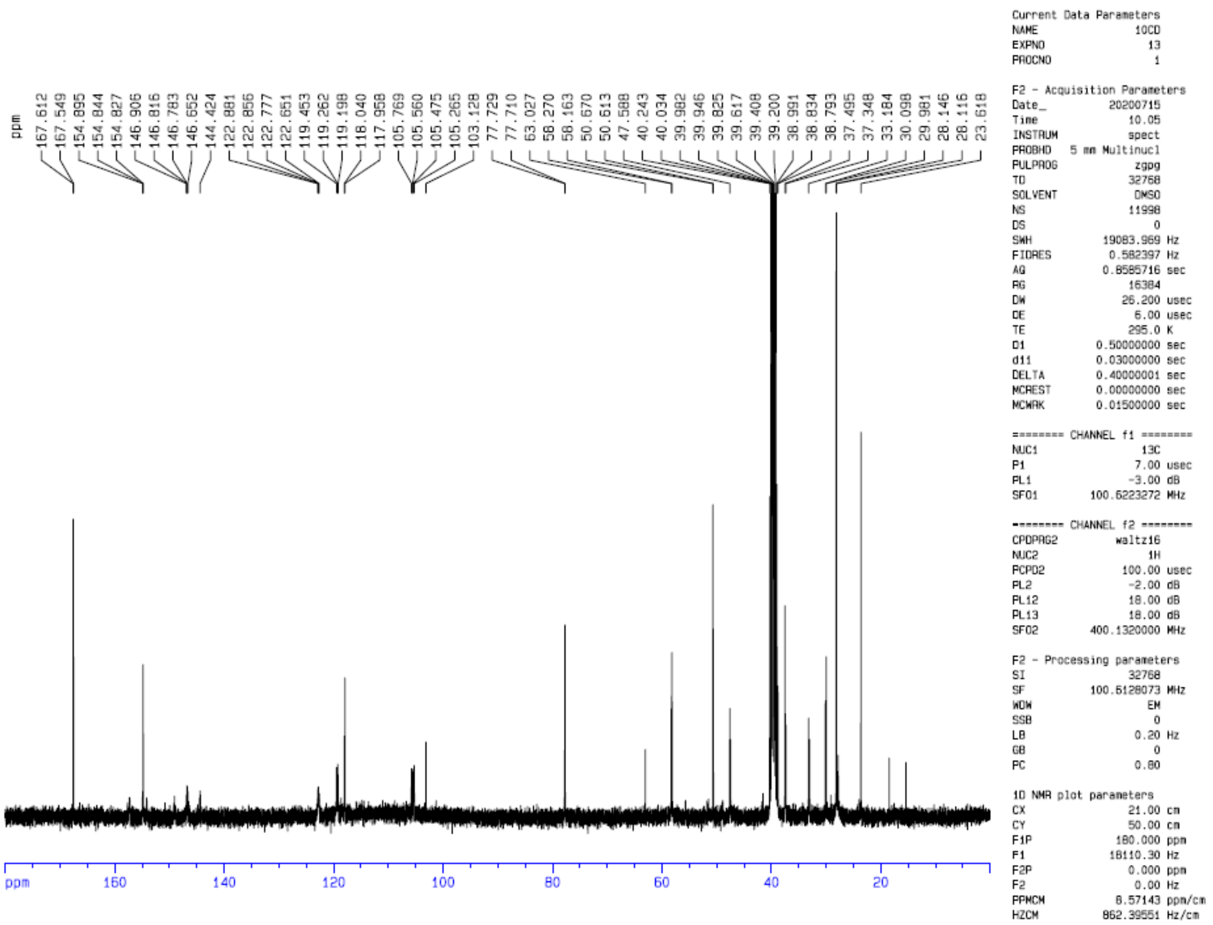

Supplementary material 1

12a,b

^{1}H NMR at RT

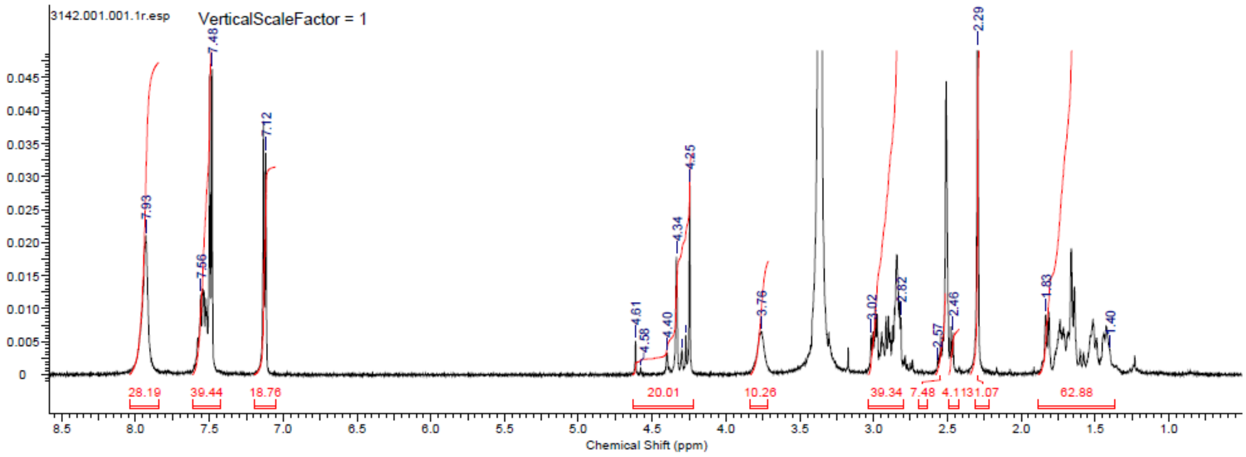

^{1}H NMR registered at different temperatures

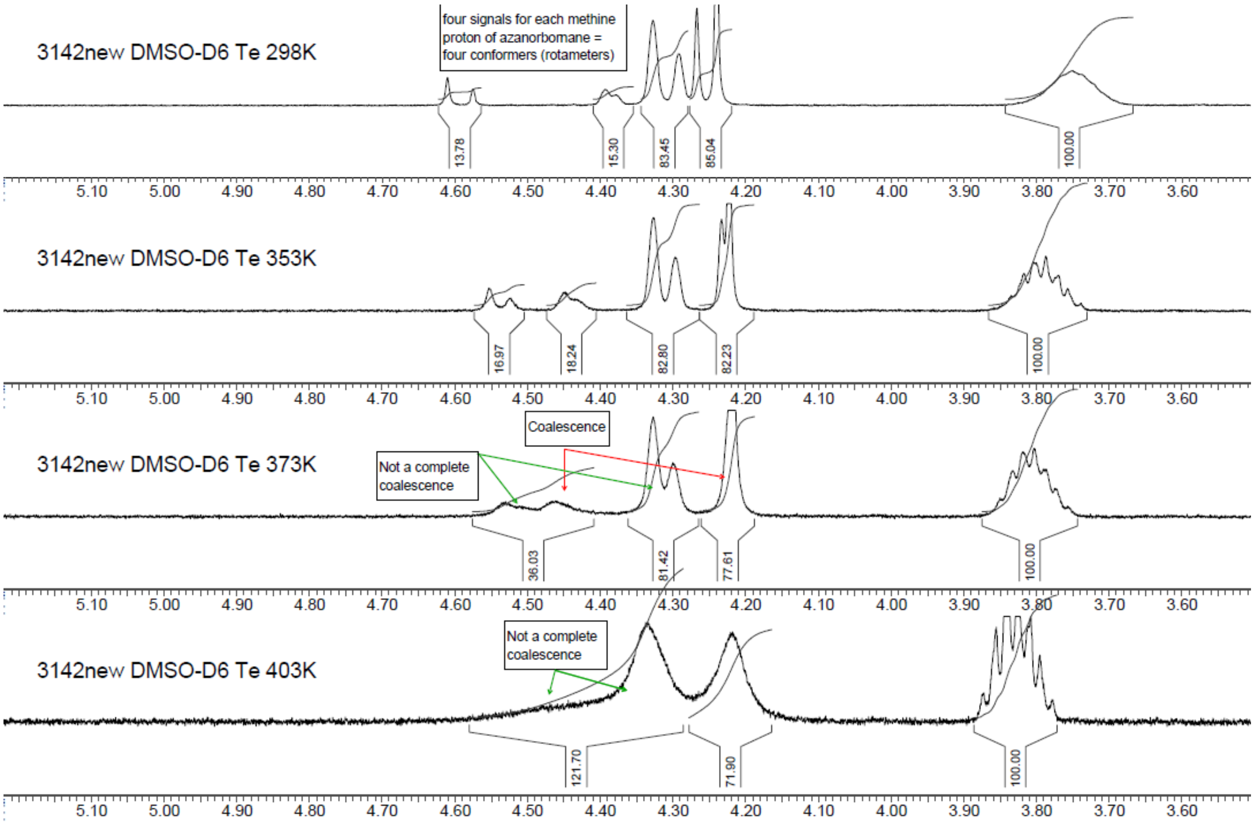

Supplementary material 1

^{1}H NMR registered at temperature 403 K

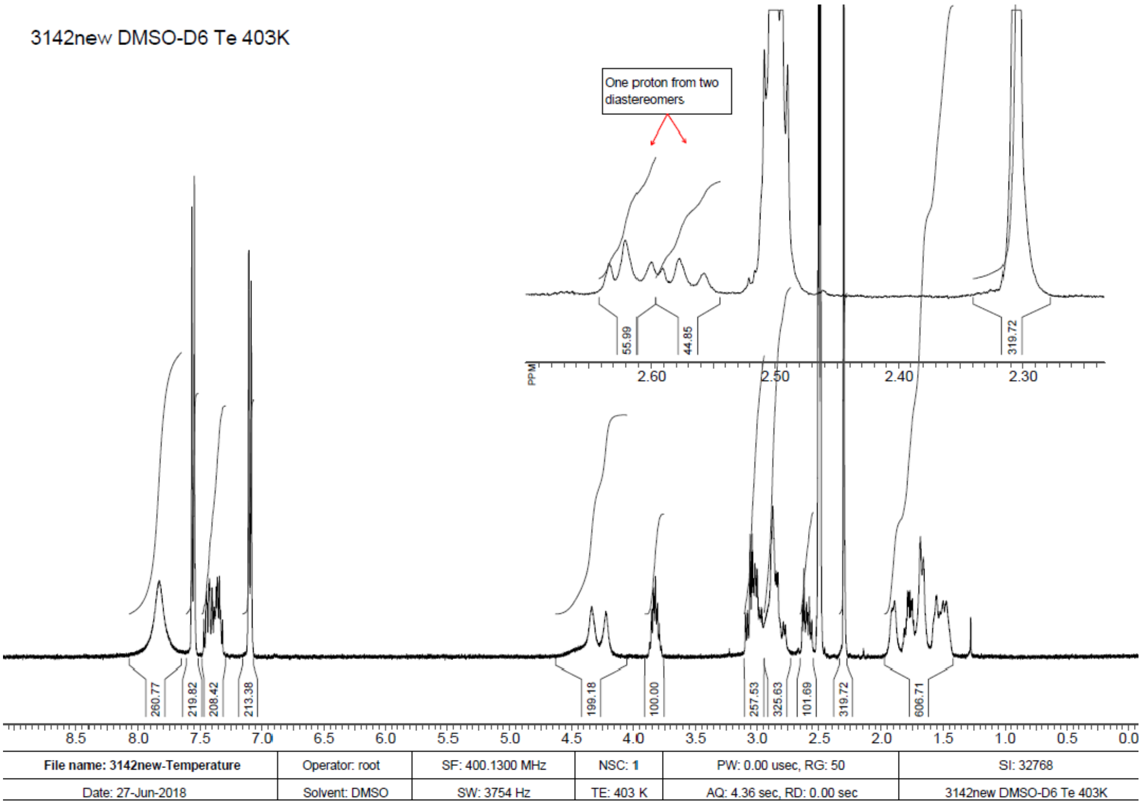

^{19}F NMR

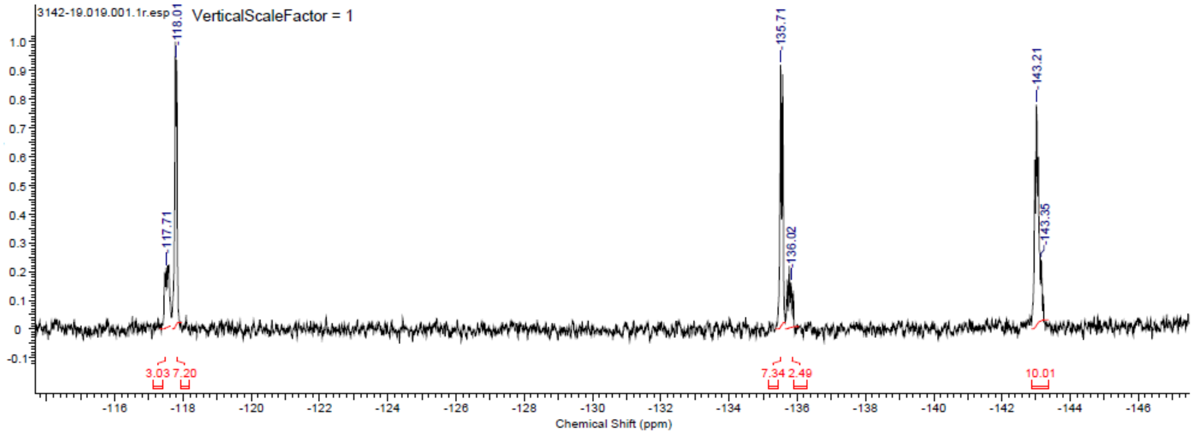

^{13}C{^{1}H} NMR

Supplementary material 1

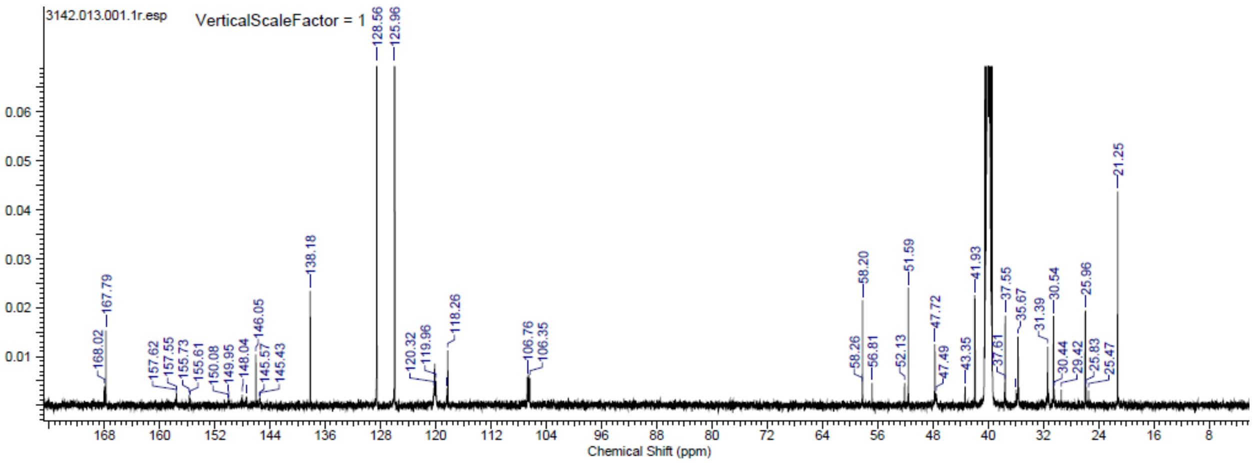

COSY

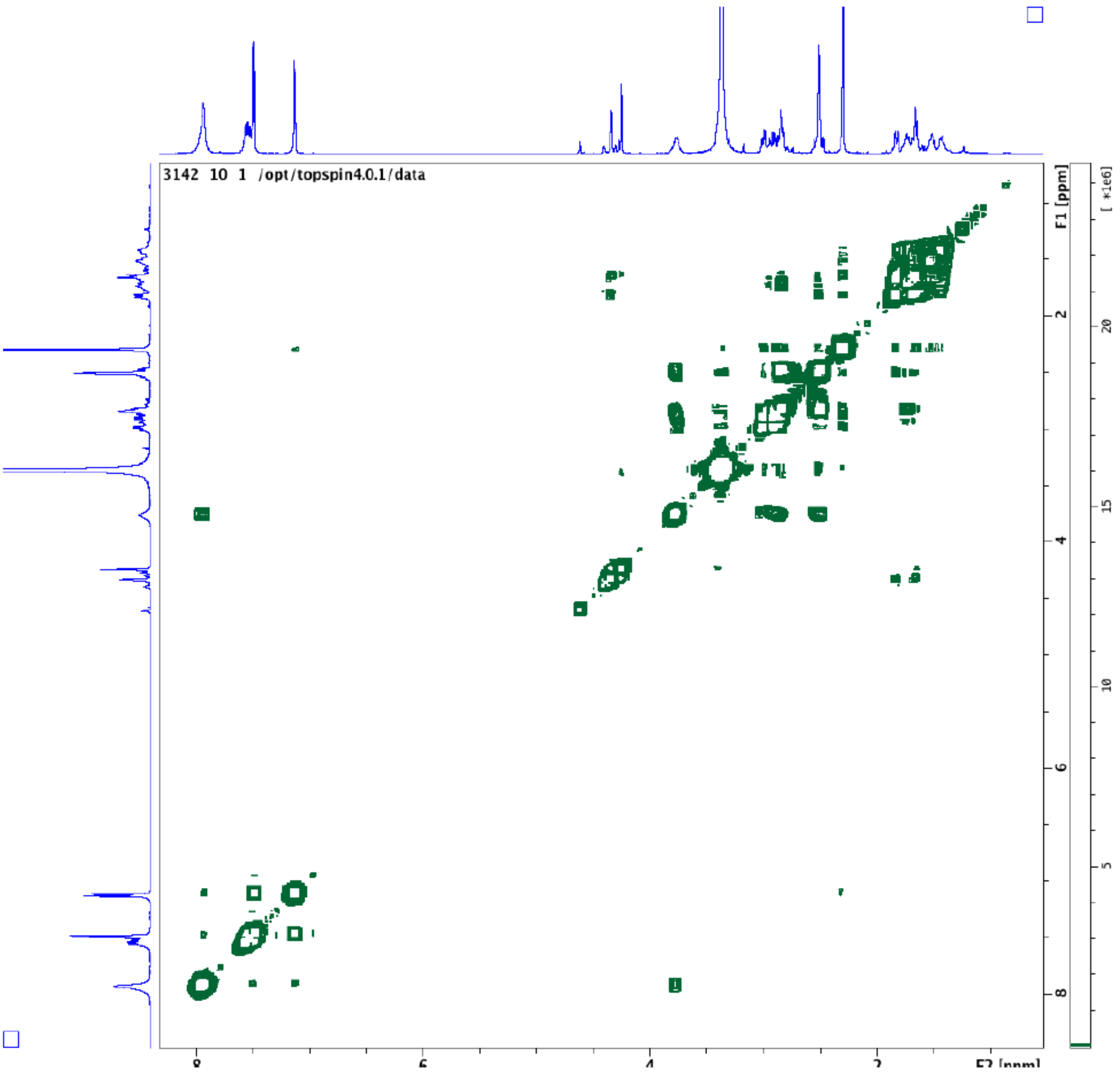

Supplementary material 1

HMBC

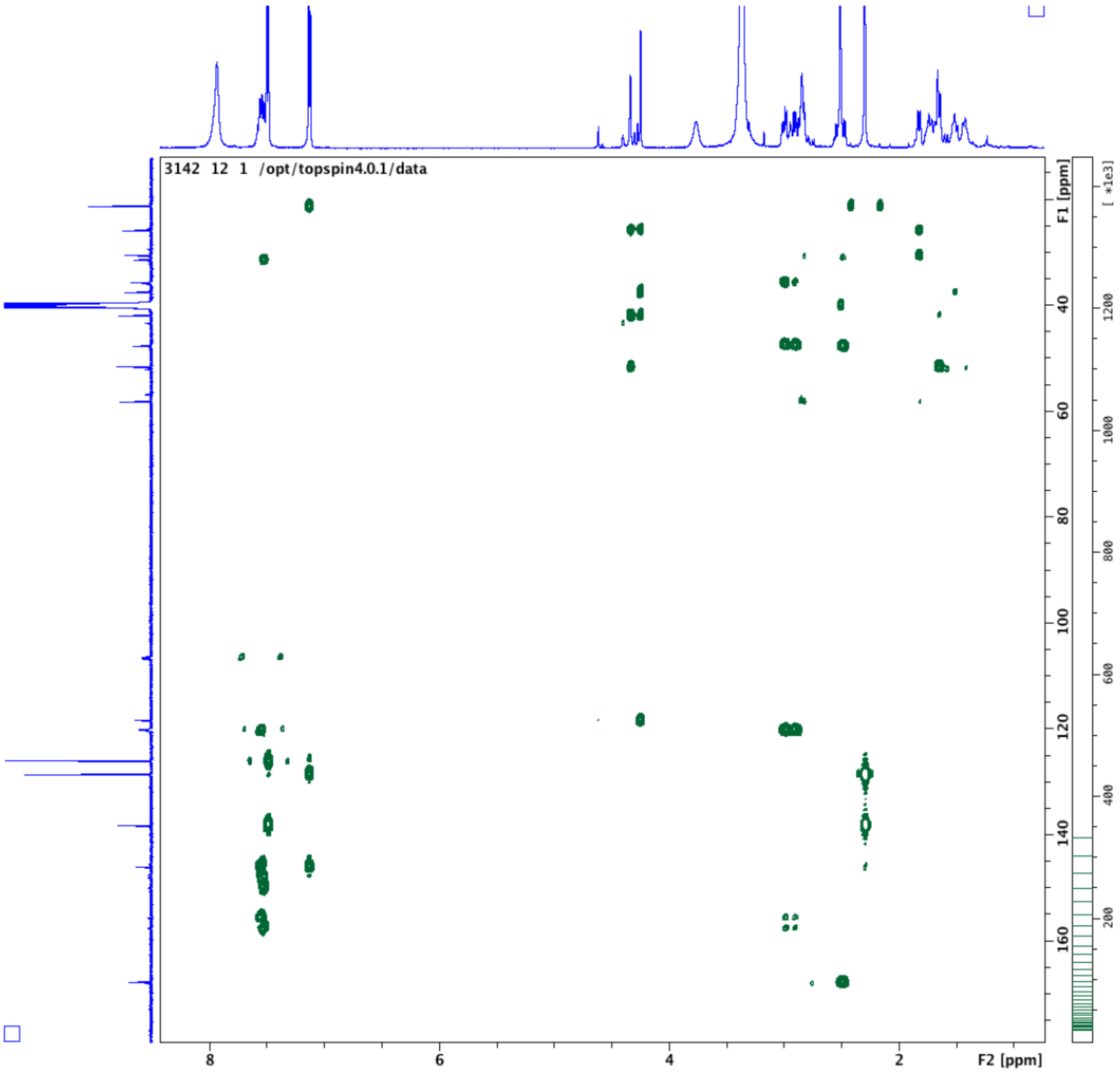

Supplementary material 1

HSQC

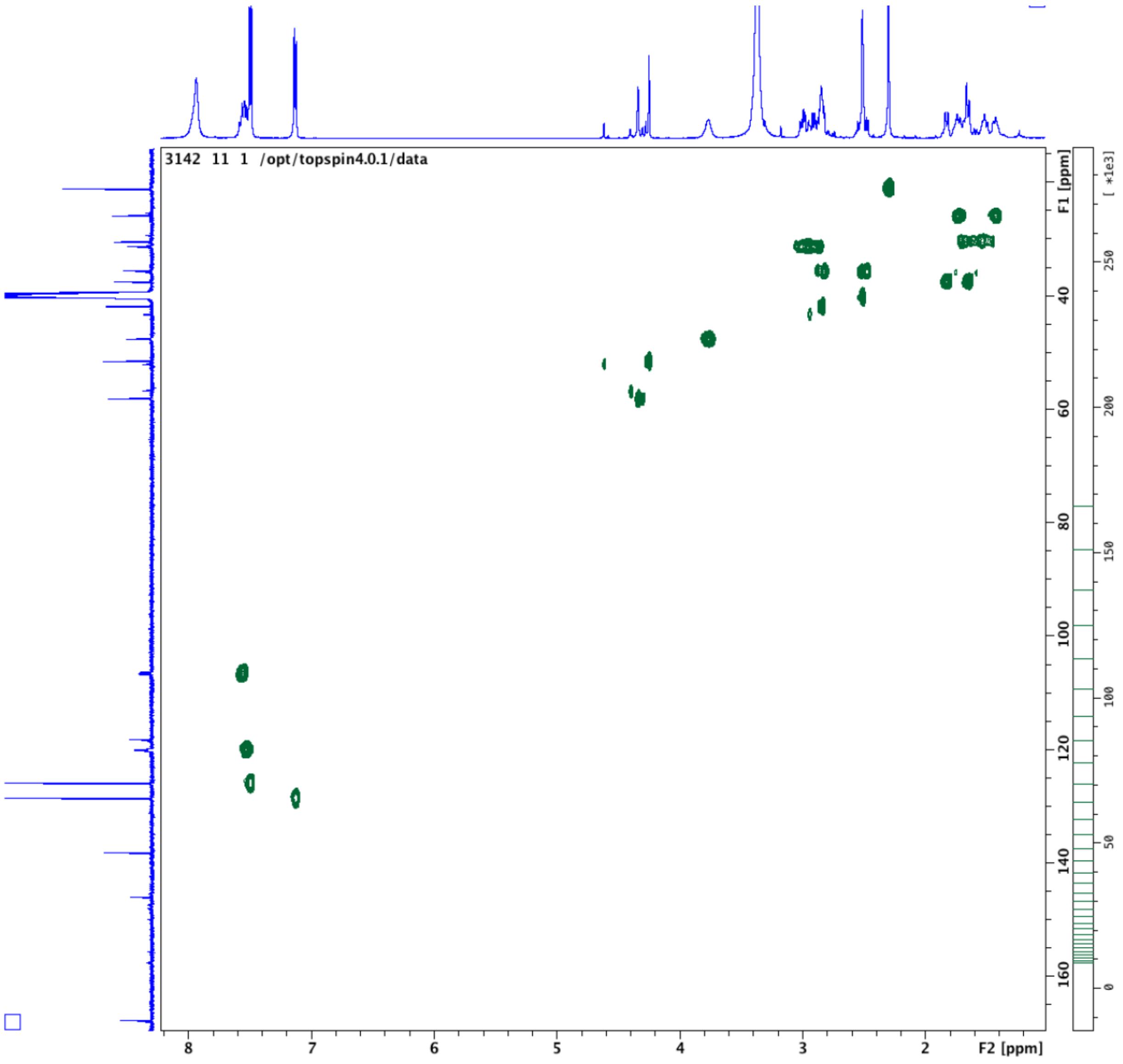

Supplementary material 1

12a

^{1}H NMR at RT

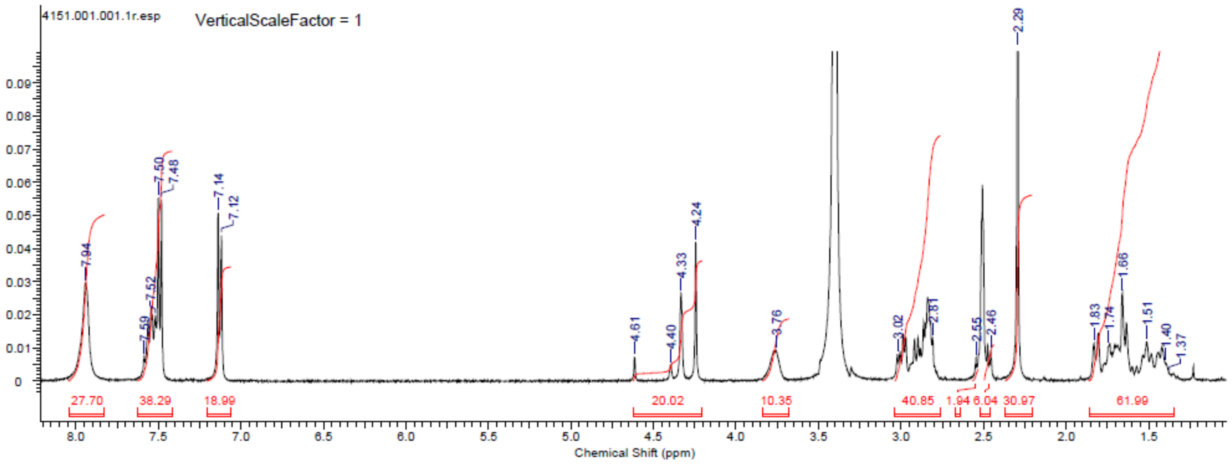

^{1}H NMR at temperature 413 K

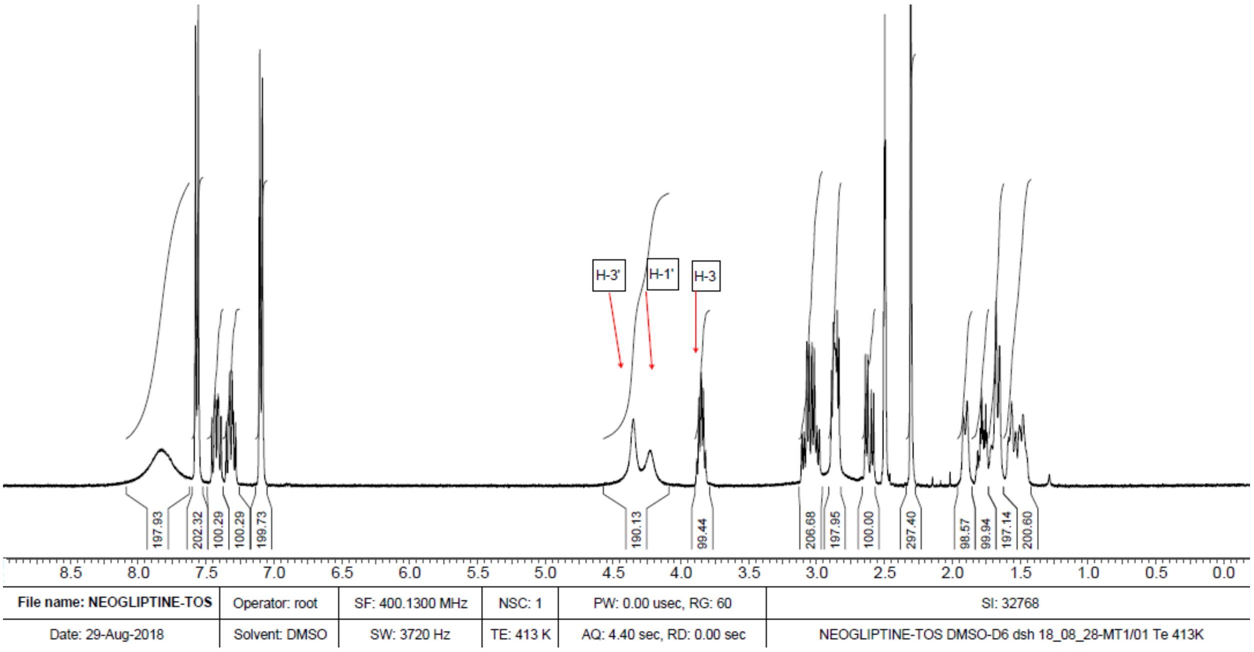

Supplementary material 1

$^{13}C\{^{1}H\}$ NMR

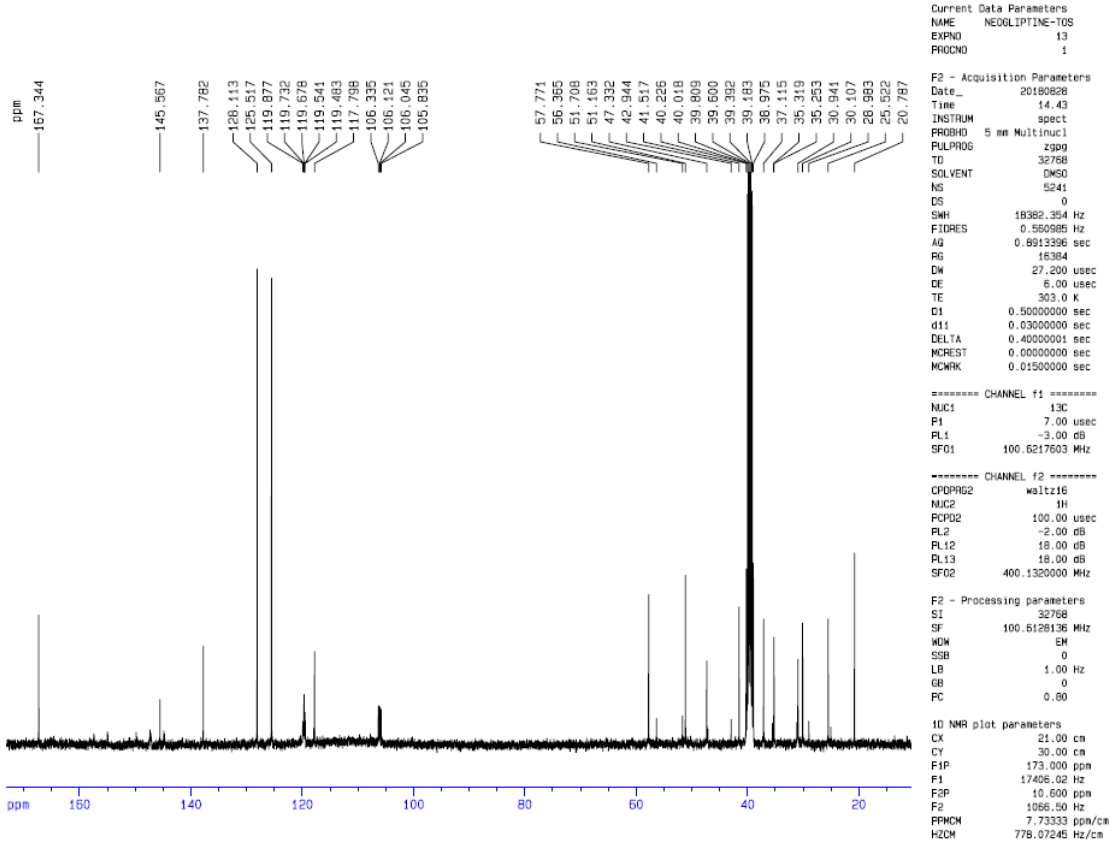

^{13}C apt NMR

Supplementary material 1

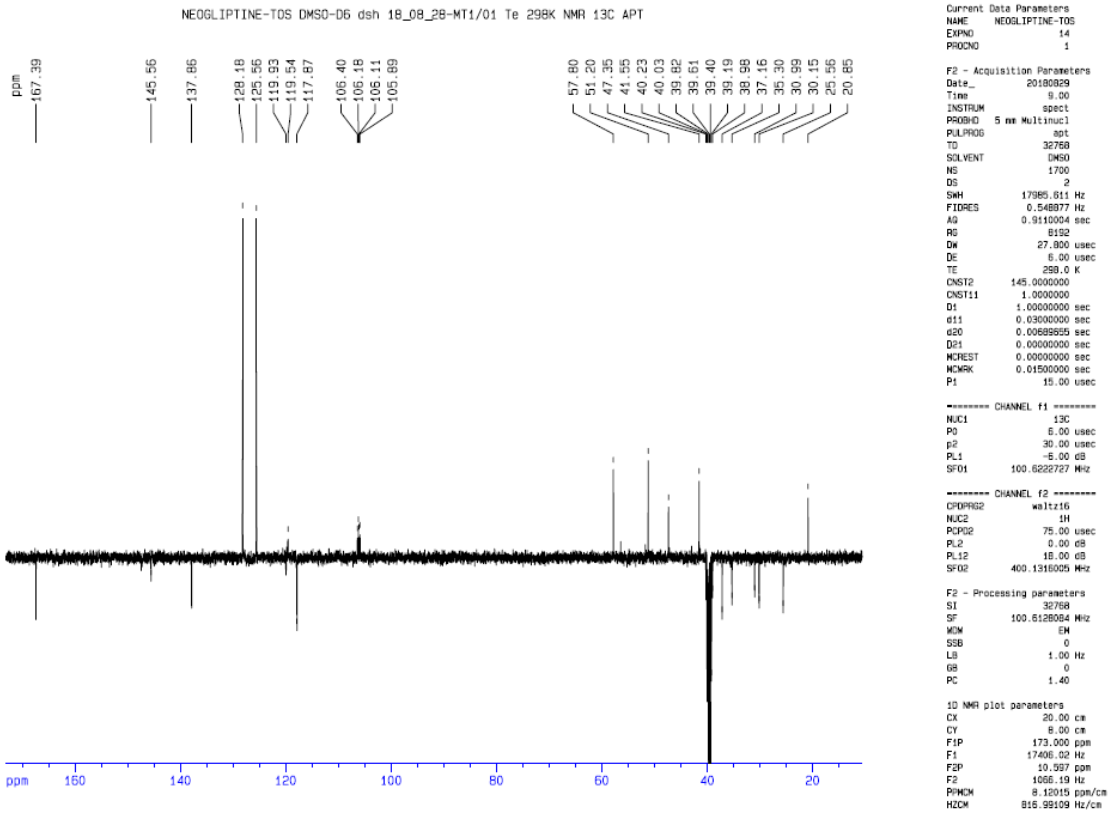

Supplementary material 1

COSY

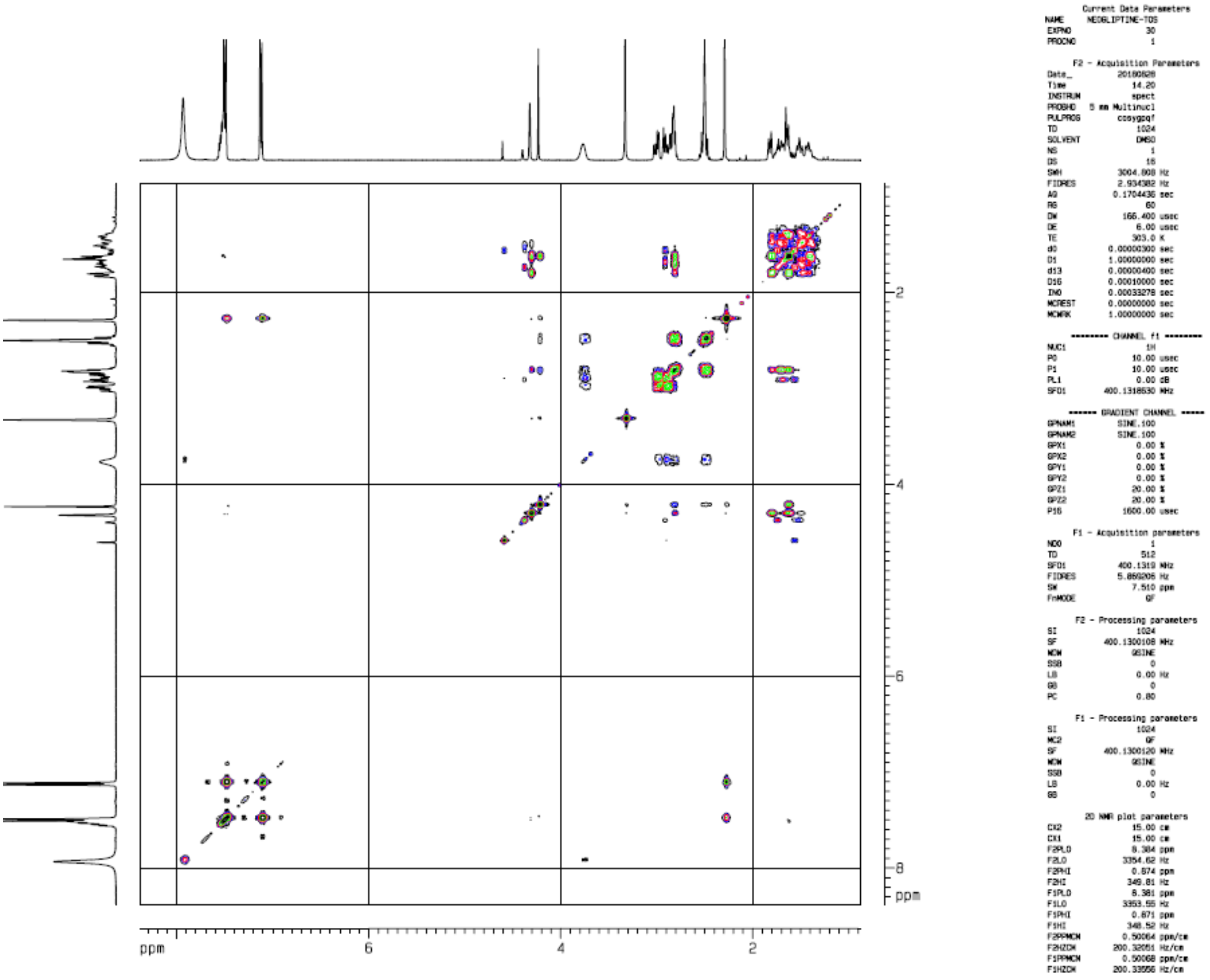

Supplementary material 1

NOESY

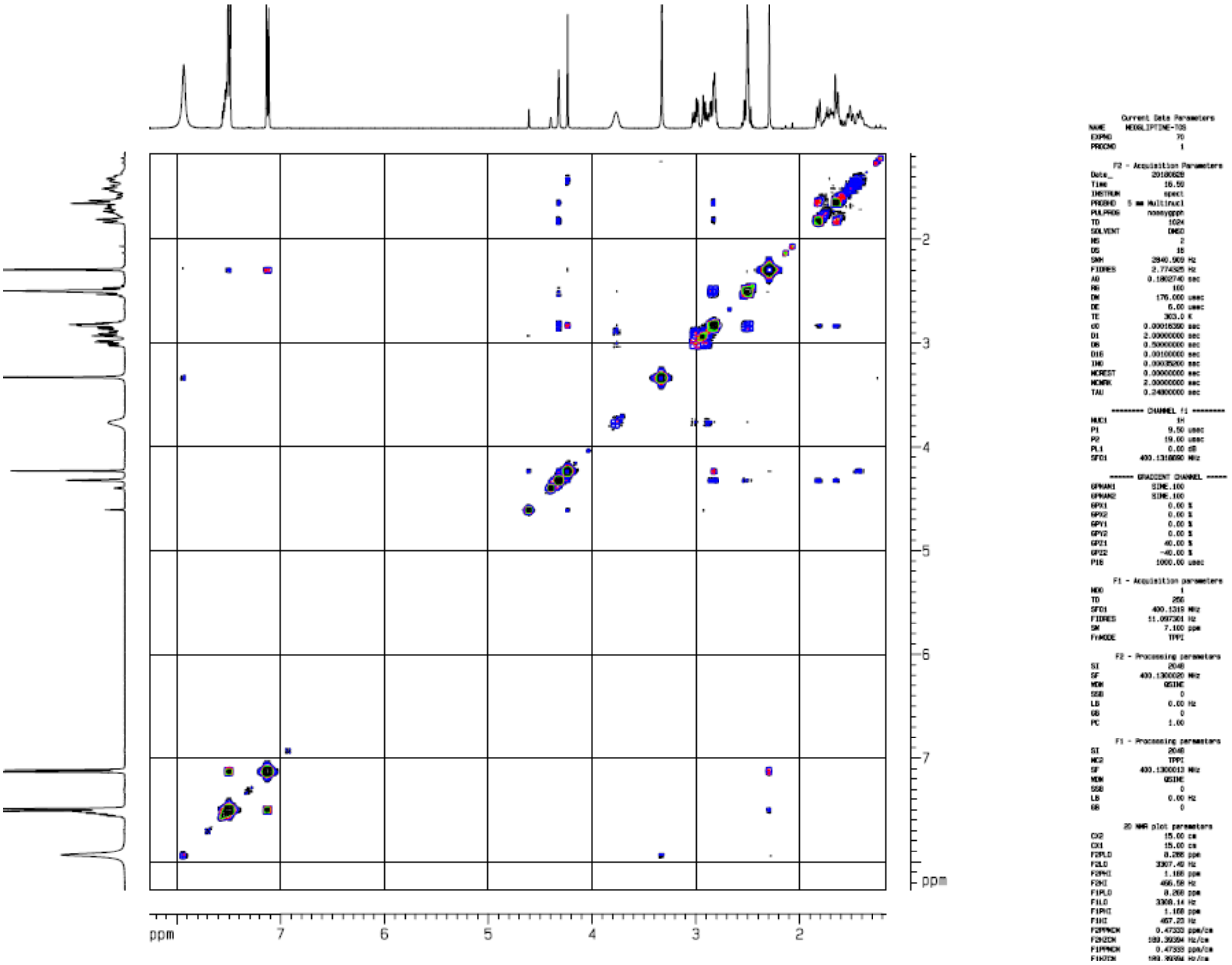

Supplementary material 1

HMBC

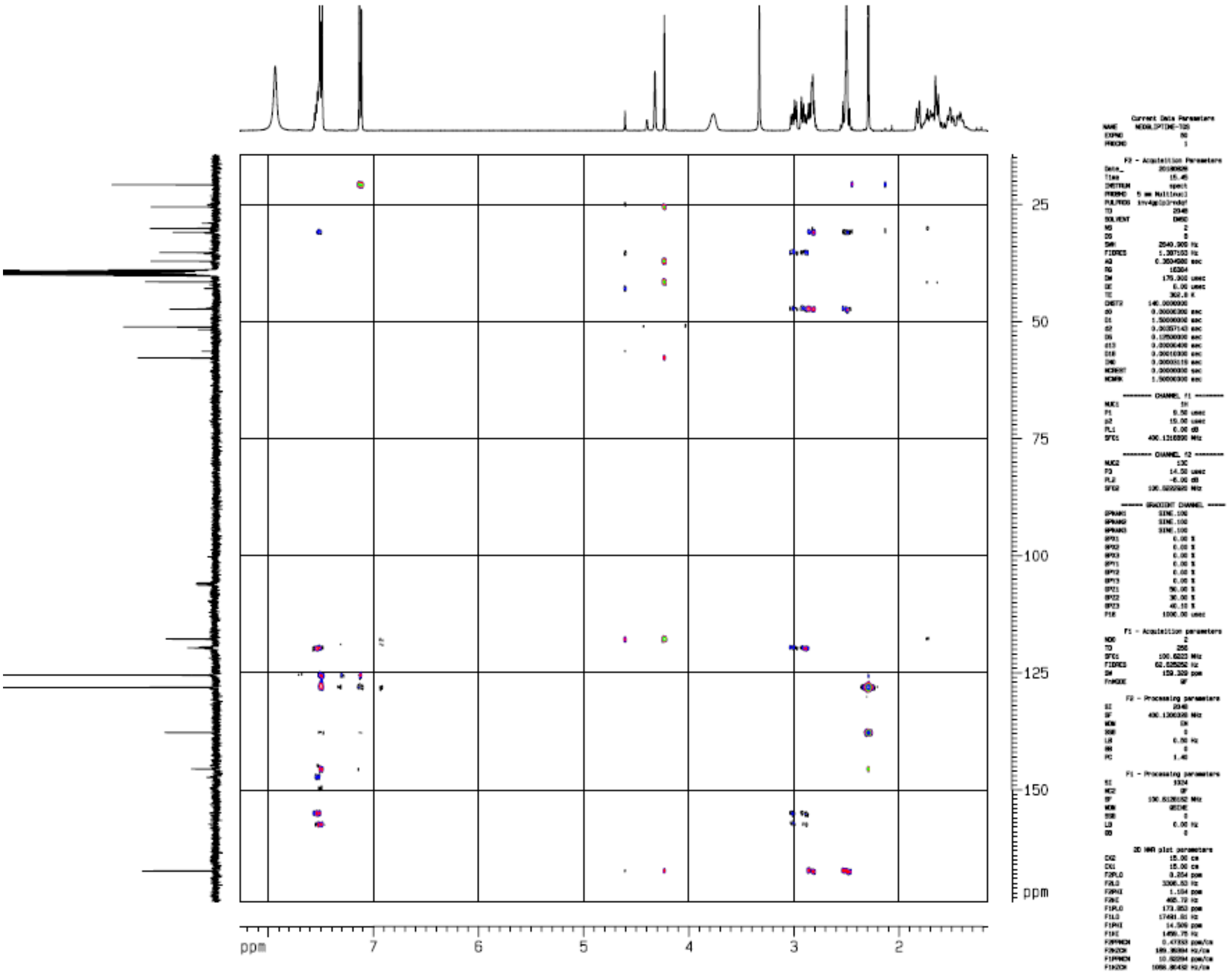

Supplementary material 1

HSCQ

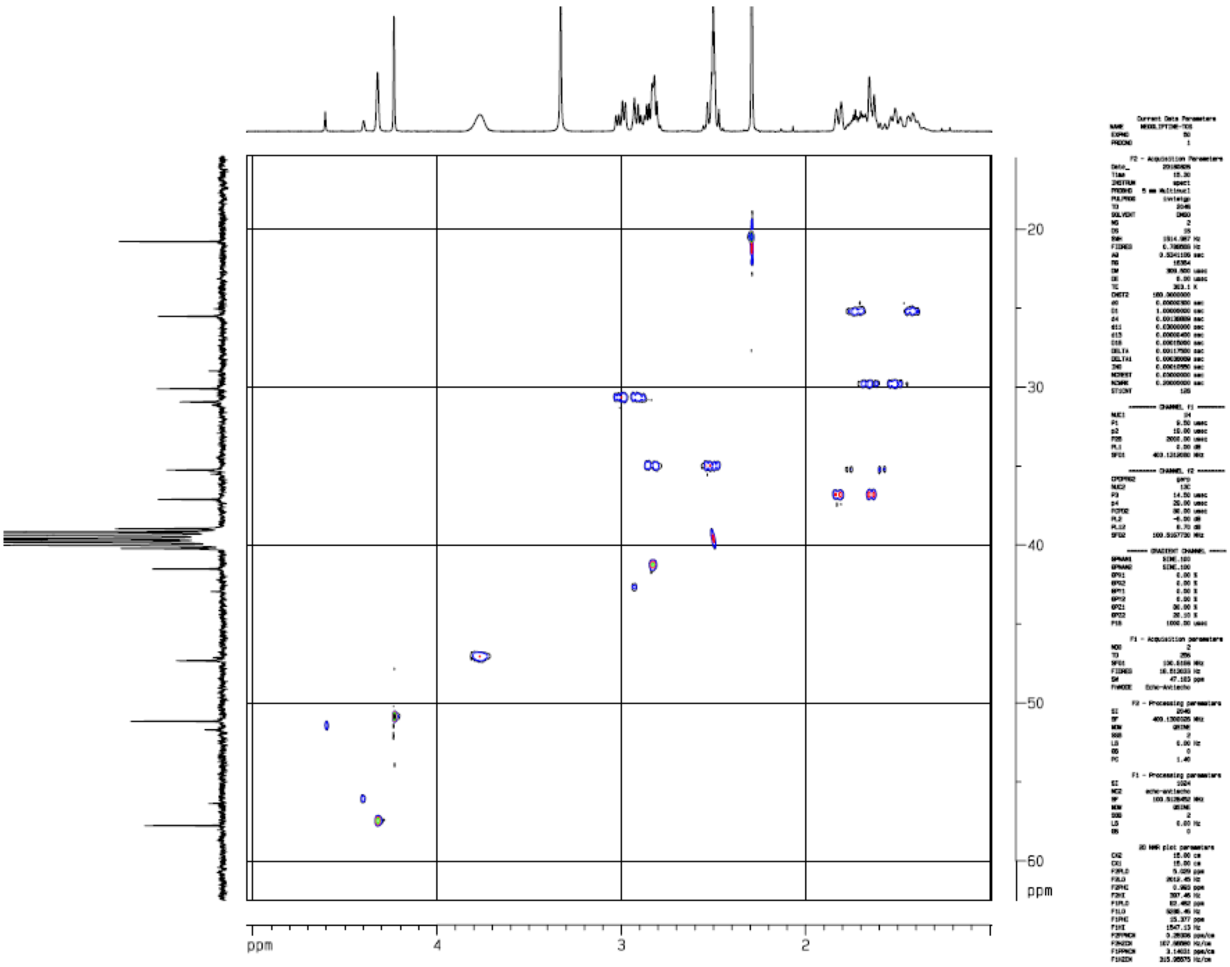

Supplementary material 1

12b

^{1}H NMR at RT

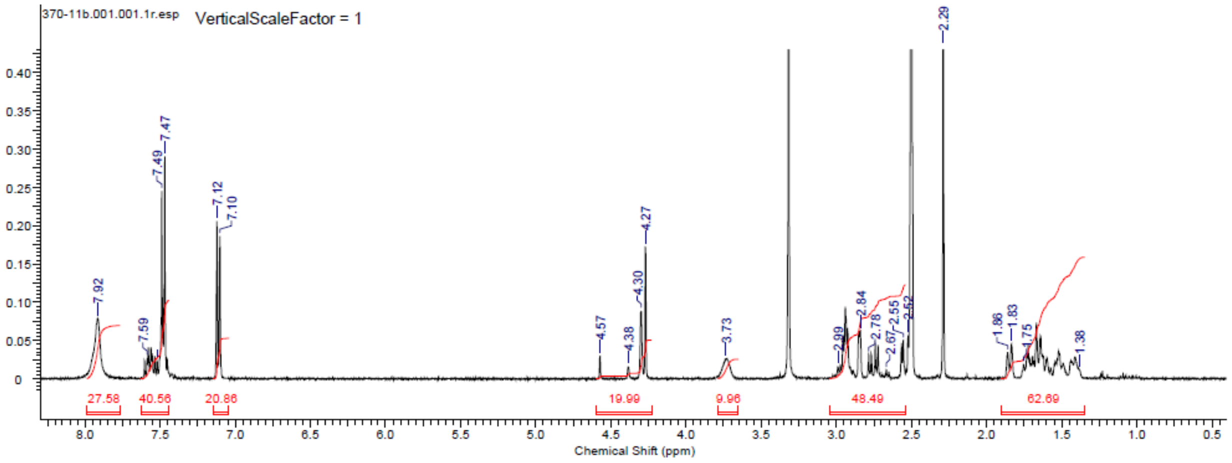

^{1}H NMR at temperature 393 K

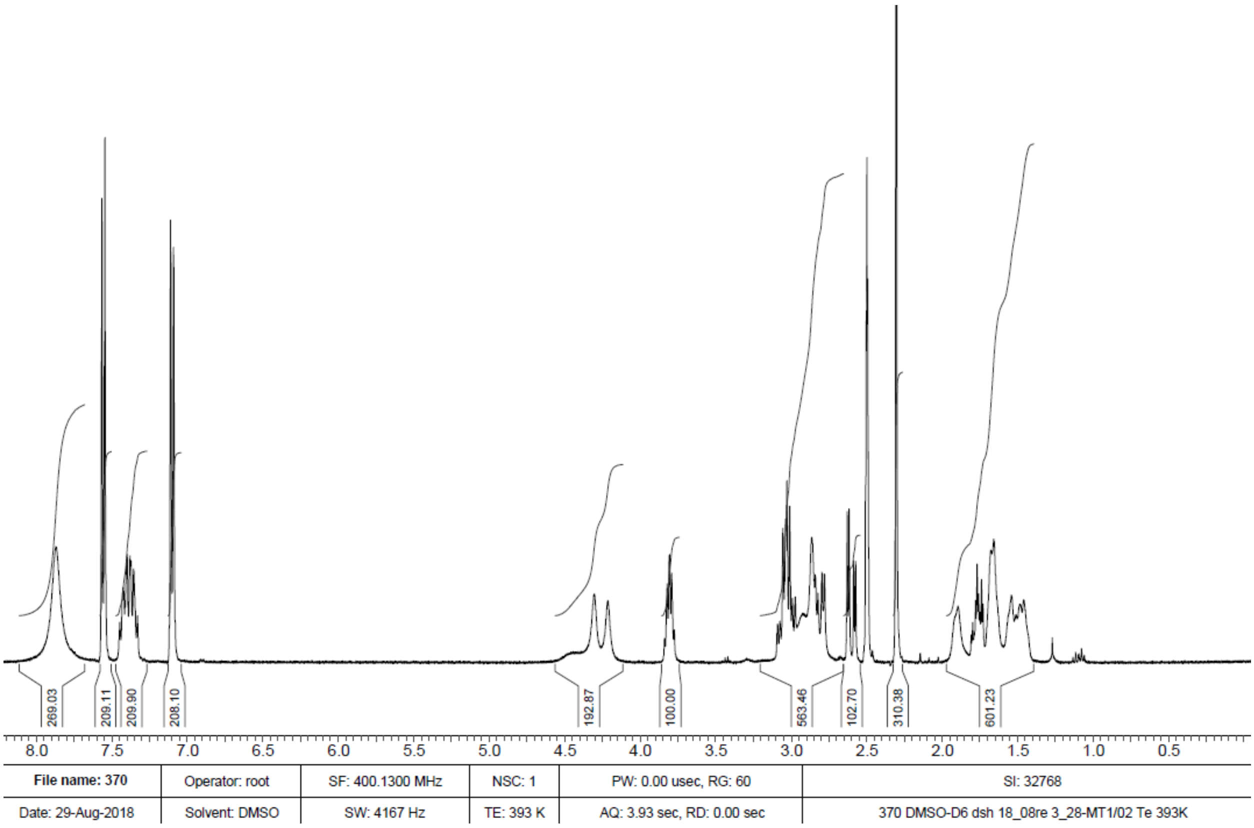

Supplementary material 1

$^{13}C\{^{1}H\}$ NMR

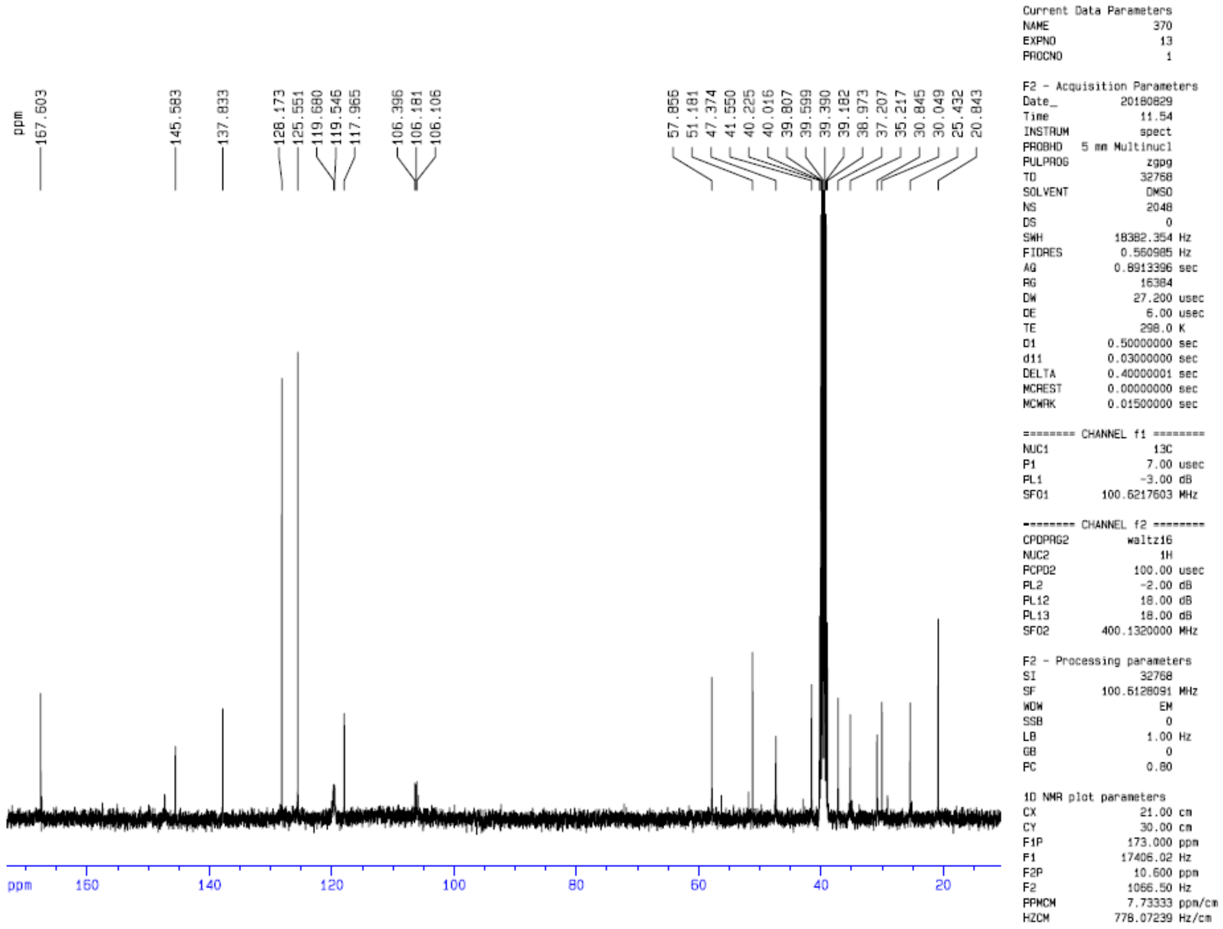

Supplementary material 1

COSY

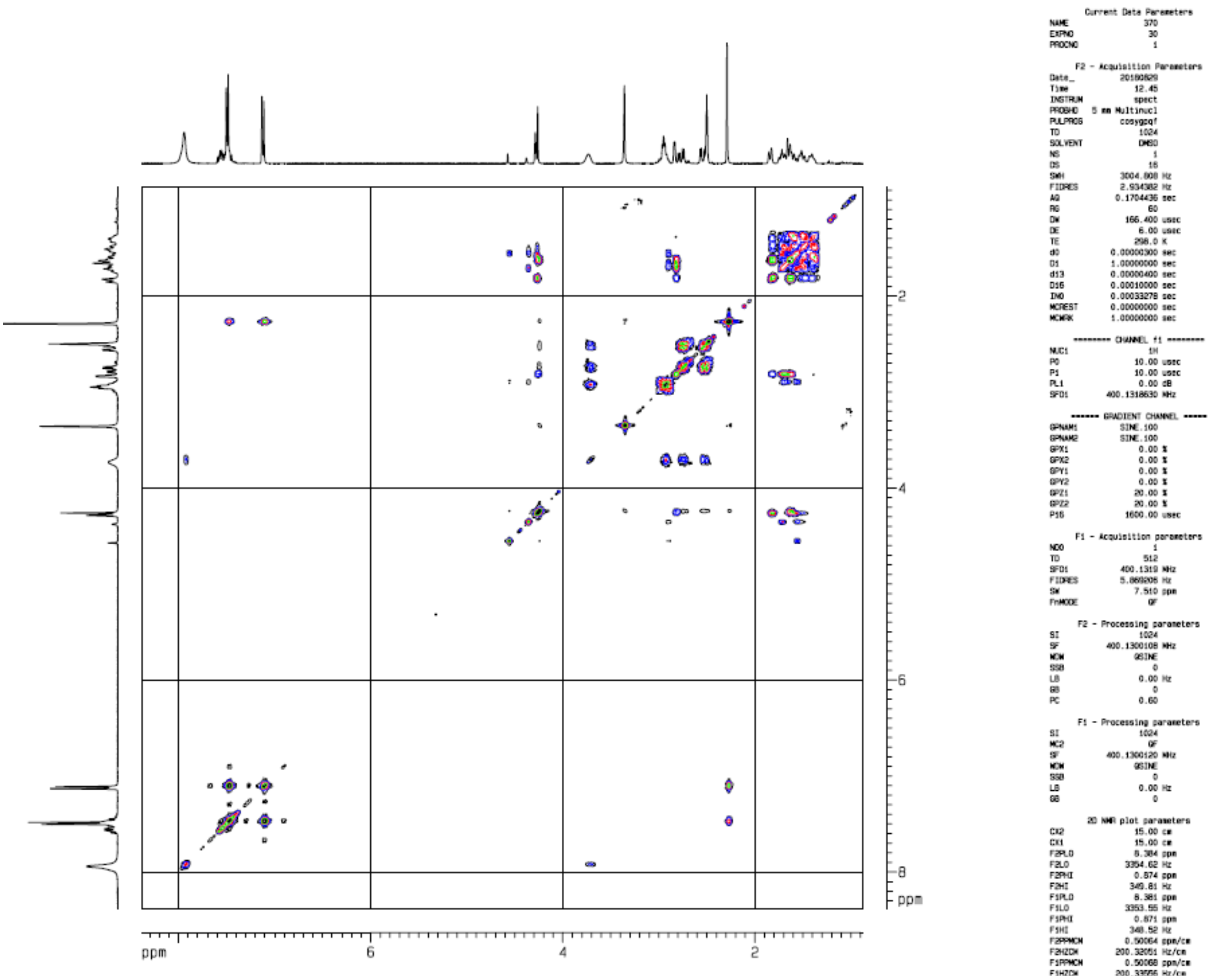

Supplementary material 1

NOESY

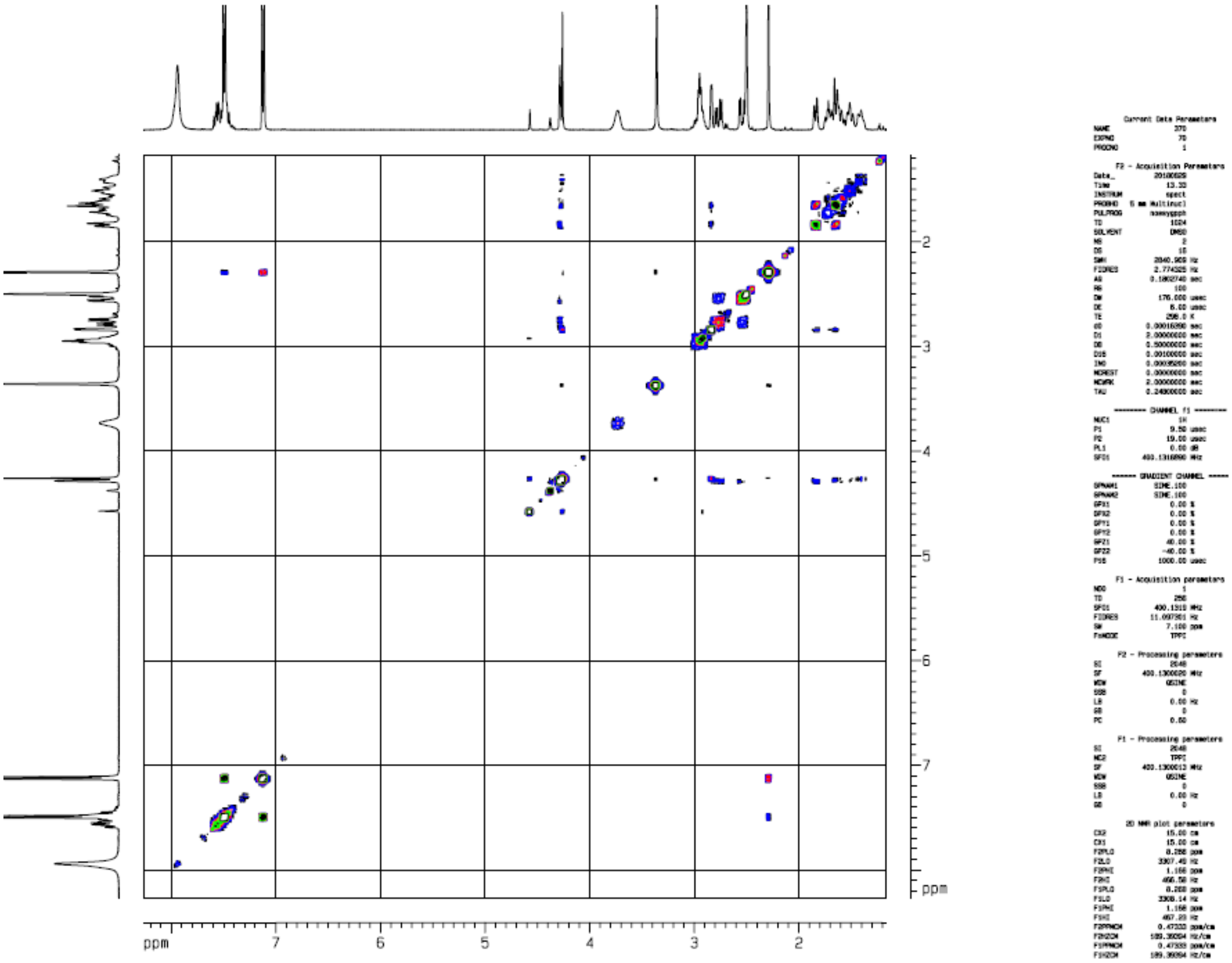

Supplementary material 1

HMBC

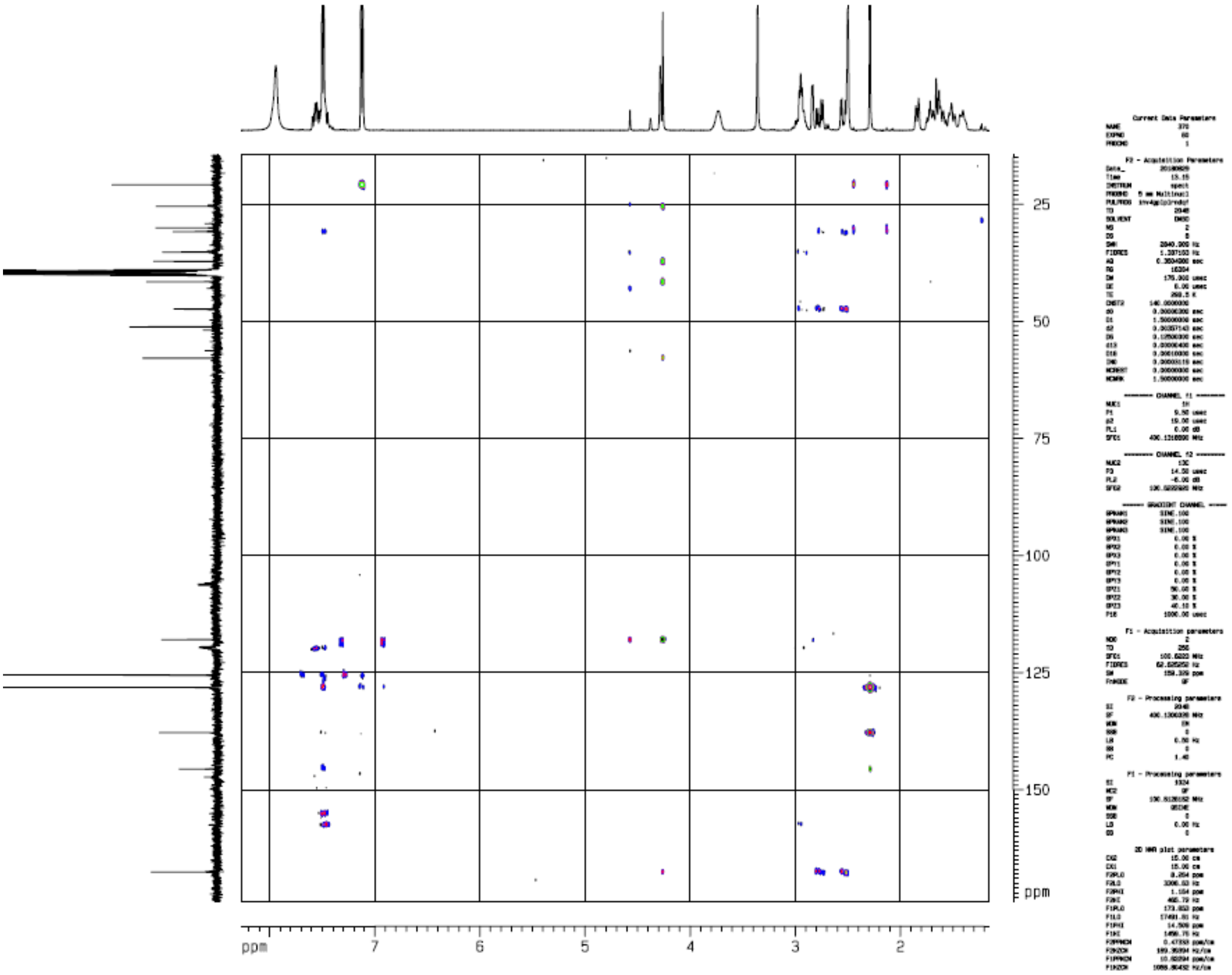

Supplementary material 1

HSQC

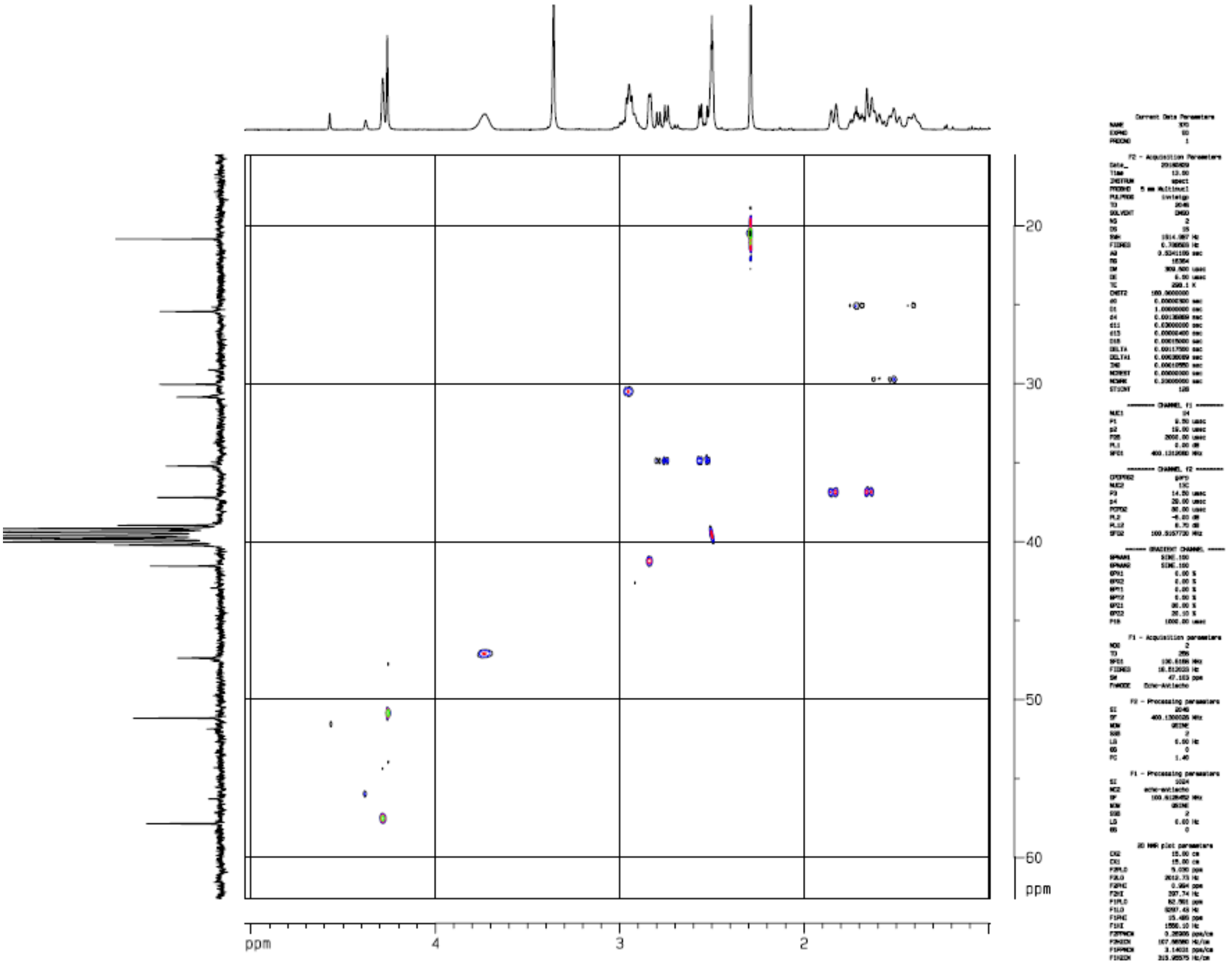

Supplementary material 1

12c,d

^{1}H NMR

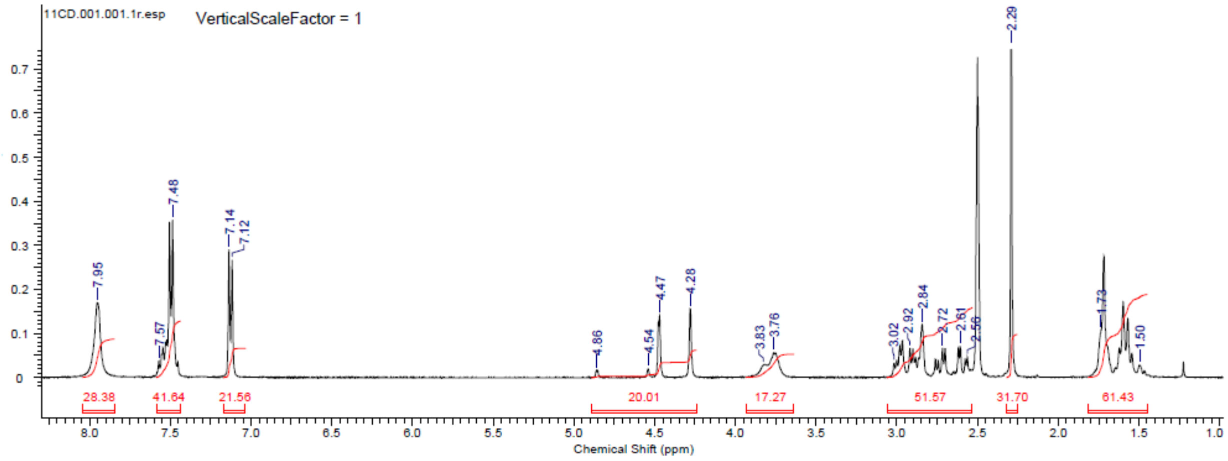

$^{13}C\{^{1}H\}$ NMR

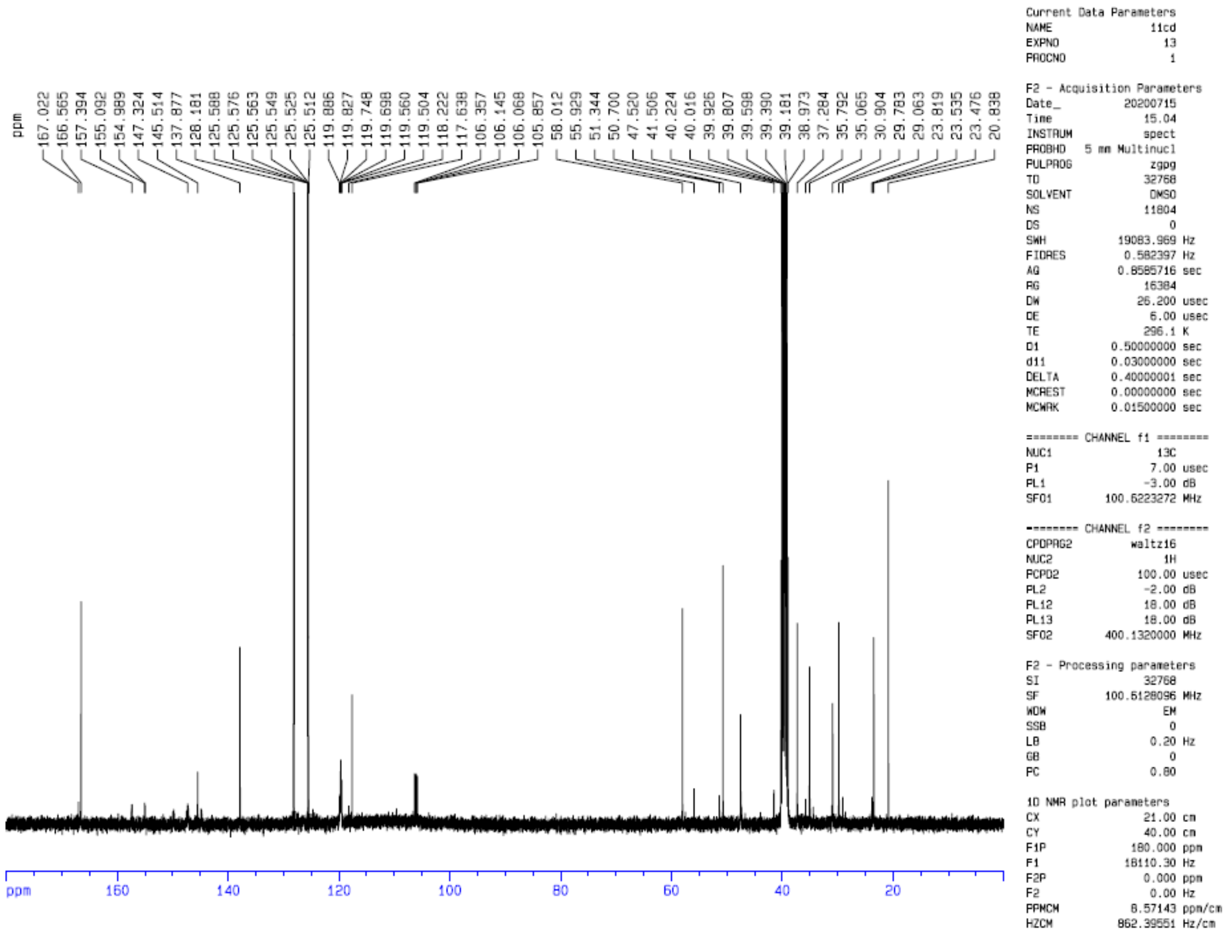

Supplement: Supplementary file 1 [file pharmaceuticals-15-00273-s001.zip › Supplementary_files.zip!/Supplementary file S1_NMR.pdf]
