# Supplementary material for: Design, Synthesis and Biological Evaluation of Neogliptin, a Novel 2-Azabicyclo[2.2.1]heptane-Based Inhibitor of Dipeptidyl Peptidase-4 (DPP-4)
Source: Pharmaceuticals (Basel). 2022 Feb 22;15(3):273. doi: 10.3390/ph15030273 (PMC8949241; doi:10.3390/ph15030273)
Supplement: Supplementary file 1 [file pharmaceuticals-15-00273-s001.zip › Supplementary_files.zip!/Supplementary file S2_mass_spectra.pdf]

**11ab**

```
=====================================================================
Injection Date  : 31/05/2018 16:43:26          Seq. Line :  51
Sample Name     : ZT313                         Location : P1-G-01
Acq. Operator   : 6                                  Inj :   1
Acq. Instrument : Instrument 1                Inj Volume : 3 µl
Different Inj Volume from Sequence !   Actual Inj Volume : 1.5 µl
Method          : C:\HPCHEM\1\METHODS\1PH.M
Last changed    : 25/05/2018 14:12:20  by 6
Column: Onyx Monolithic C18 50x4.6mm | 3.75ml/min | Columns Reg Valve
Gradient: "A"->@2.2min->"B"(Hold 0.4min)->@0.2min->"A"->PostRun
=====================================================================

Instrument Conditions    :    At Start           At Stop
Pressure                 :     100.5              62.7   bar
Flow                     :       3.750             3.750 ml/min

Detector Lamp Burn Times: Current On-Time  Accumulated On-Time
DAD 1, UV Lamp           :      29.75           59382.8   h
DAD 1, Visible Lamp      :      OFF             13046.5   h

Solvent Description      :
PMP1, Solvent A          : 0.1%TFA in Acn/H2O (2.5:97.5)
PMP1, Solvent B          : 0.1%TFA in AcN
PMP1, Solvent C          : 0.1%FA in Acn/H2O (2.5:97.5)
PMP1, Solvent D          : 0.1%FA in AcN
=====================================================================
MSD parameters
Tune file name               :    C:\HPCHEM\1\1956ATUN\atunes.tun
Ionization mode              :    APCI

MSD Instrument Conditions    :      At Start        At Stop
Quad Temp                    :            99             99 C
Gas Temp                     :           350            350 C
Vaporizer                    :           351            356 C
RoughVac                     :             2              2 Torr
HighVac                      :      8.3E-006       8.3E-006 Torr
CapCur                       :            94            925 nA
ChamCur                      :             3              0 µA
CoronaVol                    :          4353             39 Volt
DryingGas                    :            10             10 l/min
Neb Pres                     :            50             50 psig
Turbo1Spd                    :            99             99 %
Turbo1Pwr                    :            98             98 W
RF Drive                     :      0.0E-001       0.0E-001 %
Qd TpDrv                     :             8              6 %
Gas TpDrv                    :            28             29 %
Vap TpDrv                    :            27             27 %
Neb PrDrv                    :            43             44 %
Gas FlDrv                    :             6              6 %
DelaySens                    :      0.0E-001       0.0E-001 V
Aux Input                    :      0.0E-001       0.0E-001 V
Other Det                    :      0.0E-001       0.0E-001 V
=====================================================================
MSD tuning (calibration) parameters

Ionization polarity          :    Positive
Skim1                        :    Not Applicable
Skim2                        :    8.0 V
Ion Energy                   :    5.0 V
Lens1                        :    3.3 V
Lens2                        :    29 V
Iris                         :    -75 V
HED                          :    10000 V
Width Gain                   :    322
Width Offset                 :    Variable
                                  Mass         :   Value
                                  --------------------------
                                  121.05       :   -40
                                  622.03       :   -15
                                  922.01       :   -40
                                  --------------------------
Mass Gain                    :    30.25
Mass Offset                  :    0.654
Quad DC                      :    0.00 V
Octopole Peak                :    650 V
```

| | | |
|---|---|---|
| Octopole Knee | : | Not Applicable |
| Lens2DC | : | Not Applicable |
| L2RFEn | : | Not Applicable |
| L2RFPh | : | Not Applicable |
| L2RFAmp | : | Not Applicable |
| Mass Filter | : | Gaussian |
| Mass Filter Width | : | 0.30 Da |
| Time Filter | : | Gaussian |
| Time Filter Width | : | 0.030 minutes |

=====================================================================

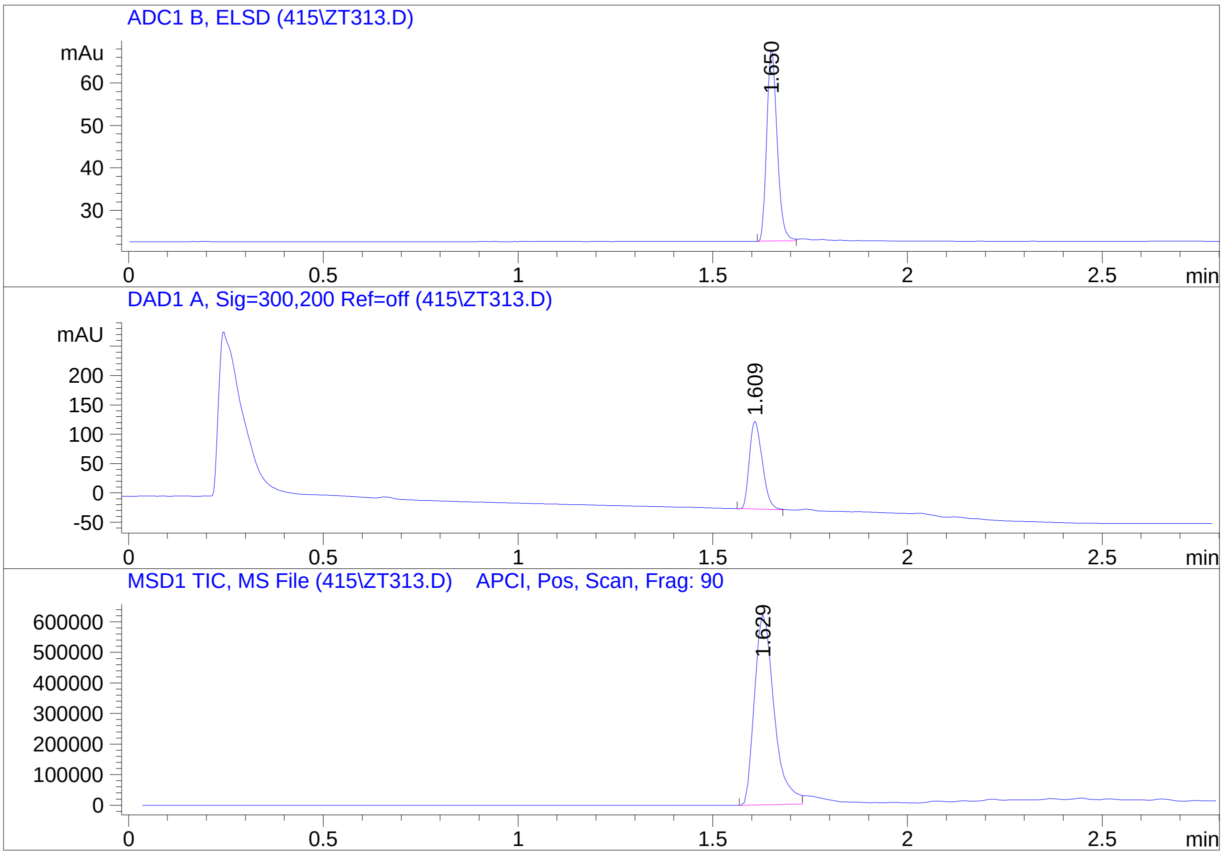


=====================================================================

Area Percent Report

=====================================================================

| | | |
|---|---|---|
| Sorted By | : | Signal |
| Multiplier | : | 1.0000 |
| Dilution | : | 1.0000 |

Use Multiplier & Dilution Factor with ISTDs

Signal 1: ADC1 B, ELSD

| Peak # | RetTime [min] | Type | Width [min] | Area [mAu*s] | Height [mAu] | Area % |
|---|---|---|---|---|---|---|
| 1 | 1.650 | PB | 0.0298 | 84.29549 | 45.14272 | 100.0000 |

Totals : 84.29549 45.14272

Signal 2: DAD1 A, Sig=300,200 Ref=off

| Peak # | RetTime [min] | Type | Width [min] | Area [mAU*s] | Height [mAU] | Area % |
|---|---|---|---|---|---|---|
| 1 | 1.609 | BB | 0.0370 | 346.19089 | 149.37743 | 100.0000 |

Totals : 346.19089 149.37743

Signal 3: MSD1 TIC, MS File

| Peak # | RetTime [min] | Type | Width [min] | Area | Height | Area % |
|---|---|---|---|---|---|---|
| 1 | 1.629 | PB | 0.0547 | 2.18808e6 | 6.27098e5 | 100.0000 |

Totals : 2.18808e6 6.27098e5

================================================================

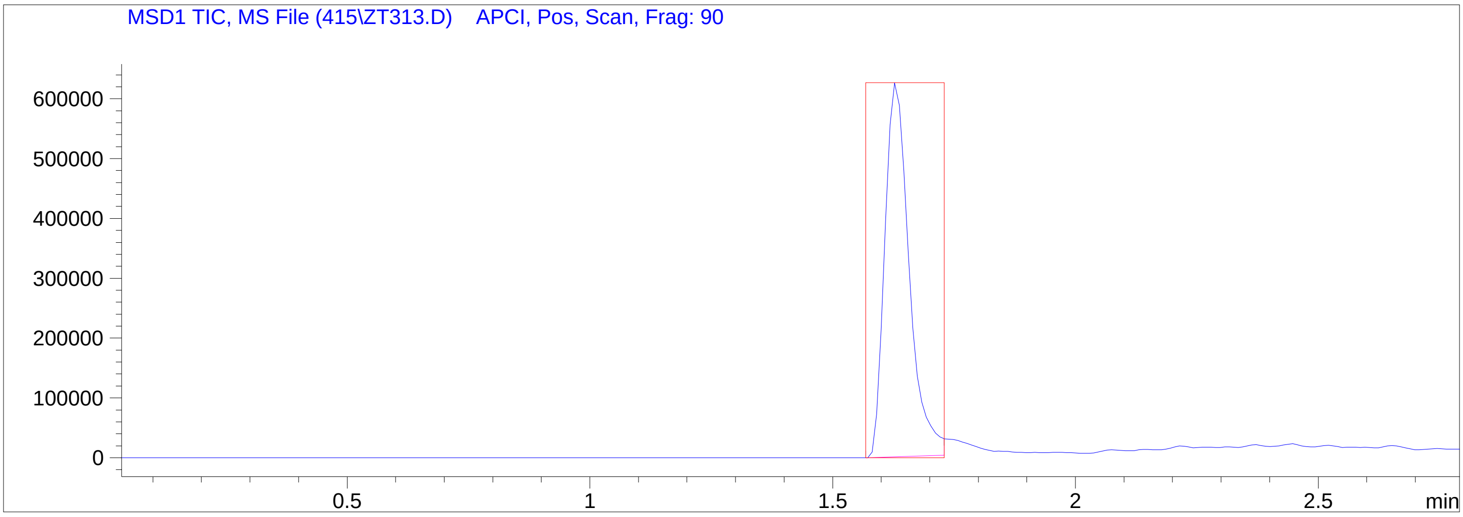


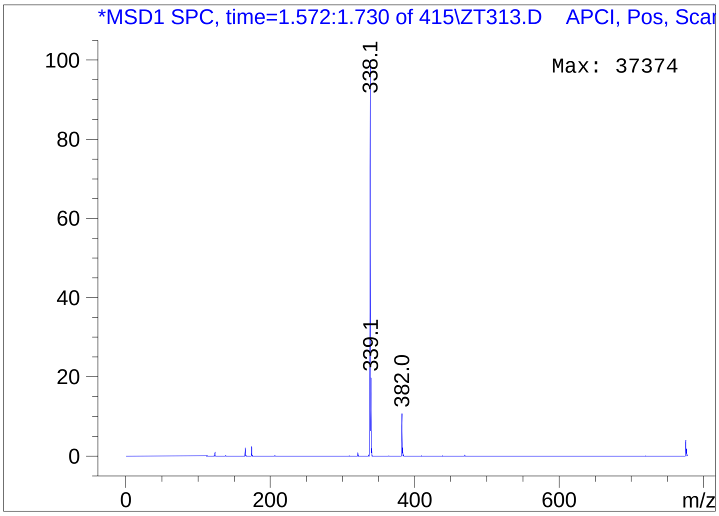


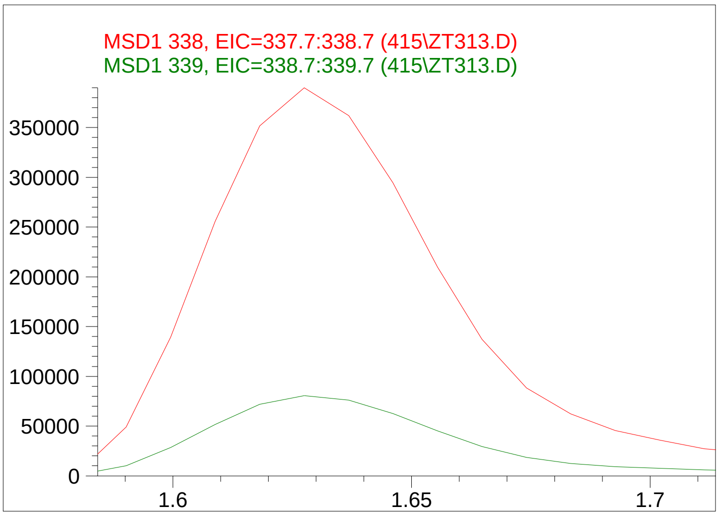


Peak #1 at 1.629 min ( 1.568 to 1.730 min)
-> The analysis found only one component, indicating a pure peak. <-

Component 1: Peak at Scan 172.1. Top ions are 338 339 382

---

*** End of Report ***

**11cd**

```
======================================================================
Injection Date  : 31/05/2018 16:59:53           Seq. Line :  55
Sample Name     : ZT318                          Location : P1-C-02
Acq. Operator   : 6                                   Inj :   1
Acq. Instrument : Instrument 1                 Inj Volume : 3 µl
Different Inj Volume from Sequence !    Actual Inj Volume : 1.5 µl
Method          : C:\HPCHEM\1\METHODS\1PH.M
Last changed    : 25/05/2018 14:12:20  by 6
Column: Onyx Monolithic C18 50x4.6mm | 3.75ml/min | Columns Reg Valve
Gradient: "A"->@2.2min->"B"(Hold 0.4min)->@0.2min->"A"->PostRun
======================================================================

Instrument Conditions    :    At Start           At Stop
Pressure                 :     105.7              63.3   bar
Flow                     :       3.750              3.750 ml/min

Detector Lamp Burn Times: Current On-Time  Accumulated On-Time
DAD 1, UV Lamp           :      30.02           59383.1    h
DAD 1, Visible Lamp      :      OFF             13046.5    h

Solvent Description      :
PMP1, Solvent A          : 0.1%TFA in Acn/H2O (2.5:97.5)
PMP1, Solvent B          : 0.1%TFA in AcN
PMP1, Solvent C          : 0.1%FA in Acn/H2O (2.5:97.5)
PMP1, Solvent D          : 0.1%FA in AcN
======================================================================
MSD parameters
Tune file name               :    C:\HPCHEM\1\1956ATUN\atunes.tun
Ionization mode              :    APCI

MSD Instrument Conditions    :      At Start         At Stop
Quad Temp                    :            99              99 C
Gas Temp                     :           350             350 C
Vaporizer                    :           349             352 C
RoughVac                     :             2               2 Torr
HighVac                      :      8.3E-006        8.3E-006 Torr
CapCur                       :           110             722 nA
ChamCur                      :             5               0 µA
CoronaVol                    :          4000              39 Volt
DryingGas                    :            10              10 l/min
Neb Pres                     :            50              50 psig
Turbo1Spd                    :            99              99 %
Turbo1Pwr                    :            99              98 W
RF Drive                     :      0.0E-001        0.0E-001 %
Qd TpDrv                     :             8               8 %
Gas TpDrv                    :            28              30 %
Vap TpDrv                    :            18              25 %
Neb PrDrv                    :            44              43 %
Gas FlDrv                    :             6               6 %
DelaySens                    :      0.0E-001        0.0E-001 V
Aux Input                    :      0.0E-001        0.0E-001 V
Other Det                    :      0.0E-001        0.0E-001 V
======================================================================
MSD tuning (calibration) parameters

Ionization polarity          :    Positive
Skim1                        :    Not Applicable
Skim2                        :    8.0 V
Ion Energy                   :    5.0 V
Lens1                        :    3.3 V
Lens2                        :    29 V
Iris                         :    -75 V
HED                          :    10000 V
Width Gain                   :    322
Width Offset                 :    Variable
                                  Mass         :   Value
                                  --------------------------
                                  121.05       :   -40
                                  622.03       :   -15
                                  922.01       :   -40
                                  --------------------------
Mass Gain                    :    30.25
Mass Offset                  :    0.654
Quad DC                      :    0.00 V
Octopole Peak                :    650 V
```

| | | |
|---|---|---|
| Octopole Knee | : | Not Applicable |
| Lens2DC | : | Not Applicable |
| L2RFEn | : | Not Applicable |
| L2RFPh | : | Not Applicable |
| L2RFAmp | : | Not Applicable |
| Mass Filter | : | Gaussian |
| Mass Filter Width | : | 0.30 Da |
| Time Filter | : | Gaussian |
| Time Filter Width | : | 0.030 minutes |

=====================================================================

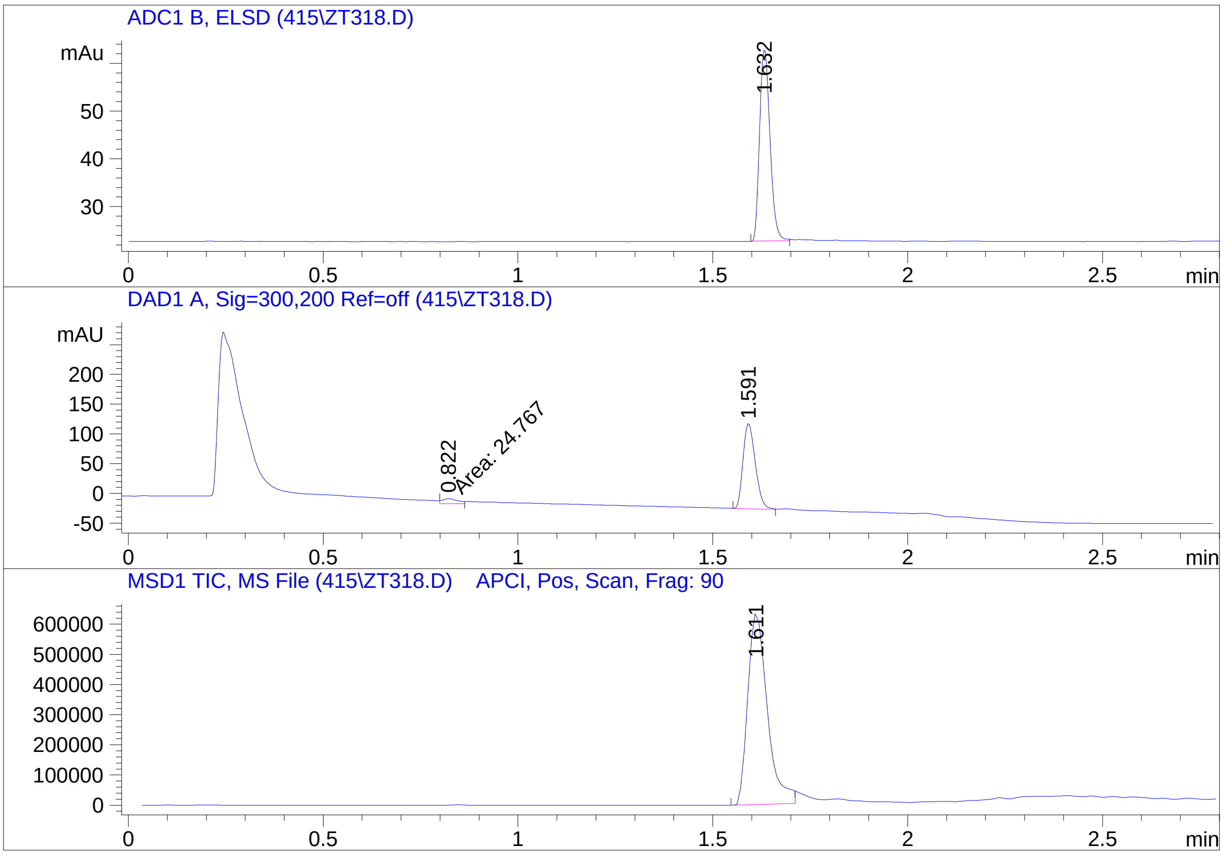


=====================================================================

Area Percent Report

=====================================================================

| | | |
|---|---|---|
| Sorted By | : | Signal |
| Multiplier | : | 1.0000 |
| Dilution | : | 1.0000 |

Use Multiplier & Dilution Factor with ISTDs

Signal 1: ADC1 B, ELSD

| Peak # | RetTime [min] | Type | Width [min] | Area [mAu*s] | Height [mAu] | Area % |
|---|---|---|---|---|---|---|
| 1 | 1.632 | PB | 0.0294 | 73.70155 | 40.21033 | 100.0000 |

Totals : 73.70155 40.21033

Signal 2: DAD1 A, Sig=300,200 Ref=off

| Peak # | RetTime [min] | Type | Width [min] | Area [mAU*s] | Height [mAU] | Area % |
|---|---|---|---|---|---|---|
| 1 | 0.822 | MM | 0.0460 | 24.76704 | 8.97513 | 7.2399 |
| 2 | 1.591 | BB | 0.0362 | 317.32370 | 141.26134 | 92.7601 |

Totals : 342.09074 150.23647

Signal 3: MSD1 TIC, MS File

| Peak # | RetTime [min] | Type | Width [min] | Area | Height | Area % |
|---|---|---|---|---|---|---|
| 1 | 1.611 | PB | 0.0546 | 2.21756e6 | 6.37157e5 | 100.0000 |

Totals : 2.21756e6 6.37157e5

=====================================================================

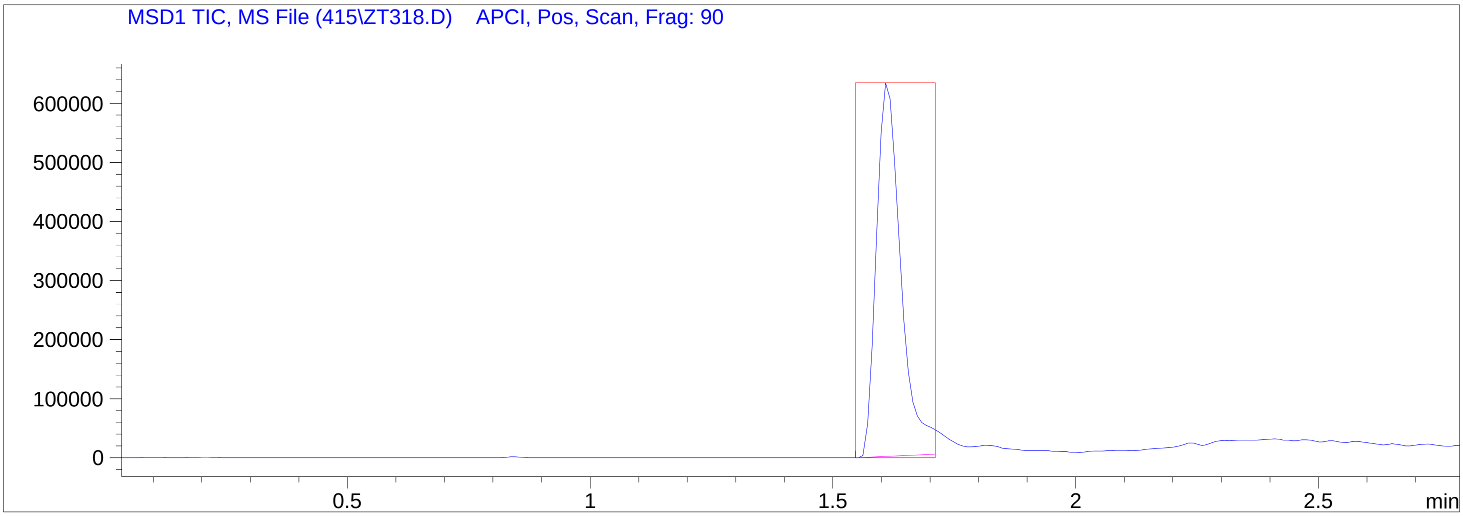


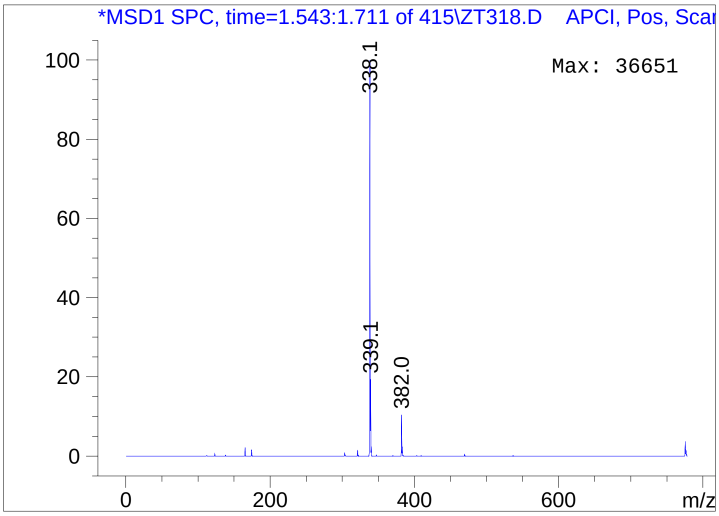


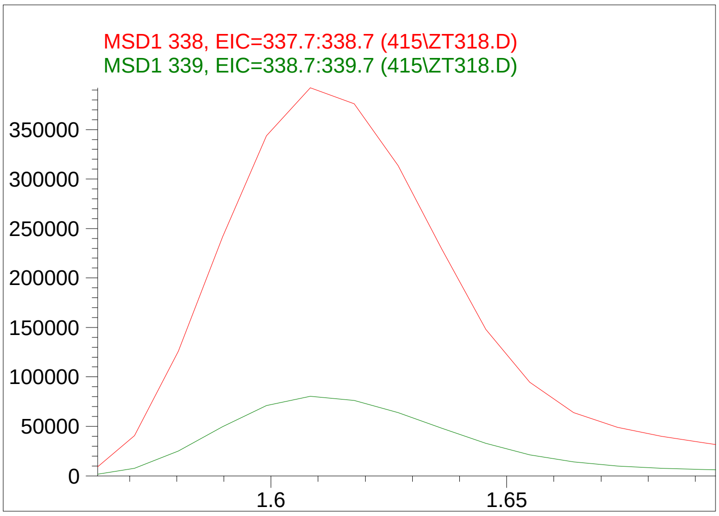


Peak #1 at 1.611 min ( 1.547 to 1.711 min)
-> The analysis found only one component, indicating a pure peak. <-

Component 1: Peak at Scan 170.2. Top ions are 338 339 382

---

*** End of Report ***

**12a (neogliptin)**

```
=====================================================================
Injection Date  : 05/06/2018 17:27:00            Seq. Line :  46
Sample Name     : TZ321                           Location : P1-C-02
Acq. Operator   : 6                                    Inj :   1
Acq. Instrument : Instrument 1                  Inj Volume : 3 µl
Different Inj Volume from Sequence !     Actual Inj Volume : 1.5 µl
Method          : C:\HPCHEM\1\METHODS\1PH.M
Last changed    : 25/05/2018 14:12:20  by 6
Column: Onyx Monolithic C18 50x4.6mm | 3.75ml/min | Columns Reg Valve
Gradient: "A"->@2.2min->"B"(Hold 0.4min)->@0.2min->"A"->PostRun
=====================================================================

Instrument Conditions   :    At Start           At Stop
Pressure                :     113.4              66.2   bar
Flow                    :       3.750             3.750 ml/min

Detector Lamp Burn Times: Current On-Time  Accumulated On-Time
DAD 1, UV Lamp          :       7.74          59438.6    h
DAD 1, Visible Lamp     :      OFF            13046.5    h

Solvent Description     :
PMP1, Solvent A         : 0.1%TFA in Acn/H2O (2.5:97.5)
PMP1, Solvent B         : 0.1%TFA in AcN
PMP1, Solvent C         : 0.1%FA in Acn/H2O (2.5:97.5)
PMP1, Solvent D         : 0.1%FA in AcN
=====================================================================
MSD parameters
Tune file name              :    C:\HPCHEM\1\1956ATUN\atunes.tun
Ionization mode             :    APCI

MSD Instrument Conditions   :      At Start         At Stop
Quad Temp                   :            99              99 C
Gas Temp                    :           350             350 C
Vaporizer                   :           348             354 C
RoughVac                    :             2               2 Torr
HighVac                     :      8.2E-006        8.1E-006 Torr
CapCur                      :           102             706 nA
ChamCur                     :             4               0 µA
CoronaVol                   :          3765              39 Volt
DryingGas                   :            10              10 l/min
Neb Pres                    :            50              50 psig
Turbo1Spd                   :            99              99 %
Turbo1Pwr                   :            97              95 W
RF Drive                    :      0.0E-001        0.0E-001 %
Qd TpDrv                    :             8               8 %
Gas TpDrv                   :            30              28 %
Vap TpDrv                   :            24              28 %
Neb PrDrv                   :            43              44 %
Gas FlDrv                   :             6               6 %
DelaySens                   :      0.0E-001        0.0E-001 V
Aux Input                   :      0.0E-001        0.0E-001 V
Other Det                   :      0.0E-001        0.0E-001 V
=====================================================================
MSD tuning (calibration) parameters

Ionization polarity         :    Positive
Skim1                       :    Not Applicable
Skim2                       :    8.0 V
Ion Energy                  :    5.0 V
Lens1                       :    3.3 V
Lens2                       :    29 V
Iris                        :    -75 V
HED                         :    10000 V
Width Gain                  :    322
Width Offset                :    Variable
                                 Mass          :   Value
                                 --------------------------
                                 121.05        :   -40
                                 622.03        :   -15
                                 922.01        :   -40
                                 --------------------------
Mass Gain                   :    30.25
Mass Offset                 :    0.654
Quad DC                     :    0.00 V
Octopole Peak               :    650 V
```

| | | |
|---|---|---|
| Octopole Knee | : | Not Applicable |
| Lens2DC | : | Not Applicable |
| L2RFEn | : | Not Applicable |
| L2RFPh | : | Not Applicable |
| L2RFAmp | : | Not Applicable |
| Mass Filter | : | Gaussian |
| Mass Filter Width | : | 0.30 Da |
| Time Filter | : | Gaussian |
| Time Filter Width | : | 0.030 minutes |

=====================================================================

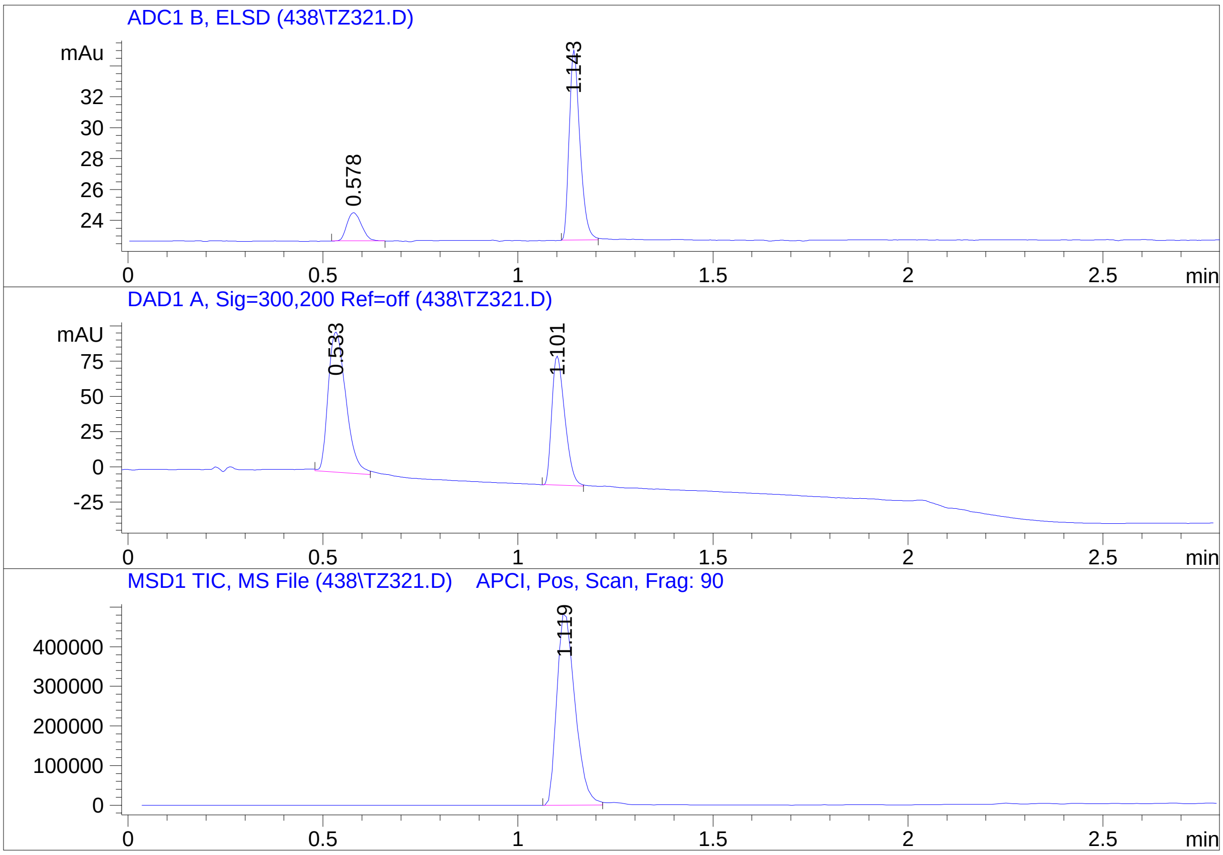


=====================================================================

Area Percent Report

=====================================================================

| | | |
|---|---|---|
| Sorted By | : | Signal |
| Multiplier | : | 1.0000 |
| Dilution | : | 1.0000 |

Use Multiplier & Dilution Factor with ISTDs

Signal 1: ADC1 B, ELSD

| Peak # | RetTime [min] | Type | Width [min] | Area [mAu*s] | Height [mAu] | Area % |
|---|---|---|---|---|---|---|
| 1 | 0.578 | PP | 0.0426 | 4.82976 | 1.83927 | 17.0634 |
| 2 | 1.143 | BB | 0.0293 | 23.47509 | 12.29206 | 82.9366 |
| Totals : | | | | 28.30484 | 14.13133 | |

Signal 2: DAD1 A, Sig=300,200 Ref=off

| Peak # | RetTime [min] | Type | Width [min] | Area [mAU*s] | Height [mAU] | Area % |
|---|---|---|---|---|---|---|
| 1 | 0.533 | BB | 0.0492 | 316.67227 | 99.23180 | 59.9573 |
| 2 | 1.101 | PB | 0.0373 | 211.49063 | 90.51962 | 40.0427 |
| Totals : | | | | 528.16290 | 189.75143 | |

Signal 3: MSD1 TIC, MS File

| Peak # | RetTime [min] | Type | Width [min] | Area | Height | Area % |
|---|---|---|---|---|---|---|
| 1 | 1.119 | PB | 0.0485 | 1.57950e6 | 4.93241e5 | 100.0000 |

Totals : 1.57950e6 4.93241e5

==================================================================

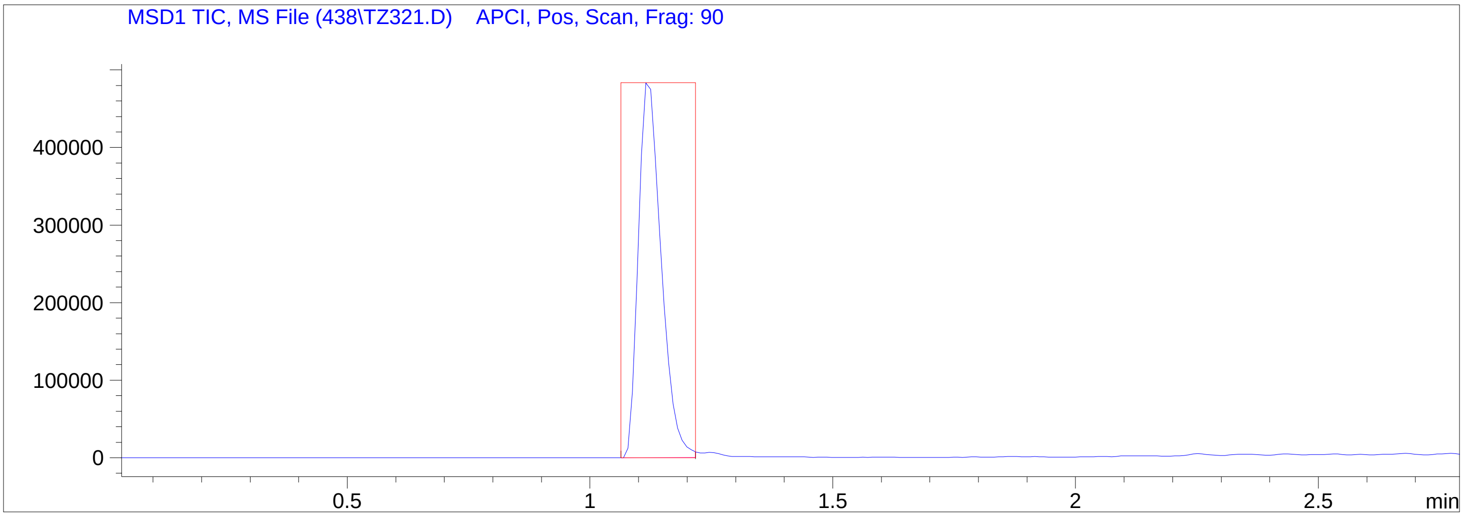


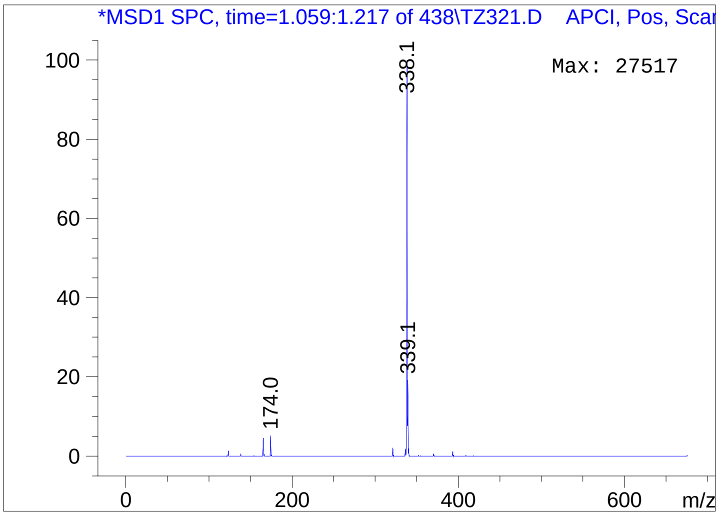


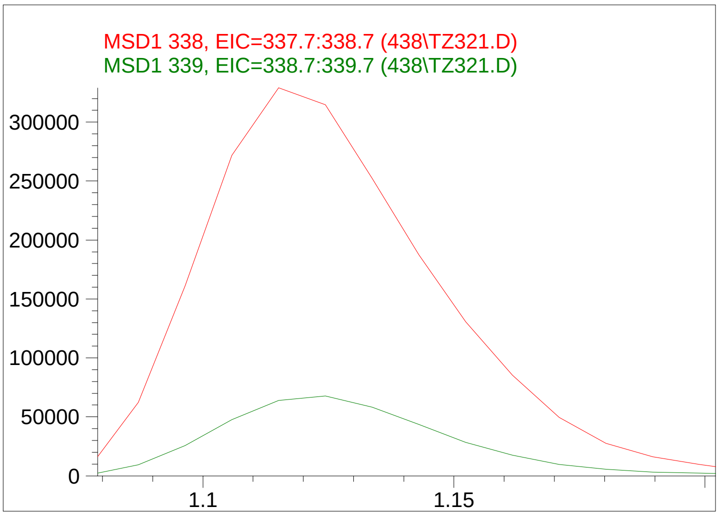


Peak #1 at 1.119 min ( 1.064 to 1.217 min)
-> The analysis found only one component, indicating a pure peak. <-

Component 1: Peak at Scan 117.5. Top ions are 338 339

---

*** End of Report ***

12ab

```
=====================================================================
Injection Date  : 31/05/2018 16:51:39          Seq. Line :  53
Sample Name     : ZT3142                        Location : P1-A-02
Acq. Operator   : 6                                  Inj :   1
Acq. Instrument : Instrument 1                Inj Volume : 3 µl
Different Inj Volume from Sequence !   Actual Inj Volume : 1.5 µl
Method          : C:\HPCHEM\1\METHODS\1PH.M
Last changed    : 25/05/2018 14:12:20  by 6
Column: Onyx Monolithic C18 50x4.6mm | 3.75ml/min | Columns Reg Valve
Gradient: "A"->@2.2min->"B"(Hold 0.4min)->@0.2min->"A"->PostRun
=====================================================================

Instrument Conditions   :    At Start          At Stop
Pressure                :     101.2             62.5   bar
Flow                    :       3.750            3.750 ml/min

Detector Lamp Burn Times: Current On-Time  Accumulated On-Time
DAD 1, UV Lamp          :      29.89           59382.9    h
DAD 1, Visible Lamp     :      OFF             13046.5    h

Solvent Description     :
PMP1, Solvent A         : 0.1%TFA in Acn/H2O (2.5:97.5)
PMP1, Solvent B         : 0.1%TFA in AcN
PMP1, Solvent C         : 0.1%FA in Acn/H2O (2.5:97.5)
PMP1, Solvent D         : 0.1%FA in AcN
=====================================================================
MSD parameters
Tune file name              :    C:\HPCHEM\1\1956ATUN\atunes.tun
Ionization mode             :    APCI

MSD Instrument Conditions   :      At Start         At Stop
Quad Temp                   :            99              99 C
Gas Temp                    :           350             350 C
Vaporizer                   :           350             350 C
RoughVac                    :             2               2 Torr
HighVac                     :      8.3E-006        8.3E-006 Torr
CapCur                      :           102             714 nA
ChamCur                     :             4               0 µA
CoronaVol                   :          4000              39 Volt
DryingGas                   :            10              10 l/min
Neb Pres                    :            50              50 psig
Turbo1Spd                   :            99              99 %
Turbo1Pwr                   :            96              98 W
RF Drive                    :      0.0E-001        0.0E-001 %
Qd TpDrv                    :             8               8 %
Gas TpDrv                   :            26              30 %
Vap TpDrv                   :            16              28 %
Neb PrDrv                   :            43              43 %
Gas FlDrv                   :             6               6 %
DelaySens                   :      0.0E-001        0.0E-001 V
Aux Input                   :      0.0E-001        0.0E-001 V
Other Det                   :      0.0E-001        0.0E-001 V
=====================================================================
MSD tuning (calibration) parameters

Ionization polarity         :    Positive
Skim1                       :    Not Applicable
Skim2                       :    8.0 V
Ion Energy                  :    5.0 V
Lens1                       :    3.3 V
Lens2                       :    29 V
Iris                        :    -75 V
HED                         :    10000 V
Width Gain                  :    322
Width Offset                :    Variable
                                 Mass         :   Value
                                 --------------------------
                                 121.05       :   -40
                                 622.03       :   -15
                                 922.01       :   -40
                                 --------------------------
Mass Gain                   :    30.25
Mass Offset                 :    0.654
Quad DC                     :    0.00 V
Octopole Peak               :    650 V
```

| | | |
|---|---|---|
| Octopole Knee | : | Not Applicable |
| Lens2DC | : | Not Applicable |
| L2RFEn | : | Not Applicable |
| L2RFPh | : | Not Applicable |
| L2RFAmp | : | Not Applicable |
| Mass Filter | : | Gaussian |
| Mass Filter Width | : | 0.30 Da |
| Time Filter | : | Gaussian |
| Time Filter Width | : | 0.030 minutes |

=====================================================================

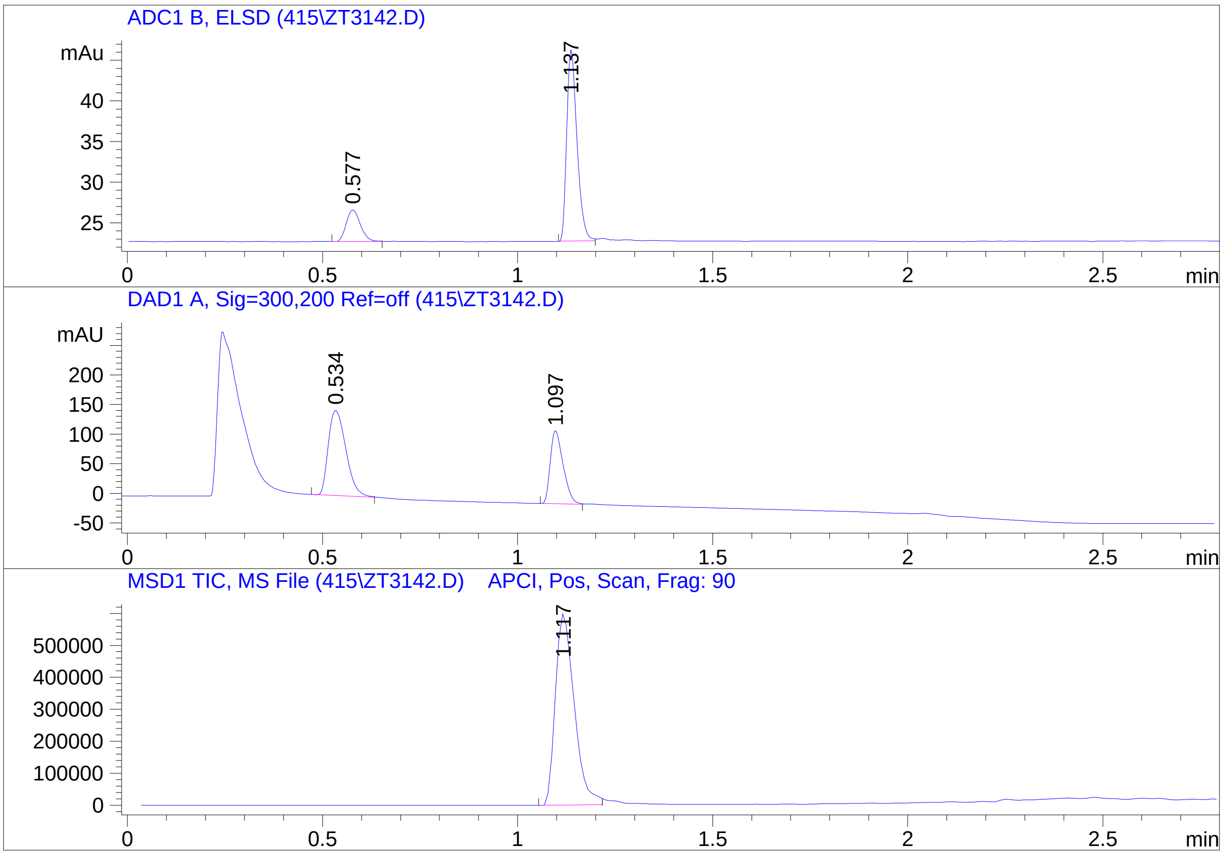


=====================================================================

Area Percent Report

=====================================================================

| | | |
|---|---|---|
| Sorted By | : | Signal |
| Multiplier | : | 1.0000 |
| Dilution | : | 1.0000 |

Use Multiplier & Dilution Factor with ISTDs

Signal 1: ADC1 B, ELSD

| Peak # | RetTime [min] | Type | Width [min] | Area [mAu*s] | Height [mAu] | Area % |
|---|---|---|---|---|---|---|
| 1 | 0.577 | BB | 0.0407 | 9.87186 | 3.87764 | 18.7429 |
| 2 | 1.137 | PB | 0.0282 | 42.79787 | 23.55371 | 81.2571 |

Totals : 52.66973 27.43136

Signal 2: DAD1 A, Sig=300,200 Ref=off

| Peak # | RetTime [min] | Type | Width [min] | Area [mAU*s] | Height [mAU] | Area % |
|---|---|---|---|---|---|---|
| 1 | 0.534 | PB | 0.0507 | 450.84891 | 143.49112 | 61.2216 |
| 2 | 1.097 | PB | 0.0374 | 285.57281 | 121.57243 | 38.7784 |

Totals : 736.42172 265.06355

Signal 3: MSD1 TIC, MS File

| Peak # | RetTime [min] | Type | Width [min] | Area | Height | Area % |
|---|---|---|---|---|---|---|
| 1 | 1.117 | PB | 0.0531 | 2.02029e6 | 6.02064e5 | 100.0000 |

Totals : 2.02029e6 6.02064e5

================================================================================

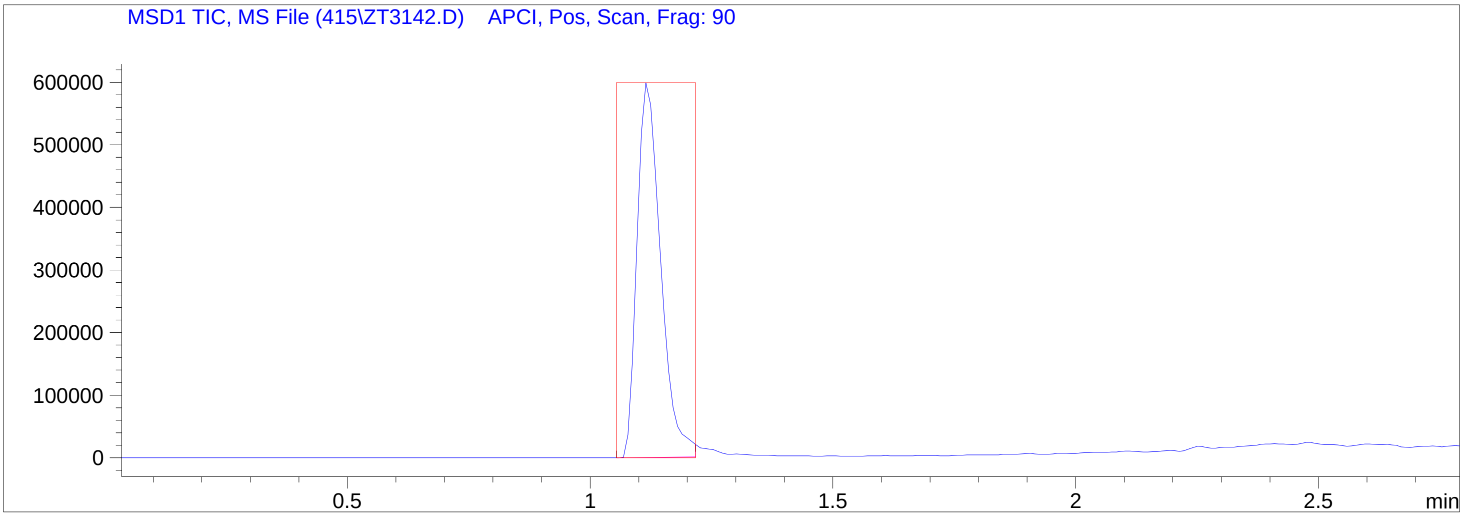


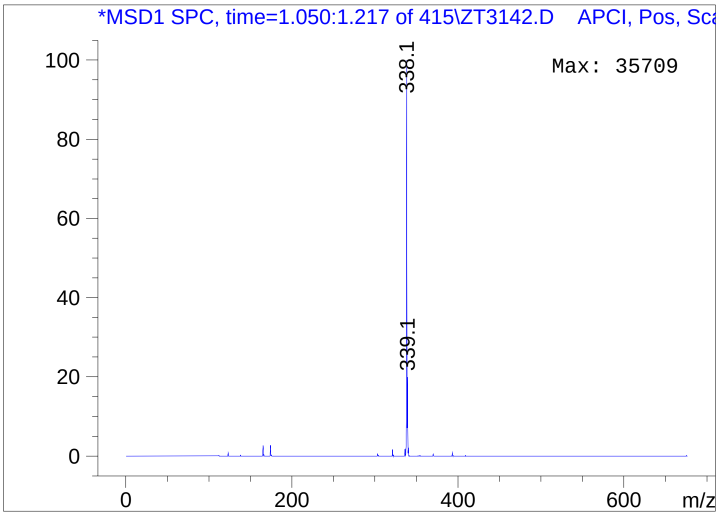


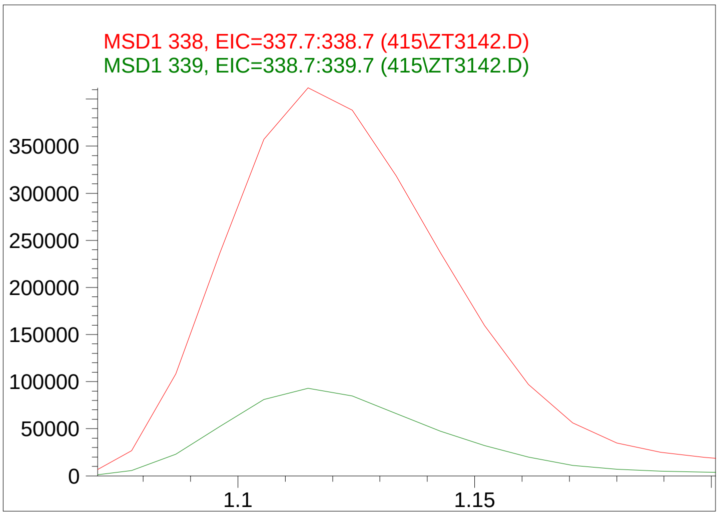


Peak #1 at 1.117 min ( 1.054 to 1.217 min)
-> The analysis found only one component, indicating a pure peak. <-

Component 1: Peak at Scan 117.1. Top ions are 338 339

---

*** End of Report ***

12cd

```
=====================================================================
Injection Date  : 05/06/2018 17:31:08           Seq. Line :  47
Sample Name     : TZ322                          Location : P1-D-02
Acq. Operator   : 6                                   Inj :   1
Acq. Instrument : Instrument 1                 Inj Volume : 3 µl
Different Inj Volume from Sequence !    Actual Inj Volume : 1.5 µl
Method          : C:\HPCHEM\1\METHODS\1PH.M
Last changed    : 25/05/2018 14:12:20  by 6
Column: Onyx Monolithic C18 50x4.6mm | 3.75ml/min | Columns Reg Valve
Gradient: "A"->@2.2min->"B"(Hold 0.4min)->@0.2min->"A"->PostRun
=====================================================================

Instrument Conditions   :    At Start          At Stop
Pressure                :     112.1             66.3   bar
Flow                    :       3.750            3.750 ml/min

Detector Lamp Burn Times: Current On-Time  Accumulated On-Time
DAD 1, UV Lamp          :       7.81          59438.7   h
DAD 1, Visible Lamp     :      OFF            13046.5   h

Solvent Description     :
PMP1, Solvent A         : 0.1%TFA in Acn/H2O (2.5:97.5)
PMP1, Solvent B         : 0.1%TFA in AcN
PMP1, Solvent C         : 0.1%FA in Acn/H2O (2.5:97.5)
PMP1, Solvent D         : 0.1%FA in AcN
=====================================================================
MSD parameters
Tune file name              :    C:\HPCHEM\1\1956ATUN\atunes.tun
Ionization mode             :    APCI

MSD Instrument Conditions   :      At Start        At Stop
Quad Temp                   :            99            100 C
Gas Temp                    :           350            350 C
Vaporizer                   :           350            352 C
RoughVac                    :             2              2 Torr
HighVac                     :      8.2E-006       8.2E-006 Torr
CapCur                      :           110             94 nA
ChamCur                     :             4              4 µA
CoronaVol                   :          3843           3647 Volt
DryingGas                   :            10             10 l/min
Neb Pres                    :            50             50 psig
Turbo1Spd                   :            99             99 %
Turbo1Pwr                   :            95             96 W
RF Drive                    :             0             49 %
Qd TpDrv                    :             9              8 %
Gas TpDrv                   :            26             26 %
Vap TpDrv                   :            22             23 %
Neb PrDrv                   :            44             43 %
Gas FlDrv                   :             6              6 %
DelaySens                   :      0.0E-001       0.0E-001 V
Aux Input                   :      0.0E-001       0.0E-001 V
Other Det                   :      0.0E-001       0.0E-001 V
=====================================================================
MSD tuning (calibration) parameters

Ionization polarity         :    Positive
Skim1                       :    Not Applicable
Skim2                       :    8.0 V
Ion Energy                  :    5.0 V
Lens1                       :    3.3 V
Lens2                       :    29 V
Iris                        :    -75 V
HED                         :    10000 V
Width Gain                  :    322
Width Offset                :    Variable
                                 Mass          :   Value
                                 --------------------------
                                 121.05        :   -40
                                 622.03        :   -15
                                 922.01        :   -40
                                 --------------------------
Mass Gain                   :    30.25
Mass Offset                 :    0.654
Quad DC                     :    0.00 V
Octopole Peak               :    650 V
```

| | | |
|---|---|---|
| Octopole Knee | : | Not Applicable |
| Lens2DC | : | Not Applicable |
| L2RFEn | : | Not Applicable |
| L2RFPh | : | Not Applicable |
| L2RFAmp | : | Not Applicable |
| Mass Filter | : | Gaussian |
| Mass Filter Width | : | 0.30 Da |
| Time Filter | : | Gaussian |
| Time Filter Width | : | 0.030 minutes |

=====================================================================

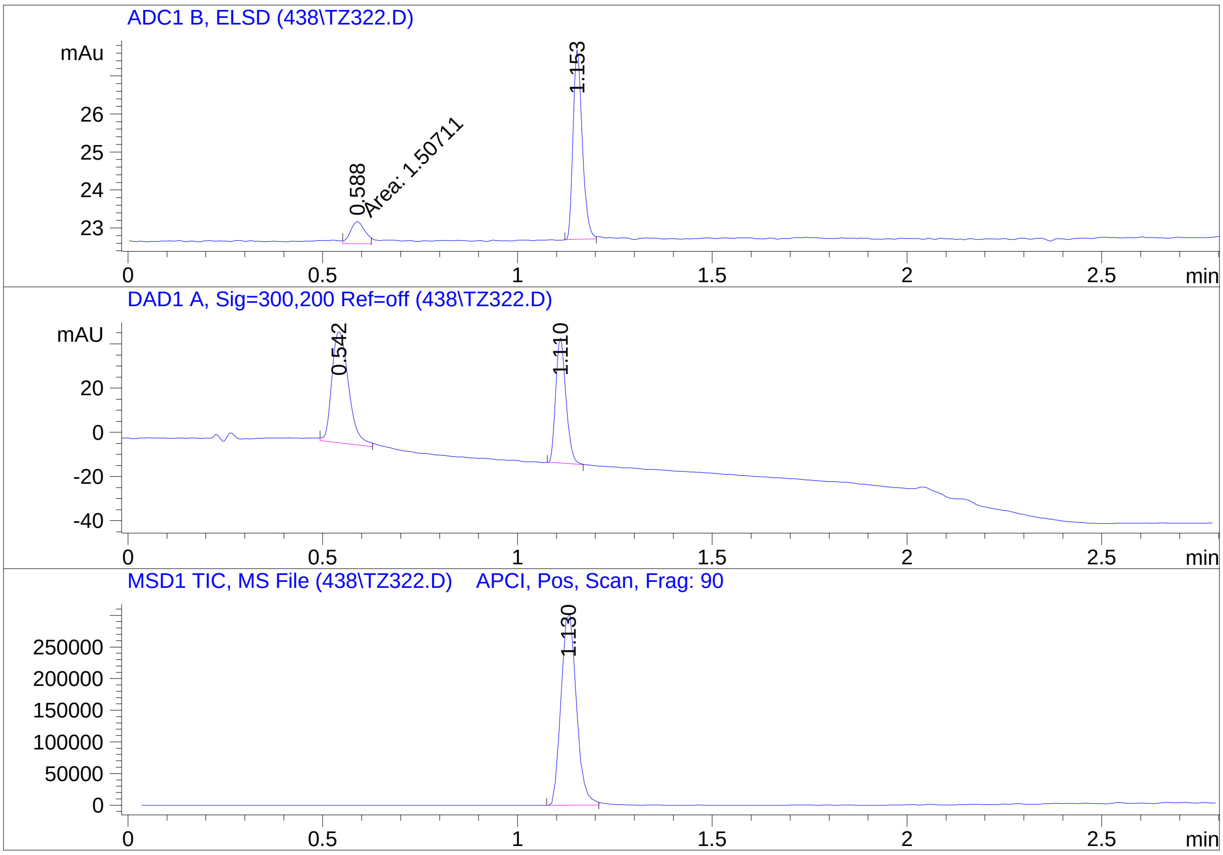


=====================================================================

Area Percent Report

=====================================================================

| | | |
|---|---|---|
| Sorted By | : | Signal |
| Multiplier | : | 1.0000 |
| Dilution | : | 1.0000 |

Use Multiplier & Dilution Factor with ISTDs

Signal 1: ADC1 B, ELSD

| Peak # | RetTime [min] | Type | Width [min] | Area [mAu*s] | Height [mAu] | Area % |
|---|---|---|---|---|---|---|
| 1 | 0.588 | MM | 0.0431 | 1.50711 | 5.82786e-1 | 15.3865 |
| 2 | 1.153 | PB | 0.0264 | 8.28787 | 4.98131 | 84.6135 |

Totals : 9.79498 5.56410

Signal 2: DAD1 A, Sig=300,200 Ref=off

| Peak # | RetTime [min] | Type | Width [min] | Area [mAU*s] | Height [mAU] | Area % |
|---|---|---|---|---|---|---|
| 1 | 0.542 | BB | 0.0471 | 150.76880 | 50.16784 | 59.2738 |
| 2 | 1.110 | PB | 0.0295 | 103.59119 | 56.12290 | 40.7262 |

Totals : 254.35999 106.29074

Signal 3: MSD1 TIC, MS File

| Peak # | RetTime [min] | Type | Width [min] | Area | Height | Area % |
|---|---|---|---|---|---|---|
| 1 | 1.130 | PB | 0.0427 | 8.30184e5 | 3.07168e5 | 100.0000 |

Totals : 8.30184e5 3.07168e5

==================================================================

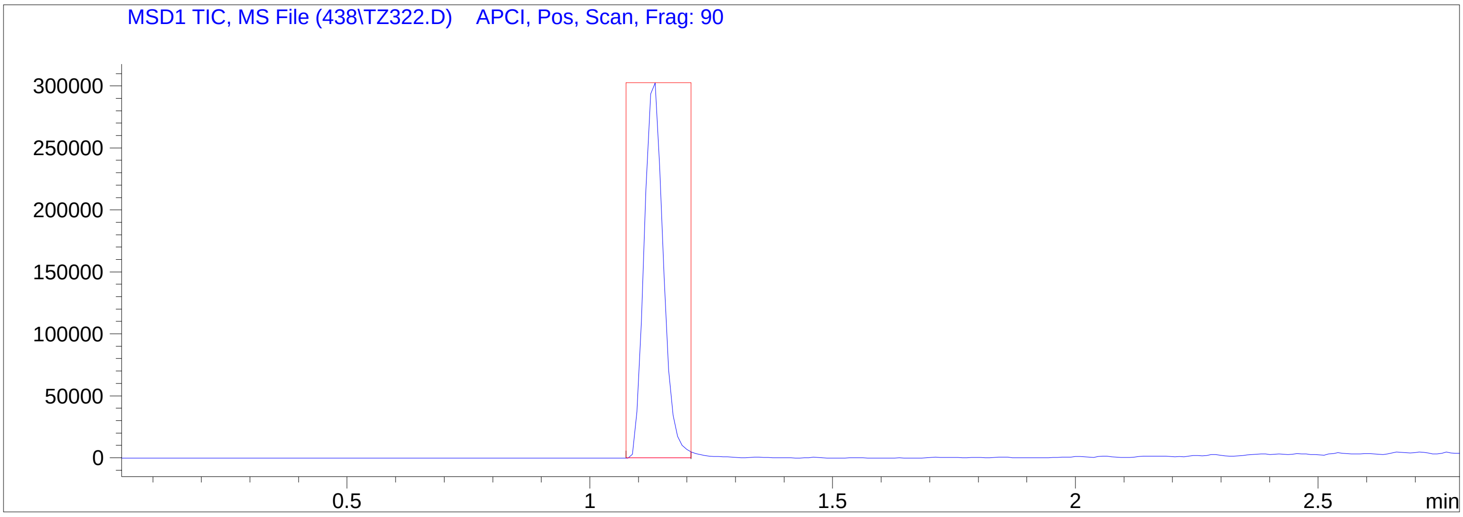


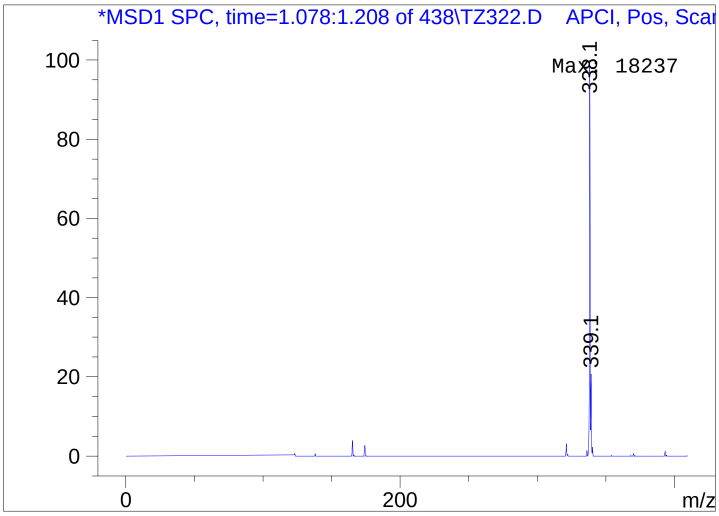


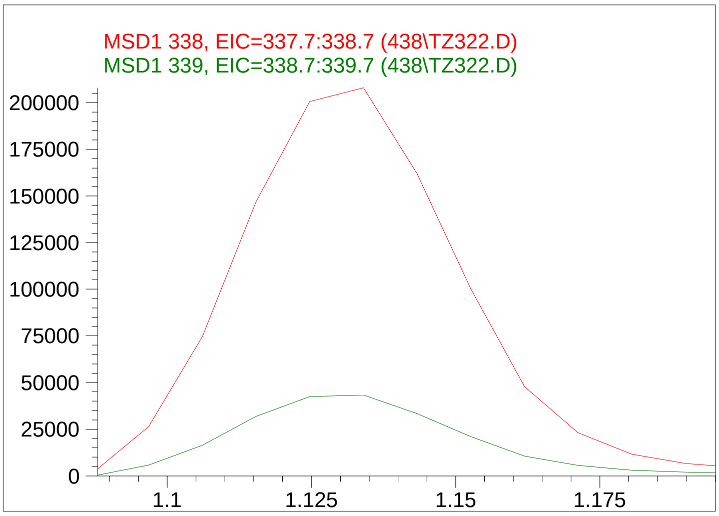


Peak #1 at  1.130 min ( 1.075 to  1.208 min)
-> The analysis found only one component, indicating a pure peak. <-

Component 1:  Peak at Scan 118.6. Top ions are  338 339

---

*** End of Report ***
